# Structural Investigation and Molecular Modeling Studies of Strobilurin-Based Fungicides Active against the Rice Blast Pathogen *Pyricularia oryzae*

**DOI:** 10.3390/ijms22073731

**Published:** 2021-04-02

**Authors:** Andrea Kunova, Luca Palazzolo, Fabio Forlani, Giorgia Catinella, Loana Musso, Paolo Cortesi, Ivano Eberini, Andrea Pinto, Sabrina Dallavalle

**Affiliations:** 1 Department of Food, Environmental and Nutritional Science (DeFENS), University of Milan, 20133 Milan, Italy; andrea.kunova@unimi.it (A.K.); fabio.forlani@unimi.it (F.F.); giorgia.catinella@unimi.it (G.C.); loana.musso@unimi.it (L.M.); paolo.cortesi@unimi.it (P.C.); sabrina.dallavalle@unimi.it (S.D.); 2 Department of Pharmacological and Biomolecular Sciences (DiSFeB), University of Milan, 20133 Milan, Italy; luca.palazzolo@unimi.it; 3 Data Science Research Center (DSRC), University of Milan, 20133 Milan, Italy

**Keywords:** rice blast, crop protection, cytochrome *bc1* complex, antifungal compounds, homology modeling, molecular docking

## Abstract

The increasing emergence of fungicide-resistant pathogens requires urgent solutions for crop disease management. Here, we describe a structural investigation of new fungicides obtained by combining strobilurin and succinate dehydrogenase inhibitor pharmacophores. We identified compounds endowed with very good activity against wild-type *Pyricularia oryzae*, combined in some cases with promising activity against strobilurin-resistant strains. The first three-dimensional model of *P. oryzae* cytochrome *bc1* complex containing azoxystrobin as a ligand was developed. The model was validated with a set of commercially available strobilurins, and it well explains both the resistance mechanism to strobilurins mediated by the mutation G143A and the activity of metyltetraprole against strobilurin-resistant strains. The obtained results shed light on the key recognition determinants of strobilurin-like derivatives in the cytochrome *bc1* active site and will guide the further rational design of new fungicides able to overcome resistance caused by G143A mutation in the rice blast pathogen.

## 1. Introduction

The mitochondrial respiratory chain is one of the most important targets for the development of agricultural fungicides [1]. Different fungicide classes act on enzymes within the respiratory chain, the most important being succinate dehydrogenase inhibitors (SDHI, or complex II) and strobilurins (quinone outside inhibitors, QoI, or complex III) [2,3,4,5,6]. Strobilurins interact with the cytochrome *bc1* complex (complex III) involved in electron transfer, oxidation of hydroquinone, and reduction of cytochrome *c* in the mitochondrial respiratory chain. Strobilurin fungicides have been a milestone in the fungicide market, and they are still among the best-selling agrochemicals worldwide. Succinate dehydrogenase (SDH), on the other side, is a particular enzyme on the interface between the tricarboxylic acid cycle (TCA) and the mitochondrial respiratory chain. It couples the oxidation of succinate to fumarate in TCA with the reduction of ubiquinone to ubiquinol in the electron transfer chain. SDHI are the fastest growing class of fungicides in terms of newly discovered products [7].

Notwithstanding their enormous potential, both fungicide classes have a single-site mode of action, which makes them prone to resistance development in fungal pathogen populations. The resistance to strobilurins is most often determined by a single amino acid substitution from glycine to alanine (G143A) and until now has been detected in approximately 50 different pathogens, while diverse mutations in the B, C, and D subunits of the SDH enzyme are known in ca. 20 fungal species [8,9,10]. Strict anti-resistance measures are being applied particularly to strobilurin use to ensure their long-lasting activity and to delay the spread of fungicide resistance, e.g., limited number of treatments, preferably in a mixture or rotation with fungicides having a different mode of action [11].

Rice blast, caused by the fungus *Pyricularia oryzae*, is one of the most serious fungal diseases of cultivated rice worldwide, causing each year 10–30% yield losses corresponding to ca. 70 billion USD of economic loss [12,13,14]. It is managed predominantly by fungicides, especially in regions where traditional rice blast-susceptible varieties are grown, and breeding of resistant varieties has been neglected. During the years, diverse fungicide classes have been used against *P. oryzae*, among which *β*-tubulin inhibitors (e.g., benomyl), sterol biosynthesis inhibitors (e.g., propiconazole), melanin biosynthesis inhibitors (e.g., tricyclazole), antibiotics (e.g., kasugamycin), or QoI/strobilurins (e.g., azoxystrobin) [15,16]. In Europe and particularly in Italy, only strobilurins and demethylation inhibitors (DMI) are currently approved [17]. Therefore, to augment the portfolio of fungicides for rice blast management and to delay/overcome the resistance development against at-risk fungicides, new solutions are urgently needed.

**Figure 1:**
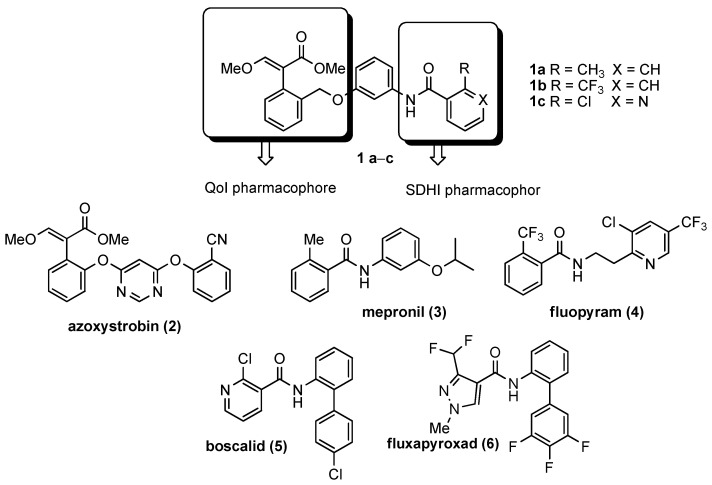
First generation of dual compounds **1a–c** [18], commercial strobilurins (**2**), and succinate dehydrogenase inhibitors (SDHIs) (**3**–**6**), chosen as templates.

In the search for innovative antifungal treatments, we reported in a recent paper the development of new hybrid fungicides obtained by combining the pharmacophore features of QoI and SDHI [18]. The first-generation hybrid compounds (Figure 1, **1a–c**) featured the three ring-based structure of azoxystrobin (**2**) and contained the methyl-(*E*)-*β*-methoxyacrylate strobilurin pharmacophore connected by a proper linker to the carboxamide pharmacophore of three commercial SDHI inhibitors, e.g., mepronil (**3**), fluopyram (**4**), and boscalid (**5**). The pilot hybrid compounds showed good in vitro mycelium growth inhibition of the rice blast pathogen, *Pyricularia oryzae*; however, they almost completely lost their activity on strobilurin-resistant strains.

The major goal of this work was to modify the structure of the most promising candidate **1a** in order to obtain highly active compounds, possibly capable of overcoming strobilurin resistance in *P. oryzae*.

The strobilurin pharmacophore was maintained unaltered, as it was previously found to interact very strongly with the enzyme. Instead, attention was focused on the following aspects:

1. Spatial orientation of the two pharmacophores
2. Role and nature of the linker
3. Nature and substitution pattern of the SDHI pharmacophore.

## 2. Results

**Figure 2:**
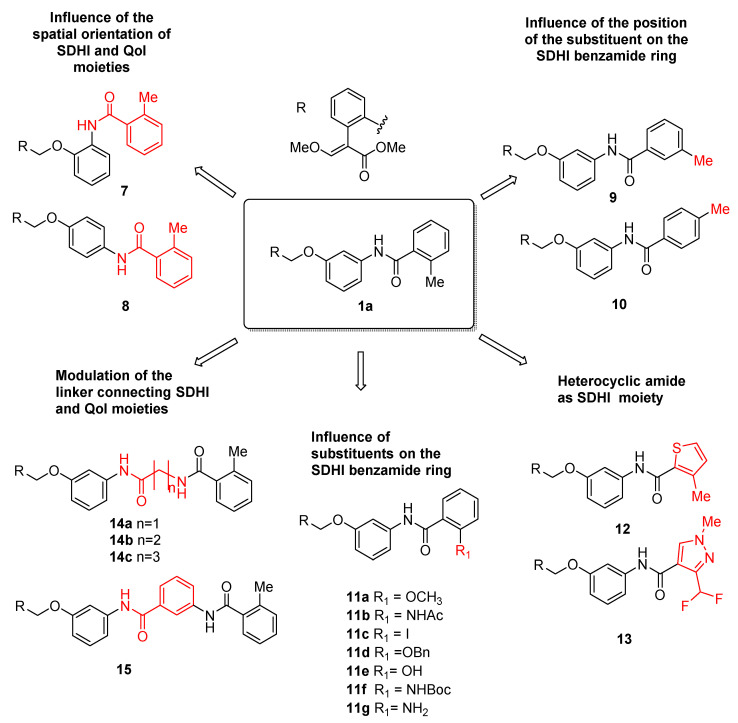
Design strategy to optimize the structure of compound **1a** to obtain new dual SDHI-QoI (**7**–**15**).

To address the objectives described above, we synthesized compounds reported in Figure 2. Initially, we investigated the influence of the orientation of the two pharmacophore moieties. For this purpose, we prepared derivatives **7** and **8** with SDHI and QoI pharmacophores positioned in ortho and para relative positions on the central ring, respectively (Figure 2). Moreover, we varied the position of the substituent (methyl group) on the benzamide ring (compounds **9** and **10**).

Successively, we investigated the effect of the introduction of diverse substituents on the benzamide ring. Thus, we prepared compounds **11a**–**g** bearing substituents with different steric and stereoelectronic properties.

After this, we changed the nature of the aromatic ring in the SDHI moiety, replacing the benzamide with a heterocyclic amide (compounds **12** and **13**). Compound **13** is featured with the pyrazole carboxamide found in the commercial SDHI inhibitor fluxapyroxad (**6**, Figure 1).

Finally, we focused our attention on the linker between the two pharmacophores. In compounds **14a–c**, we increased the distance between the strobilurin and SDHI moieties, using glycine, *β*-alanine, and γ-aminobutyric acid (GABA) as linkers. In addition, a further aromatic ring was placed between the two active moieties in compound **15** (Figure 2).

### 2.1. Chemistry

**Scheme 1:**
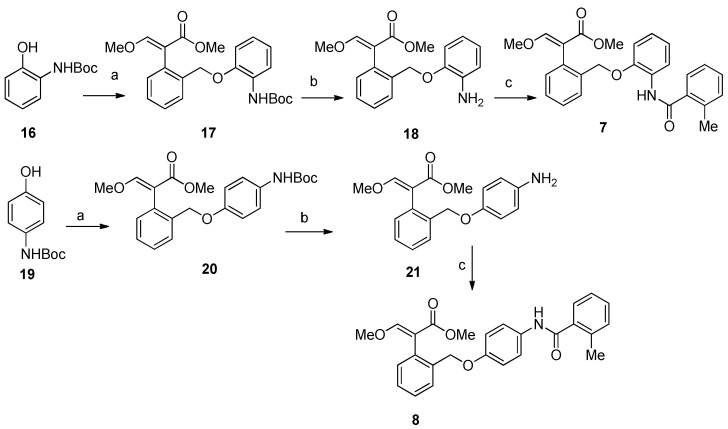
Synthesis of compounds **7–8**. Reagents and conditions: (**a**) K_2_CO_3_, (E)-methyl 2-(2-(bromomethyl)phenyl)-3-methoxyacrylate, 18-crown-6, acetone, 3 h, reflux, 23% for **17,** and 64% for **20**; (**b**) TFA, CH_2_Cl_2_, 3 h, 0 °C, 98% for **18,** and 95% for **21**; (**c**) 2-methylbenzoic acid, EDC·HCl, HOBt, DIPEA, CH_2_Cl_2_, 24 h, rt, 35% for **7,** and 64% for **8**.

Compound **7** was prepared by reacting (E)-methyl 2-(2-(bromomethyl)phenyl)-3-methoxyacrylate [18] with tert-butyl 2-hydroxyphenylcarbamate **16**, followed by removal of the Boc protecting group and acylation of the resulting compound **18** with 2-methylbenzoic acid. The same synthetic strategy was used to obtain the isomer **8** starting from tert-butyl 4-hydroxyphenylcarbamate **19** (Scheme 1).

**Scheme 2:**
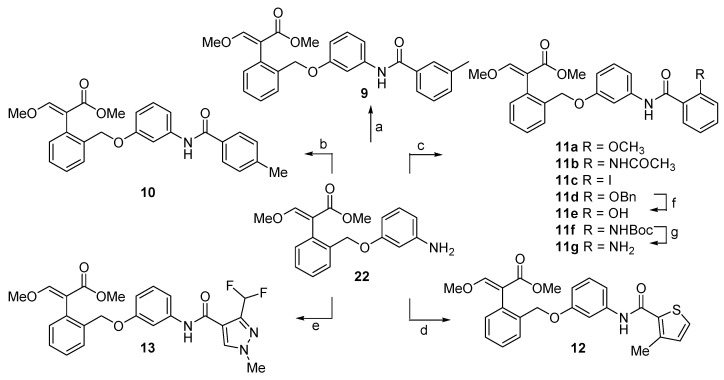
Synthesis of compounds **9–13**. Reagents and conditions: (**a**) 3-methylbenzoic acid, EDC·HCl, HOBt, DIPEA, CH_2_Cl_2_, 0 °C to rt 84%; (**b**) 4-methylbenzoic acid, EDC·HCl, HOBt, DIPEA, CH_2_Cl_2_, 0°C to rt 82%; (**c**) for **11a**: 2-methoxybenzoic acid, EDC·HCl, HOBt, DIPEA, CH_2_Cl_2_, 0 °C to rt, 64%; for **11b**: 2-acetamidobenzoic acid, EDC·HCl, HOBt, DIPEA, CH_2_Cl_2_, 0 °C to rt; 59%; for **11c**: 2-iodobenzoic acid, EDC·HCl, HOBt, DIPEA, CH_2_Cl_2_, 0 °C to rt, 86%, for **11d**: 2-benzyloxybenzoic acid, EDC·HCl, HOBt, DIPEA, CH_2_Cl_2_, 0 °C to rt, 71%, for **11f**: 2-tert-butoxycarbomylaminobenzoic acid, EDC·HCl, HOBt, DIPEA, CH_2_Cl_2_, 0 °C to rt, 41%; (**d**) 3-methylthiophene-2-carboxylic acid, EDC·HCl, HOBt, DIPEA, CH_2_Cl_2_, 0 °C to rt, 62%; (**e**) 3-(difluoromethyl)-1-methyl-1H-pyrazole-4-carboxylic acid, EDC·HCl, HOBt, DIPEA, CH_2_Cl_2_, 0 °C to rt, 57%; (**f**) 10% Pd/C, H_2_, ethyl acetate, rt, overnight, 91%; (**g**) TFA, CH_2_Cl_2_, 2 h, rt, 52%.

The synthesis of compounds **9–13** started from the common precursor **22 [18]**, which was coupled with the suitable carboxylic acid using EDC·HCl and HOBt as coupling reagents and DIPEA at 0 °C (Scheme 2).

**Scheme 3:**
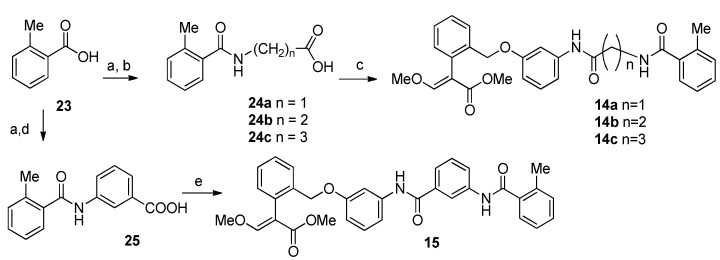
Synthesis of compounds **14–15**. Reagents and conditions: (**a**) (COCl)_2_, CH_2_Cl_2_, DMF, 0 °C, rt, 63%; (**b**) for **24a**: glycine, 2M NaOH, 8 h, rt, 19%; for **24b**: *β*-alanine, 2M NaOH, overnight, rt, 24%; for **24c**: GABA, 2M NaOH, 2 h at 0 °C then overnight at rt, 41%; (**c**) i. EDC·HCl, HOBt, CH_2_Cl_2_, 30′ at 0 °C; ii. **22**, DIPEA, CH_2_Cl_2_, at 0 °C then overnight at rt, for **14a**: 18%, for **14b**: 49%, for **14c**: 33%; (**d**) 3-aminobenzoic acid, TEA, CHCl_3_, 51%; (**e**) i. EDC·HCl, HOBt, CH_2_Cl_2_, 30′ at 0 °C; ii. **22**, DIPEA, CH_2_Cl_2_, at 0 °C then overnight at rt, 33%.

Compounds **14a–c** were prepared starting from 2-methylbenzoic acid **23**, which was condensed with glycine, *β*-alanine, and GABA, respectively, to obtain intermediate acids **24a–c**. Coupling with the amine **22** in the presence of EDC and HOBt provided the desired compounds **14a–c**. Compound **15** was obtained by reaction of **23** with 3-aminobenzoic acid to give **25**, which was then condensed with **22** following the protocol described above (Scheme 3).

### 2.2. Biological Activity

**Figure 3:**
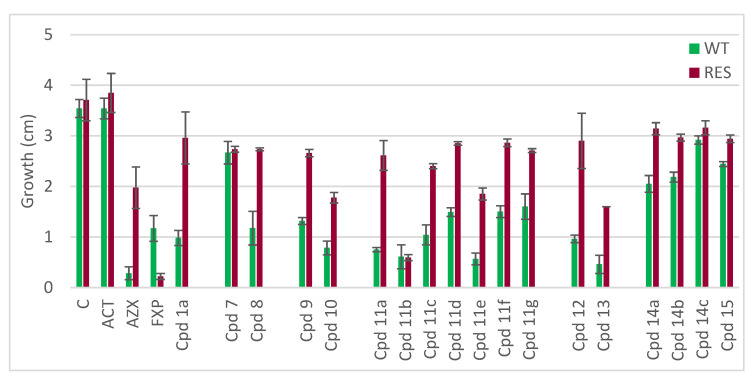
Mycelium growth of wild-type (WT) and QoI-resistant (RES) *Pyricularia oryzae* strains on control malt-extract agar media (C = control, ACT = 1% acetone) and media supplemented with azoxystrobin (AZX), fluxapyroxad (FXP), or dual compounds (Cpds **1a**, **7–15**) at a concentration of 25 mg/L. Error bars represent the standard deviation of the mean.

The inhibitory activity of the novel compounds **7–15** on the mycelium growth of *P. oryzae* was evaluated and compared to the activity of the reference compound **1a** (Figure 3, Table 1). Acetone (ACT), used as a solvent, did not show any inhibitory activity on *P. oryzae* mycelium growth. The commercial fungicides azoxystrobin (AZX; QoI) and fluxapyroxad (FXP, SDHI) and the newly synthesized compounds were tested at the final concentration of 25 mg/L. AZX inhibited > 90% of the growth of wild-type (WT) strains, while the strobilurin-resistant (RES) strains were inhibited to < 50%. On the contrary, FXP (SDHI) showed low activity on WT strains (65% inhibition) but > 90% inhibition of RES strains.

### 2.3. SDH and Qo Inhibitory Activity of Compound ***11b***

As compound **11b** showed very promising biological activity on both WT and RES strains of *P. oryzae* (>80% inhibition), we hypothesized that it maintained good Qo and SDH inhibitory activity. The measurement of decylubiquinol:Cyt *c* reductase activity (i.e., the Cyt *bc1* complex activity) was performed to evaluate the QoI action of the selected compound. To this purpose, the reduction rate of Cyt *c* mediated by the mitochondrial fraction of *P. oryzae* was measured using decylubiquinol (DBH_2_) as the electron-donor substrate. The DBH_2_: Cyt *c* reductase activity was inhibited by 58 ± 18% by the compound **11b** (50 µM). A similar inhibition value (61 ± 10%) was observed for azoxystrobin (50 µM), thus confirming that **11b** retained the QoI action.

**Figure 4:**
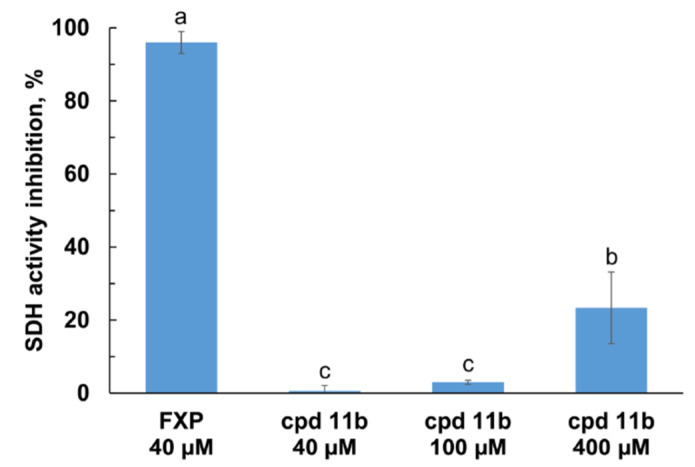
Effect of compound **11b** on the *P. oryzae* mitochondrial SDH activity. To evaluate the percent of inhibition due to the SDHI action, the rates of the succinate:2,3-dimethoxy-5-methyl-*p*-benzoquinone dehydrogenase activity of the mitochondrial fraction were measured at 25 °C (λ = 595 nm) using DCPIP in the presence of **11b** and the reference compound, fluxapyroxad (FXP), at the indicated concentrations. Data represent the mean ± standard deviation of at least three independent trials. Different superscript letters indicate statistically significant differences (Tukey HSD, *p* ≤ 0.01).

SDH enzymatic assay was performed measuring the electron transfer rate mediated by the mitochondrial fraction of *P. oryzae* in the presence of 2,3-dimethoxy-5-methyl-*p*-benzoquinone and succinate by a colorimetric method, using the redox dye 2,6-dichlorophenolindophenol (DCPIP, Figure 4).

Fluxapyroxad showed > 90% inhibition of SDH activity at the concentration of 40 µM. Surprisingly, at the same concentration, no SDH inhibition was observed for compound **11b**. By increasing the concentration to 400 µM, approximately 23% inhibition of the SDH enzyme activity was observed.

### 2.4. In Silico Modeling and Docking

**Figure 5:**
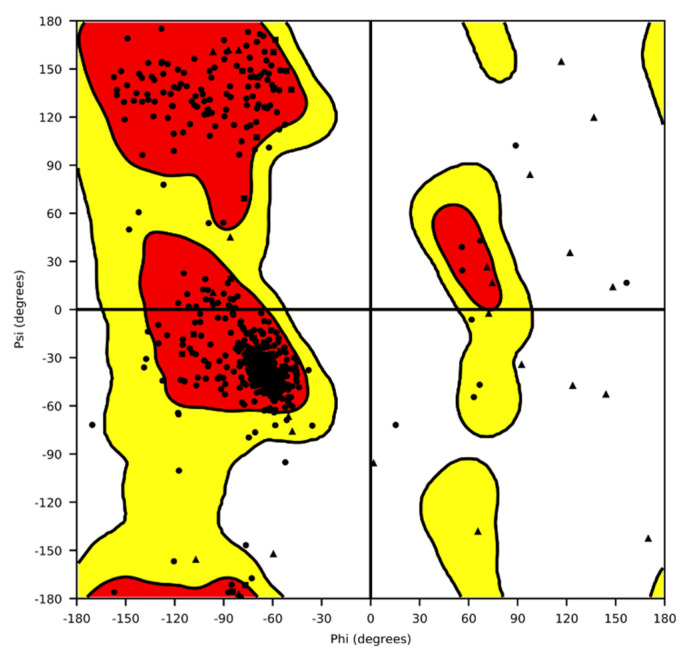
Ramachandran plot of the three-dimensional model for *Pyricularia oryzae* cytochrome *bc1* complex. Red and yellow regions are respectively favored and allowed regions. Triangles refer to G, squares to P, and dots to the remaining amino acids.

Since no experimental structures for *P. oryzae* cytochrome *bc1* in complex with azoxystrobin are available, we produced a 3D model by homology. The Ramachandran plot (Figure 5), the side-chain packing, and the stereochemical quality were carefully checked to verify that all these parameters were suitable and consistent with typical values found in template crystallographic structures.

**Figure 6:**
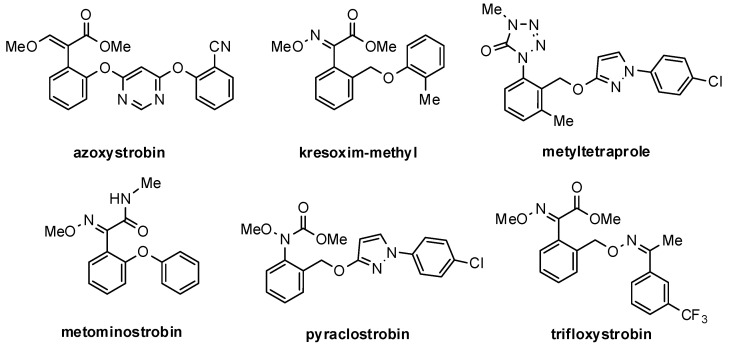
Validation set of marketed strobilurins and metyltetraprole.

The final model contained azoxystrobin that was transferred by the selected template (PDB ID: 1SQB). The selected docking procedure, thoroughly described in the Materials and Methods section, was validated testing well-known strobilurin QoI: azoxystrobin, trifloxystrobin, kresoxim-methyl, and metominostrobin, characterized by the presence of a *β*-methoxyacrylate, *α*-methoxyimino acetate, or *α*-methoxyimino-*N*-methylacetamide pharmacophore, respectively (Figure 6). Metyltetraprole, a newly discovered strobilurin derivative insensitive to G143A mutation and thus active also against strobilurin-resistant pathogens [19,20], was added to validate the QoI binding site of the model.

**Figure 7:**
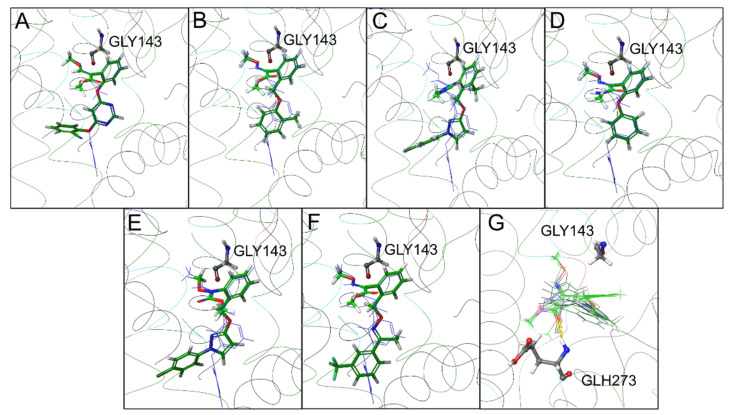
Top-scoring docking poses within the cytochrome *b* binding pocket of the commercial strobilurin fungicides included in the validation set: (**A**) azoxystrobin, (**B**) kresoxim-methyl, (**C**) metyltetraprole, (**D**) metominostrobin, (**E**) pyraclostrobin (the second top-scoring pose), and (**F**) trifloxystrobin. In (**G**), all validation set poses are reported to highlight their binding mode and the interaction with GLU273.

The top-scoring docking solution for all the validation-set compounds showed a binding mode highly similar to the azoxystrobin placement in the bovine cytochrome *bc1* (PDB ID: 1SQB) [21], in which the pharmacophore was very close to the G143 protein residue (Figure 7A–G, Table 2). The only validation compound with a top-scoring solution having a completely different orientation was pyraclostrobin; however, the second solution was similar to the co-crystallized azoxystrobin. The orientation of the methylester and the bioisosteric groups showed an interesting variability, with two different solutions (file S1). The superposition of the top-scoring poses for the entire validation set confirms a key role for Glu273 (Figure 7G) as observed in other organisms [22].

After validating the docking procedure, the five most active new compounds (**10**, **11a**, **11b**, **11e,** and **13**) were docked to gain more insights into their binding interactions (Table 3). All the five selected compounds showed a top-scoring pose with an orientation similar to the co-crystallized azoxystrobin in 1SQB and to the validation set, i.e., with the methoxyacrylate moiety close to G143; some other poses with higher energy values showed an orientation not compatible with the azoxystrobin/strobilurin binding mode.

**Figure 8:**
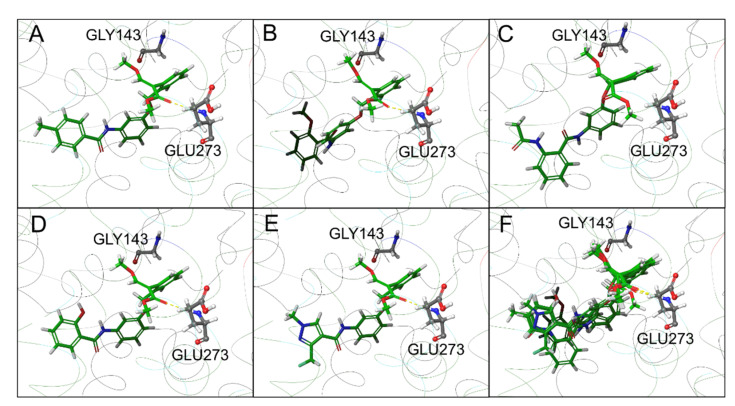
Top-scoring docking poses for compounds (**A**) **10**, (**B**) **11a**, (**C**) **11b**, (**D**) **11e**, and (**E**) **13**. In (**F**), the top-scoring docking poses for all the tested compounds are reported.

The top-scoring poses of all five compounds showed a very similar binding mode, characterized by a strong overlap of the common rings and functional groups, as reported in Figure 8.

**Figure 9:**
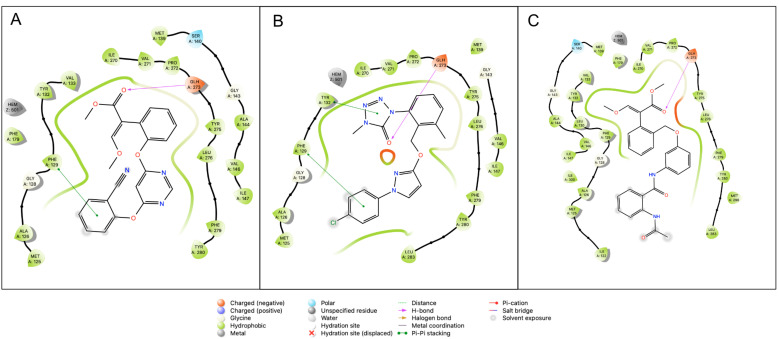
Ligand interaction diagram for (**A**) azoxystrobin, (**B**) metyltetraprole, and (**C**) compound **11b**. H-bond between the carbonyl function of the ester group and Glu273 is pointed out by the purple arrow, and π-π stacking between the distal aromatic ring and Phe129 is pointed out by green line.

Figure 9 describes the ligand interaction diagram for azoxystrobin, metyltetraprole, and compound **11b**, showing the role of Glu273 in the formation of a stable H-bond between the carbonyl function of the ester group and the amide function of the protein backbone. Moreover, in both azoxystrobin and metyltetraprole, Phe129 plays a stabilizing role through the formation of a π-π stacking interaction with the aromatic ring distal with respect to the methoxyacrylic function.

Since compound **11b** maintained its biological activity also against strobilurin-resistant strains containing the G143A mutation (Figure 3), we tested the impact of this mutation on the cytochrome *bc1* complex using a specific residue scan tool. The mutant protein showed a positive unfavorable ΔAffinity (approx. 5.7 kcal/mol), similar to the ΔAffinity value for azoxystrobin (approx. 4.7 kcal/mol) on the same mutant. On the contrary, the impact of the same mutation on the molecular recognition of metyltetraprole was negligible (approx. −0.7 kcal/mol), suggesting that the correctly oriented and less bulky methyltetrazolone ring did not negatively interact with the methyl group of A143.

## 3. Discussion

Rice blast is the main fungal disease of cultivated rice worldwide, causing 10–30% yield losses each year. Its management still relies on the use of fungicides, but the development of resistance augments food insecurity and requires urgent and innovative solutions.

In this paper, we report a structural investigation on new fungicides obtained by combining the pharmacophores of two classes of marketed antifungal compounds: strobilurins and SDH inhibitors. The study focused on the structural variations/modifications of a previously identified hit candidate, with the aim to increase the antifungal activity both on wild-type and, more importantly, on strobilurin-resistant strains of *P. oryzae*. The spatial orientation of the two pharmacophores, the role and nature of the linker, and the substitution patterns of the SDHI pharmacophore were studied to shed light on the key structural determinants of their activity.

The obtained results clearly show that the relative orientation of the QoI and SDHI pharmacophores and the entire shape of the fungicide strongly impact the biological activity of the hybrid molecule. In particular, arranging the SDHI and QoI pharmacophores in ortho position on the central ring (Figure 2, compound **7**) resulted in a significant drop in activity against the wild-type strains. Compound **8**, with the two pharmacophores in para position, maintained activity comparable to **1a** (meta position), remaining, however, almost inactive on resistant strains. This indicates that the introduction of the SDHI pharmacophore in ortho position probably creates a steric obstacle to the binding of the QoI-pharmacophore in the cytochrome *b* binding pocket, while meta and para orientations do not interfere with the binding.

The position of the substituent (methyl group) on the benzamide ring of the SDHI pharmacophore also affects the biological activity of the compounds. In particular, compound **10** with the methyl group in para on the carboxamide ring had higher activity on the resistant strains (52% inhibition) than compounds **9** (28%) and **1a** (20%).

Increasing the length of the spacer between the two pharmacophores was deleterious. In fact, compounds **14a–c**, with glycine, *β*-alanine, and γ-aminobutyric acid as linkers, and **15**, with an additional aromatic ring between the two active moieties, showed low activity. Interestingly, the activity decreased with the increase of the chain length (**14a** versus **14b** versus **14c**).

Changing the nature of the aromatic ring in the SDHI moiety by replacing the benzamide with a heterocyclic amide (compounds **12** and **13**) maintained a good activity of dual compounds on WT strains (73% and 87%, respectively). Interestingly, compound **13**, which was featured with the pyrazole carboxamide found in the commercial SDHI inhibitor fluxapyroxad, maintained some activity also on RES strains (57% inhibition).

The introduction of substituents with different steric and stereoelectronic properties on the SDHI benzamide ring had a major effect on the activity of dual compounds. Compounds **11a** and **11c** showed activity comparable to the reference scaffold **1a**, while **11d**, **11g**, and **11f** were endowed with a lower efficacy. Compounds **11b** and **11e** showed excellent inhibition of wild-type strains (>80% inhibition), with compound **11b** maintaining a strong activity also on resistant strains.

Unexpectedly, **11b** showed low activity against SDH enzyme in the succinate:quinone oxidoreductase (SQR) assay. This prompted us to further investigate the binding mode of the compound with cytochrome *bc1* by developing a three-dimensional model of *P. oryzae* cytochrome *bc1* supramolecular assembly in complex with azoxystrobin. We simulated the putative binding site of our novel compounds, evidencing that they share the same binding mode of a set of selected commercially available strobilurins. Our in silico analysis suggests that the new compounds have the same molecular recognition mechanism of azoxystrobin for the *P. oryzae* cytochrome *bc1* Qo binding site, as pointed out by their very good approx. binding free energy values computed through Glide XP Score, spanning from −12.6 to −9.1 kcal/mol, and from binding free energy values computed through MM-GBSA, spanning from −83.9 to −59.6 kcal/mol.

Interestingly, our finding suggests that the unique activity of the compound **11b** against resistant strains of *P. oryzae* cannot be easily explained by our simulations, as the G143A mutation negatively impacts the binding of the compound differently from metyltetraprole. We speculate that additional targets other than cytochrome *bc1* and SDH could be involved in the observed activity. Another intriguing hypothesis is that **11b** could undergo biotransformation in *P. oryzae*, resulting in a compound able to efficiently bypass the resistance. Future efforts will be devoted to elucidating the peculiar activity of this promising compound.

Nevertheless, the availability of a new model for *P. oryzae* cytochrome *bc1* in complex with azoxystrobin paves the way for a further rational design of new highly active compounds, possibly able to overcome the well-known strobilurin resistance in *P. oryzae*.

## 4. Materials and Methods

### 4.1. Chemistry

All reagents and solvents were reagent grade or were purified by standard methods before use. Melting points were determined in open capillaries on an SMP3 apparatus and are uncorrected.

^1^H NMR spectra were recorded on 300 and 600 MHz spectrometers; ^13^C NMR spectra were recorded on 300 and 600 MHz spectrometers. Solvents were routinely distilled prior to use; anhydrous THF and Et_2_O were obtained by distillation from sodium benzophenone ketyl; anhydrous CH_2_Cl_2_ was obtained by distillation from phosphorus pentoxide.

All reactions requiring anhydrous conditions were performed under a positive nitrogen flow, and all glassware was oven-dried. Isolation and purification of the compounds were performed by flash column chromatography on silica gel 60 (230–400 mesh). Analytical TLC was conducted on TLC plates (silica gel 60 F254, aluminum foil).

Compounds on TLC plates were detected under UV light at 254 and 365 nm or were revealed by spraying with 10% phosphomolybdic acid (PMA) in EtOH.

Compounds **16** [23], **19** [24], (E)-methyl 2-(2-(bromomethyl)phenyl)-3-methoxyacrylate [18], **22** [18], **24a** [25], **25** [26], 2-benzyloxybenzoic acid [27], and 2-*tert*-butoxycarbonylaminobenzoic acid [28] were prepared as reported in literature.

The synthetic procedures for the obtainment of all the new compounds are reported in the Supplementary Material.

### 4.2. Fungal Strains

In this study, four strains of *Pyricularia oryzae* were used; two strains belonging to the Italian population and sensitive to quinone outside inhibitor (QoI) fungicides (WT): A2.5.2 and TA102; and two strains belonging to the Japanese population and resistant to QoI (RES): PO1312 and PO1336. The strains belong to a vast collection of monoconidial isolates maintained at the laboratory of plant pathology, University of Milan [29,30]. The strains were maintained as single-spore isolates on malt-agar medium (MA: 20 g/L malt extract, Oxoid, U.K.; 15 g/L agar, Oxoid, U.K.) at 4 °C.

### 4.3. Fungicides

The commercial fungicides azoxystrobin (AZX, Amistar SC—suspension concentrate, 22.9% ai., Syngenta Crop Protection) and fluxapyroxad (FXP, Sercadis EC—emulsifiable concentrate, 30% ai., BASF Italia, S.p.A.) were used as standards to evaluate the activity of QoI (azoxystrobin) and SDHI (fluxapyroxad) fungicides.

### 4.4. Inhibition of Mycelium Growth of Pyricularia oryzae by Novel Dual Compounds

The inhibitory activity of the novel dual compounds and commercial fungicides on the mycelium growth of *P. oryzae* was evaluated as described previously [18]. In short: a mycelium plug (0.5 cm in diameter) obtained from actively growing fungal colonies of *P. oryzae* A2.5.2, TA102, PO1312, and PO1336 was transferred to MA medium plates supplemented or not with commercial fungicides (AZX, FXP) and tested compounds at the concentration of 25 mg/L in three biological replicates. Due to the low solubility of the tested dual molecules in water, they were dissolved in acetone. Therefore, two controls were included: MA medium (C, control) and MA medium supplemented with acetone at the final concentration of 1% *v*/*v* (ACT). The plates were incubated at 24 °C in the dark. The mycelium growth was measured at 7 days after inoculation (DAI), and the inhibition of mycelium growth (%) was calculated by comparing the mycelium growth on control and fungicide-supplemented plates. The inhibition percentage was calculated as I% = (C−T)/C*100, where C = mycelium growth in the control medium and T = mycelium growth in the medium added with the tested compound. For AZX and FXP, the control was MA medium. For tested compounds, the control was considered ACT.

### 4.5. Enzyme Inhibition Assay for the Measurement of SDHI and QoI Action

*P. oryzae* mitochondrial fraction from the A2.5.2 strain was prepared as described previously [18].

The SDHI action was evaluated measuring the succinate: quinone oxidoreductase (SQR) activity (i.e., the succinate dehydrogenase activity; EC 1.3.5.1) of the mitochondrial fraction in the presence of **11b** compound using a method based on the use of the redox dye, 2,6-dichlorophenolindophenol (DCPIP) [31], with some modifications as described previously [18]. The enzyme reaction was monitored at 595 nm at fixed times using a fixed wavelength microplate reader (iMark™; Bio-Rad Laboratories, Inc., Hercules, CA, USA). To calculate the SQR reaction rate, the absorbance decrease due to the DCPIP reduction during the 2–60 min time interval was considered. Fluxapyroxad (37047, Sigma Aldrich, Milan, Italy) was used as the reference SDHI.

The strobilurin-like (QoI) action was evaluated measuring the decylubiquinol: Cyt *c* reductase activity (i.e., the Cyt *bc1* complex activity; EC 1.10.2.2) of the mitochondrial fraction in the presence of the **11b** compound, using the method described by Zhu et al. [32] with some modifications [18]. Azoxystrobin (31697, Sigma Aldrich, Milan, Italy) was used as the reference QoI.

In both enzyme assays, the enzyme rate in the presence of the tested molecule (rate_molecule_) was compared to that achieved in the presence of the compound diluent DMSO (rate_DMSO_), in order to calculate the percent of inhibition (I %) as follows:

(1) $$I\;\%=\frac{{\mathrm{rate}}_{\mathrm{molecule}}-{\;\mathrm{rate}}_{\mathrm{DMSO}}}{{\mathrm{rate}}_{\mathrm{DMSO}}}\times 100$$

### 4.6. Statistical Analysis

The mycelium growth data for each treatment were grouped based on the QoI resistance of *P. oryzae* strains (WT or RES) and were submitted to ANOVA followed by a Tukey’s HSD post hoc test for multiple comparisons (*p* < 0.05) using the TukeyC package and R software, version R4.0.0 [33,34].

Similarly, ANOVA followed by posthoc Tukey’s HSD test for multiple comparisons was used for enzymatic activity data analysis.

### 4.7. In Silico Modeling

#### 4.7.1. Homology Modeling of Cytochrome bc1 Complex

The *Pyricularia oryzae* cytochrome *bc1* complex (complex III) primary structures were downloaded from the UniProt Protein Knowledgebase database (entry: Q85KP9 form *P. grisea* and G4N4E1 from *P. oryzae*, respectively [18]). After a protein BLAST search of the Protein Data Bank (RCSB PDB) database for homolog templates, the crystallographic structure of bovine cytochrome *bc1* (PDB ID: 1SQB [21]), co-crystallized with azoxystrobin, chains E and N, was selected as a template for both subunits. Two alignments produced by the ClustalΩ software and manually optimized for cytochrome *b* and 1SQB chain N were used. Comparative model building was carried out by Schrödinger BioLuminate [35] Multiple Sequence Viewer/Editor in the knowledge-based model setting, including azoxystrobin, heme groups, and Fe-S clusters. The geometry of the final model was checked by the Ramachandran plot.

#### 4.7.2. Ligand Preparation

Available/commercial ligands were downloaded from PubChem, and original ones were built with the Maestro 3D Builder tool. All ligands were prepared for docking with the LigPrep panel, using the OPLS3e [36] force field.

#### 4.7.3. Molecular Docking and Affinity Calculations

The molecular docking procedure was carried out with the Schrödinger Small-Molecule Drug Discovery Suite [37]. The QoI binding site of the cytochrome *bc1* complex was identified by the presence of azoxystrobin transferred from the bovine cytochrome *bc1* complex (PDB ID: 1SQB [21]). Molecular docking was carried out via Glide in its extra precision (XP) mode [38,39,40]. Before the docking procedures, our model of cytochrome *bc1* complex was prepared and energy-minimized via the BioLuminate Protein Preparation Wizard with the OPLS3e [36] force field. The same force field was applied in all the molecular docking procedures. The binding free energy of all the complexes produced by our molecular docking pipeline was evaluated via both Glide XP Score and Prime MM-GBSA that combines molecular mechanics with generalized Born and surface area scoring function [41]. The accuracy of the proposed methods is associated with a low level of accuracy. Glide XP Score is an empirical scoring function that approximates the ligand binding free energy, and that is generally good enough to separate ligands putatively from non-ligands. In Eberini et al. [42], we used an empirical scoring function for estimating ligand binding free energy after molecular docking: the comparison between computed and experimental affinities (i.e., dissociation constants, K_i_, computed from ΔG values) showed approx. one order of magnitude accuracy for our predictions. An approach associated with a higher level of accuracy than the one based on Glide XP Score is based on MM-GBSA that allows the ligand to relax in the binding site.

#### 4.7.4. Mutant Generation and Evaluation

The evaluation of the impact of G143A mutation on the enzyme stability and on the affinity for compound **11b** was carried out with the Maestro BioLuminate Residue Scanning Tool, which considers the impact of a mutation on the stability of the protein (ΔStability) and on the affinity (ΔAffinity) for the tested ligand(s), expressed in kcal/mol [43].

## Figures and Tables

**Table 1:** Inhibition of mycelium growth of wild-type (WT) and QoI-resistant (RES) *Pyricularia oryzae* (Scheme 1), acetone (ACT), and media supplemented with azoxystrobin (AZX), fluxapyroxad (FXP), or dual compounds (Cpds **1a**, **7–15**) at a concentration of 25 mg/L.

	WT		RES	
Compound	% Inhibition ^a^	Tukey HSD ^b^	% Inhibition	Tukey HSD
Control	0	a	0	a
ACT	0	a	−4	a
AZX	92	m	47	c
FXP	67	f h i	94	d
**1a**	72	h i j	20	b
**7**	25	b c	26	b
**8**	67	f g h i	26	b
**9**	63	e f g h	28	b
**10**	78	j k l	52	c
**11a**	79	i j k l	30	b
**11b**	83	k l	84	d
**11c**	71	h i j	35	b
**11d**	58	e f g	23	b
**11e**	84	k l m	50	c
**11f**	58	e f	23	b
**11g**	55	e	27	b
**12**	73	h i j k	22	b
**13**	87	l m	57	c
**14a**	42	d	15	b
**14b**	38	d	20	b
**14c**	18	b	15	b
**15**	31	c d	21	b

^a^ Inhibition (%) was calculated as I% = (C − T)/C*100, where C = is growth on control medium, and T = is growth on treated medium. ^b^ Tukey post hoc test of mycelium growth data. The different letters indicate statistically significant differences between mean growth (*p* > 0.05).

**Table 2:** Top-scoring docking solutions for all chemicals in the validation set. ^a^ Glide XP Score indicates the approx. binding affinity (kcal/mol) of the top-scoring binding pose. ^b^ MM-GBSA indicates a more accurate binding affinity (kcal/mol) of the top-scoring binding pose. ^c^ Number of poses for each compound obtained by the molecular docking procedure. ^d^ Range of the binding affinities (kcal/mol) for each tested chemical of the validation set. ^e^ Number of generated poses with an orientation similar to azoxystrobin (as transferred from the template) for each tested compound of the validation set.

Ligand	Glide XP ^a^ [kcal/mol]	MM-GBSA ^b^ [kcal/mol]	Number of Poses ^c^	Range ^d^ [kcal/mol]	Orientation of the Generated Poses ^e^
azoxystrobin	−10.6	−76.4	5	(−10.6; −9.4)	5/5
trifloxystrobin	−9.8	−65.6	2	(−9.8; −9.7)	2/2
kresoxym-methyl	−10.4	−68.9	1	(−10.4)	1/1
metominostrobin	−9.9	−79.3	1	(−9.9)	1/1
pyraclostrobin	−8.4	−61.7	5	(−10.0; −5.7)	3/5
metyltetraprole	−10.2	−62.9	2	(−10.2; −9.8)	2/2

**Table 3:** Top-scoring docking solutions for the selected five novel compounds showing the highest biological activity against *Pyricularia oryzae*. ^a^ Glide XP Score indicates the approx. binding affinity (kcal/mol) of the top-scoring binding poses. ^b^ MM-GBSA indicates a more accurate binding affinity (kcal/mol) of the top-scoring binding pose. ^c^ Number of poses for each compound obtained by the molecular docking procedure. ^d^ Range of the binding affinities (kcal/mol) of individual poses for each tested novel compound. ^e^ Number of generated poses with an orientation similar to azoxystrobin (as transferred from the template) for each tested novel compound.

Ligand	Glide XP ^a^ [kcal/mol]	MM-GBSA ^b^ [kcal/mol]	Number of Poses ^c^	Range ^d^ [kcal/mol]	Orientation of the Generated Poses ^e^
**10**	−11.7	−83.9	3	(−11.7; −9.1)	3/3
**11a**	−9.1	−60.3	5	(−9.1; −7.4)	1/5
**11b**	−9.4	−59.6	5	(−9.4; −7.4)	3/5
**11e**	−12.6	−83.3	5	(−12.6; −11.6)	5/5
**13**	−12.2	−78.7	2	(−12.2; −10.8)	2/2

## Supplementary Materials

The Supplementary Materials are available online at https://www.mdpi.com/article/10.3390/ijms22073731/s1.

## References

[1] FRAC FRAC Code List©* 2020: Fungal Control Agents Sorted by Cross Resistance Pattern and Mode of Action. https://cpb-us-w2.wpmucdn.com/u.osu.edu/dist/b/28945/files/2020/02/frac-code-list-2020-final.pdf.

[2] Lamberth C. (2016). Complex III inhibiting strobilurin esters, amides, and carbamates as broad-spectrum fungicides. Bioactive Carboxylic Compound Classes: Pharmaceuticals and Agrochemicals.

[3] Walter H. (2016). Fungicidal succinate-dehydrogenase-inhibiting carboxamides. Bioactive Carboxylic Compound Classes: Pharmaceuticals and Agrochemicals.

[4] Xiong L., Shen Y.-Q., Jiang L.-N., Zhu X.-L., Yang W.-C., Huang W., Yang G.-F. (2015). Succinate Dehydrogenase: An Ideal Target for Fungicide Discovery. Discovery and Synthesis of Crop Protection Products.

[5] Bartlett D.W., Clough J.M., Godwin J.R., Hall A.A., Hamer M., Parr-Dobrzanski B. (2002). The strobilurin fungicides. Pest Manag. Sci..

[6] Musso L., Fabbrini A., Dallavalle S. (2020). Natural compound-derived cytochrome bc1 complex inhibitors as antifungal agents. Molecules.

[7] Steinhauer D., Salat M., Frey R., Mosbach A., Luksch T., Balmer D., Hansen R., Widdison S., Logan G., Dietrich R.A. (2019). A dispensable paralog of succinate dehydrogenase subunit C mediates standing resistance towards a subclass of SDHI fungicides in *Zymoseptoria tritici*. PLoS Pathog..

[8] FRAC FRAC List of Plant Pathogenic Organisms Resistant to Disease Control Agents. https://www.frac.info/docs/default-source/working-groups/sdhi-fungicides/group/list-of-resistant-plant-pathogens_2012-edition.pdf?sfvrsn=ef18469a_2.

[9] Torriani S.F.F., Frey R., Buitrago C., Wullschleger J., Waldner M., Kuehn R., Scalliet G., Sierotzki H., Deising H.B., Fraaije B., Mehl A., Oerke E.C., Sierotzki H., Stammler G. (2016). Succinate-dehydrogenase inhibitor (SDHI) resistance evolution in plant pathogens. Modern Fungicides and Antifungal Compounds.

[10] Fernández-Ortuño D., Torés J.A., de Vicente A., Pérez-García A., Carisse O. (2010). The QoI fungicides, the rise and fall of a successful class of agricultural fungicides. Fungicides.

[11] FRAC FRAC Reccomendations for QoI Fungicides. https://www.frac.info/frac-teams/working-groups/qol-fungicides/recommendations-for-qoi.

[12] Asibi A.E., Chai Q., Coulter J.A. (2019). Rice blast: A disease with implications for global food security. Agronomy.

[13] Nalley L., Tsiboe F., Durand-Morat A., Shew A., Thoma G. (2016). Economic and environmental impact of rice blast pathogen (*Magnaporthe oryzae*) alleviation in the United States. PLoS ONE.

[14] Scheuermann K.K., Raimondi J.V., Marschalek R., de Andrade A., Wickert E. (2012). Magnaporthe oryzae genetic diversity and its outcomes on the search for durable resistance. The Molecular Basis of Plant Genetic Diversity.

[15] Ishii H. (2015). Rice pathogens in Japan. Fungicide Resistance in Plant Pathogens.

[16] Kunova A., Cortesi P., Wheeler M.N., Johnston B.R. (2013). Tricyclazole and azoxystrobin in rice blast management: A review of their activity and pathogen responses. Fungicides: Classification, Role in Disease Management and Toxicity Effects.

[17] Fitogest® Image Line Fitogest®. https://fitogest.imagelinenetwork.com/it/.

[18] Zuccolo M., Kunova A., Musso L., Forlani F., Pinto A., Vistoli G., Gervasoni S., Cortesi P., Dallavalle S. (2019). Dual-active antifungal agents containing strobilurin and SDHI-based pharmacophores. Sci. Rep..

[19] Suemoto H., Matsuzaki Y., Iwahashi F. (2019). Metyltetraprole, a novel putative complex III inhibitor, targets known QoI-resistant strains of *Zymoseptoria tritici* and *Pyrenophora teres*. Pest Manag. Sci..

[20] Matsuzaki Y., Yoshimoto Y., Arimori S., Kiguchi S., Harada T., Iwahashi F. (2020). Discovery of metyltetraprole: Identification of tetrazolinone pharmacophore to overcome QoI resistance. Bioorg. Med. Chem..

[21] Esser L., Quinn B., Li Y.-F., Zhang M., Elberry M., Yu L., Yu C.-A., Xia D. (2004). Crystallographic studies of quinol oxidation site inhibitors: A modified classification of inhibitors for the cytochrome bc 1 complex. J. Mol. Biol..

[22] Palsdottir H., Lojero C.G., Trumpower B.L., Hunte C. (2003). Structure of the yeast cytochrome bc1 complex with a hydroxyquinone anion Qo site inhibitor bound. J. Biol. Chem..

[23] Kim E.M., Jung C.K., Choi E.Y., Gao C., Kim S.W., Lee S.H., Kwon O.P. (2011). Highly conductive polyaniline copolymers with dual-functional hydrophilic dioxyethylene side chains. Polymer.

[24] Huang R., Li Z., Ren P., Chen W., Kuang Y., Chen J., Zhan Y., Chen H., Jiang B. (2018). N- Phenyl- N- aceto-vinylsulfonamides as efficient and chemoselective handles for N-terminal modification of peptides and proteins. Eur. J. Org. Chem..

[25] Zhou X., Wang Q., Zhao W., Xu S., Zhang W., Chen J. (2015). Palladium-catalyzed ortho-arylation of benzoic acid derivatives via C-H bond activation using an aminoacetic acid bidentate directing group. Tetrahedron Lett..

[26] Crestey F., Frederiksen K., Jensen H.S., Dekermendjian K., Larsen P.H., Bastlund J.F., Lu D., Liu H., Yang C.R., Grunnet M. (2015). Identification and electrophysiological evaluation of 2-methylbenzamide derivatives as Nav1.1 modulators. ACS Chem. Neurosci..

[27] Baramov T., Schmid B., Ryu H., Jeong J., Keijzer K., von Eckardstein L., Baik M., Süssmuth R.D. (2019). How many O-donor groups in enterobactin does it take to bind a metal cation?. Chem. Eur. J..

[28] Vilaivan T. (2006). A rate enhancement of tert-butoxycarbonylation of aromatic amines with Boc2O in alcoholic solvents. Tetrahedron Lett..

[29] Kunova A., Pizzatti C., Cortesi P. (2013). Impact of tricyclazole and azoxystrobin on growth, sporulation and secondary infection of the rice blast fungus, *Magnaporthe oryzae*. Pest Manag. Sci..

[30] Kunova A., Pizzatti C., Bonaldi M., Cortesi P. (2014). Sensitivity of nonexposed and exposed populations of *Magnaporthe oryzae* from rice to tricyclazole and azoxystrobin. Plant Dis..

[31] Ye Y.-H., Ma L., Dai Z.-C., Xiao Y., Zhang Y.-Y., Li D.-D., Wang J.-X., Zhu H.-L. (2014). Synthesis and antifungal activity of nicotinamide derivatives as succinate dehydrogenase inhibitors. J. Agric. Food Chem..

[32] Zhu X., Wang F., Li H., Yang W., Chen Q., Yang G. (2012). Design, synthesis, and bioevaluation of novel strobilurin derivatives. Chin. J. Chem..

[33] R Core Team (2020). R: A Language and Environment for Statistical Computing.

[34] Faria J.C., Jelihovschi E.G., Allaman I.B. (2020). Conventional Tukey Test.

[35] (2020). Schrödinger Release 2020-3.

[36] Harder E., Damm W., Maple J., Wu C., Reboul M., Xiang J.Y., Wang L., Lupyan D., Dahlgren M.K., Knight J.L. (2016). OPLS3: A force field providing broad coverage of drug-like small molecules and proteins. J. Chem. Theory Comput..

[37] (2020). Schrödinger Release 2020-3.

[38] Friesner R.A., Banks J.L., Murphy R.B., Halgren T.A., Klicic J.J., Mainz D.T., Repasky M.P., Knoll E.H., Shelley M., Perry J.K. (2004). Glide: A new approach for rapid, accurate docking and scoring. 1. Method and assessment of docking accuracy. J. Med. Chem..

[39] Halgren T.A., Murphy R.B., Friesner R.A., Beard H.S., Frye L.L., Pollard W.T., Banks J.L. (2004). Glide: A new approach for rapid, accurate docking and scoring. 2. Enrichment factors in database screening. J. Med. Chem..

[40] Friesner R.A., Murphy R.B., Repasky M.P., Frye L.L., Greenwood J.R., Halgren T.A., Sanschagrin P.C., Mainz D.T. (2006). Extra precision glide: Docking and scoring incorporating a model of hydrophobic enclosure for protein-ligand complexes. J. Med. Chem..

[41] Li J., Abel R., Zhu K., Cao Y., Zhao S., Friesner R.A. (2011). The VSGB 2.0 model: A next generation energy model for high resolution protein structure modeling. Proteins Struct. Funct. Bioinform..

[42] Eberini I., Rocco A.G., Mantegazza M., Gianazza E., Baroni A., Vilardo M.C., Donghi D., Galliano M., Beringhelli T. (2008). Computational and experimental approaches assess the interactions between bovine *β*-lactoglobulin and synthetic compounds of pharmacological interest. J. Mol. Graph. Model..

[43] Beard H., Cholleti A., Pearlman D., Sherman W., Loving K.A. (2013). Applying physics-based scoring to calculate free energies of binding for single amino acid mutations in protein-protein complexes. PLoS ONE.

