# Supplementary material for: Structural Investigation and Molecular Modeling Studies of Strobilurin-Based Fungicides Active against the Rice Blast Pathogen Pyricularia oryzae
Source: Int J Mol Sci. 2021 Apr 2;22(7):3731. doi: 10.3390/ijms22073731 (PMC8038316; doi:10.3390/ijms22073731)
Supplement: Supplementary file 1 [file ijms-22-03731-s001.pdf]

Supporting information for

Structural investigation and molecular modeling studies of strobilurin-based fungicides active against the rice blast pathogen *Pyricularia oryzae*

Andrea Kunova[1], Luca Palazzolo[2], Fabio Forlani[1], Giorgia Catinella[1], Loana Musso[1], Paolo Cortesi[1], Ivano Eberini[2,3,*], Andrea Pinto[1,*], Sabrina Dallavalle[1]

[1]Department of Food, Environmental and Nutritional Science (DeFENS), University of Milan, Via Celoria 2, 20133 Milan, Italy, andrea.kunova@unimi.it, fabio.forlani@unimi.it, giorgia.catinella@unimi.it, loana.musso@unimi.it, paolo.cortesi@unimi.it, andrea.pinto@unimi.it, sabrina.dallavalle@unimi.it

[2]Department of Pharmacological and Biomolecular Sciences (DiSFeB), University of Milan, Via Giuseppe Balzaretti 9, 20133 Milan, Italy, luca.palazzolo@unimi.it, ivano.eberini@unimi.it

[3]Data Science Research Center (DSRC), University of Milan, Milan, Italy, ivano.eberini@unimi.it

[*]correspondence: andrea.pinto@unimi.it, ivano.eberini@unimi.it

Table of contents

General information S1

Experimental procedures S1-S14

^{1}H-NMR and ^{13}C-NMR spectra of compounds **7**, **8**, **9**,**10**, **11a-11g**, **12**, **13**, **14a-c**, **15** S15-S48

Figure S1. Top-scoring poses of pyraclostrobin S49

References S50

General information. All reagents and solvents were reagent grade or were purified by standard methods before use. Melting points were determined in open capillaries on an SMP3 apparatus and are uncorrected. NMR spectra were recorded on Varian 300 MHz (Varian, Palo Alto, CA, USA) and Bruker AV600 (Bruker, Karlsruhe, Germany) spectrometers. Chemical shifts (δ values) and coupling constants (*J* values) are given in ppm and Hz, respectively.

Solvents were routinely distilled prior to use; anhydrous THF and Et_2O were obtained by distillation from sodium benzophenone ketyl; anhydrous CH_2Cl_2 was obtained by distillation from phosphorus pentoxide.

All reactions requiring anhydrous conditions were performed under a positive nitrogen flow, and all glassware was oven-dried. Isolation and purification of the compounds were performed by flash column chromatography on silica gel 60 (230–400 mesh). Analytical thin-layer chromatography (TLC) was conducted on TLC plates (silica gel 60 F254, aluminum foil). Compounds on TLC plates were detected under UV light at 254 and 365 nm or were revealed by spraying with 10% phosphomolybdenic acid (PMA) in ethanol.

Compounds **16**,[1] **19**,[2] (E)-methyl 2-(2-(bromomethyl)phenyl)-3-methoxyacrylate,[3] **22**,[3] **24a**,[4] **25**,[5] 2-benzyloxybenzoic acid[6] and 2-*tert*-butoxycarbonylamino-benzoic acid[7] were prepared as reported in literature.

Methyl (E)-2-(2-([2-[[tert-butoxycarbonyl]amino]phenoxy]methyl)phenyl)-3-methoxyacrylate (17). Compound **16** (200 mg, 0.96 mmol) was added at room temperature to a suspension of anhydrous K_2CO_3 (142 mg, 1.03 mmol) in acetone (4 mL) and the resulting mixture was stirred at reflux for 30 min. After cooling to room temperature, (*E*)-methyl 2-(2-

(bromomethyl)phenyl)-3-methoxyacrylate (210 mg, 0.73 mmol) and 18-crown-6 (499 mg, 2.06 mmol) were then added, and the reaction mixture was stirred at reflux for 3 h. Acetone was removed at reduced pressure and the residue was diluted with ethyl acetate (20 mL). The organic layer was washed with water (3×20 mL) and brine (20 mL), then dried with anhydrous Na_2SO_4 and concentrated under reduced pressure. The residue was purified by flash column chromatography in petroleum ether/ethyl acetate 3:1 to obtain compound **17** (72 mg, 23 %) as a white waxy solid. R_f: 0.34 in hexane/ethyl acetate 3:1. ^{1}H NMR (300 MHz, $CDCl_3$): δ 8.09 (d, J = 6.9 Hz, 1H), 7.57 (s, 1H), 7.49–7.44 (m, 1H), 7.39–7.32 (m, 2H), 7.21–7.16 (m, 1H), 7.13 (s, 1H), 6.95–6.84 (m, 2H), 6.83–6.77 (m, 1H), 4.92 (s, 2H), 3.81 (s, 3H), 3.69 (s, 3H), 1.50 (s, 9H). ^{13}C NMR (75 MHz, $CDCl_3$): δ 163.9, 159.8, 153.9, 146.8, 135.5, 132.3, 131.2, 128.4, 128.1, 127.8 (x 2C), 122.1, 121.2, 118.2, 111.5, 110.2, 80.2, 69.0, 51.3, 28.4 (× 3C).

(*E*)-Methyl 2-(2-([2-aminophenoxy]methyl)phenyl)-3-methoxyacrylate (**18**). To a solution of compound **17** (72 mg, 0.17 mmol) in dry CH_2Cl_2 (1 mL), trifluoroacetic acid (100 μL) was added dropwise at 0 °C and the reaction was stirred for 3 h at 0 °C. The solvent was evaporated. Traces of TFA were removed by addition and evaporation of toluene (2 × 1 mL). The residue was dissolved in CH_2Cl_2 and washed with a sat. solution of $NaHCO_3$. The organic phase was dried over anhydrous Na_2SO_4 and the solvent was removed in vacuo. Compound **18** (54 mg, 98%) was obtained as oil. R_f: 0.28 in hexane/ethylacetate 2:1. ^{1}H NMR (300 MHz, $CDCl_3$): δ 7.57 (s, 1H), 7.56–7.52 (m, 1H), 7.36–7.26 (m, 2H), 7.21–7.15 (m, 1H), 6.81–6.62 (m, 4H), 4.88 (s, 2H), 3.80 (s, 3H), 3.69 (s, 3H) ppm. ^{13}C NMR (75 MHz, $CDCl_3$): δ 167.9, 160.0, 146.5, 136.5, 136.2, 131.4, 131.0, 128.1, 127.8, 127.8, 121.3, 118.3, 115.1, 112.0, 110.2, 68.5, 62.0, 51.7.

Methyl (*E*)-2-(2-([4-[[tert-butoxycarbonyl]-amino]phenoxy]methyl)phenyl)-3-methoxyacrylate (20). Compound **19** (209 mg, 1.01 mmol) [reference] was added at room temperature to a suspension of anhydrous K_2CO_3 (151 mg, 1.09 mmol) in acetone (4 mL), and the resulting mixture was stirred at reflux for 30 min. After cooling to room temperature, (*E*)-methyl 2-(2-(bromomethyl)phenyl)-3-methoxyacrylate (222 mg, 0.78 mmol) and 18-crown-6 (529 mg, 2.18 mmol) were added and the reaction mixture was stirred at reflux for 3 h. Acetone was removed at reduced pressure and the residue was diluted with ethyl acetate (20 mL). The organic layer was washed with water (3 × 20 mL) and brine (20 mL). The organic layer was dried over anhydrous Na_2SO_4 and concentrated under reduced pressure. The residue was purified by flash chromatography in petroleum ether/ethyl acetate 3:1 to obtain compound **20** (207 mg, 64%) as a white sticky solid. R_f: 0.18 in hexane/ethyl acetate 3:1. ^{1}H NMR (300 MHz, $CDCl_3$): δ 7.57 (s, 1H), 7.54–7.49 (m, 1H), 7.36–7.27 (m, 2H), 7.21 (d, J = 8.9 Hz, 2H), 7.18–7.14 (m, 1H), 6.88–6.79 (m, 2H), 6.34 (s, 1H), 4.99 (s, 2H), 3.81 (s, 3H), 3.70 (s, 3H), 1.51 (s, 9H). ^{13}C NMR (75 MHz, $CDCl_3$: δ 167.8, 160.0, 155.0, 153.1, 136.1, 131.5, 131.2, 130.9, 128.4, 128.0, 127.4 (x 2C), 120.6, 115.2 (× 2C), 110.2, 80.2, 68.4, 61.9, 51.6, 28.3 (× 3C).

(*E*)-Methyl 2-(2-([4-aminophenoxy]methyl)phenyl)-3-methoxyacrylate (21). To a solution of compound **20** (109 mg, 0.26 mmol) in dry CH_2Cl_2 (1.5 mL), trifluoroacetic acid (150 µL) was added dropwise at 0 °C and the reaction was stirred 3 h at 0 °C. The solvent was evaporated and the crude was treated with toluene (2 × 1 mL) to remove TFA. The residue was dissolved in CH_2Cl_2 and washed with a sat. solution of $NaHCO_3$. The organic phase was dried with anhydrous Na_2SO_4 and the solvent was removed in vacuo. Compound **21** (78 mg, 95%) was obtained as an oil. R_f: 0.11 in hexane/ethylacetate 2:1. ^{1}H NMR (300 MHz, $CDCl_3$): δ 7.60 (s, 1H), 7.56–7.52 (m, 1H),

7.36–7.26 (m, 2H), 7.18–7.13 (m, 1H), 6.77–6.71 (m, 2H), 6.63–6.57 (m, 2H), 4.99 (s, 2H), 3.84 (s, 3H), 3.72 (s, 3H) ppm. ^{13}C NMR (75 MHz, $CDCl_3$): δ 167.9, 160.0, 146.5, 136.5, 136.2, 131.4, 131.0, 128.1, 127.8, 127.6, 121.3, 118.3, 115.1, 112.0, 110.2, 68.5, 61.9, 51.6 ppm.

General procedure for the synthesis of compounds 7 and 8. To a solution of 2-methylbenzoic acid (0.26 mmol) in CH_2Cl_2 (1.5 mL) at 0 °C under nitrogen atmosphere, EDC·HCl (0.33 mmol) and HOBt (0.33 mmol) were added. The reaction mixture was stirred for 30 min at 0 °C, then a solution of aniline (compound **18** or **21**, 0.22 mmol) in CH_2Cl_2 (1 mL) and DIPEA (0.44mmol) was added dropwise at 0 °C. The reaction was stirred for 24 h at room temperature, then the mixture was diluted with ethyl acetate and washed with sat. NH_4Cl, sat. $NaHCO_3$, and brine. The organic phase was dried over anhydrous Na_2SO_4, and the solvent was removed by evaporation. The residue was purified by flash chromatography to give the desired compound.

Methyl (*E*)-3-methoxy-2-(2-(2-[2-methylbenzamido]-phenoxy)-methyl)-phenyl)-acrylate (7). Obtained according to the above procedure from 2-methylbenzoic acid and aniline **18**. Purified by flash chromatography in hexane:ethyl acetate 3:1 (26 mg, 35 %). Yellow sticky solid. R_f: 0.21 in hexane/ethyl acetate 2:1. ^{1}H NMR (300 MHz, $CDCl_3$): δ 8.56–8.48 (m, 1H), 8.22 (s, 1H), 7.52 (s, 1H), 7.49–7.39 (m, 2H), 7.37–7.27 (m, 3H), 7.25–7.14 (m, 3H), 7.05–6.96 (m, 2H), 6.90–6.83 (m, 1H), 5.04 (s, 2H), 3.70 (s, 3H), 3.61 (s, 3H), 2.49 (s, 3H). ^{13}C NMR (75 MHz, $CDCl_3$): δ 167.8, 167.7, 160.1, 147.5, 136.6, 135.5, 131.4 (x 2C), 131.3, 130.2, 128.2, 127.9, 127.5, 127.2, 125.9, 124.8, 123.9, 121.3, 120.0, 111.9, 109.9, 69.2, 61.9, 51.7, 20.0.

3-Methoxy-2-(2-(4-[2-methyl-benzoylamino]phenoxymethyl)phenyl)acrylic acid methyl ester (8). Obtained according to general procedure from 2-methylbenzoic acid and amine **21**. Purified by flash chromatography in hexane:ethyl acetate 55:45 (61 mg, 64 %). White solid. R_f: 0.60 in hexane/ethyl acetate 1:1. Mp: 182 °C. ^{1}H NMR (300 MHz, $CDCl_3$): δ 7.58 (s, 1H), 7.56–7.42 (m, 4H), 7.40–7.30 (m, 4H), 7.29–1.22 (m, 2H); 7.20–7.15 (m, 2H), 6.95–6.85 (m, 2H), 4.96 (s, 2H), 3.83 (s, 3H), 3.71 (s, 3H), 2.49 (s, 3H). ^{13}C NMR (75 MHz, $CDCl_3$): δ 167.8 (x 2C), 159.9, 155.9, 136.6, 136.0, 131.3, 131.2, 131.1, 131.0, 130.1, 128.0, 127.5, 127.4, 126.6, 125.8, 121.7, 115.3, 110.2, 68.4, 61.9, 51.6, 19.7.

General procedure for the synthesis of compounds 9, 10, 11a, 11b, and 12.

To a solution of suitable benzoic acid (0.18 mmol) in CH_2Cl_2 (1 mL) at 0 °C under nitrogen atmosphere, EDC·HCl (0.19 mmol) and HOBt (0.19 mmol) were added. The reaction mixture was stirred at 0 °C for 1 h. After that, a solution of compound **22** (0.16 mmol) in CH_2Cl_2 (0.7 mL) and DIPEA (0.32 mmol, 56 µL) was added dropwise at 0 °C. The reaction mixture was stirred at room temperature for 8 h. Further amounts of benzoic acid (0.18 mmol), EDC·HCl (0.19 mmol), HOBt (0.19 mmol), and DIPEA (0.32 mmol, 56 µL) were added at 0 °C after 24 and 48 h, and then the reaction was stirred for a further 24 h. The mixture was diluted with ethyl acetate (15 mL) and washed with a sat. NH_4Cl (3×20 mL), sat. $NaHCO_3$ (20 mL) solution, and brine (20 mL). The organic layer was dried over anhydrous Na_2SO_4, and the solvent was removed at reduced pressure. The residue was purified by flash chromatography to give the desired compounds.

Methyl (*E*)-3-methoxy-2-(2-([3-[3-methylbenzamido]phenoxy]methyl)phenyl)acrylate (9).

Obtained according to the above procedure from compound **22** and 3-methylbenzoic acid. The crude was purified by flash chromatography in hexane/ethyl acetate 3:1 to give compound **9** (58.3 mg, 84 %) as a white waxy solid. ^{1}H NMR (600 MHz, $CDCl_3$): *δ* 7.92 (s, 1H), 7.70 (s, 1H), 7.67 (d, *J* = 6.3 Hz, 1H), 7.58 (s, 1H), 7.55 (d, *J* = 7.3 Hz, 1H), 7.28–7.36 (m, 4H), 7.25 (s, 1H), 7.20 (dd, *J* = 8.3, 8.3 Hz, 1H), 7.16 (d, *J* = 7.3 Hz, 1H), 7.11 (s, 1H), 6.68 (dd, *J* = 1.4, 8.2 Hz, 1H), 4.98 (s, 2H), 3.82 (s, 3H), 3.67 (s, 3H), 2.41 (s, 3H). ^{13}C NMR (150 MHz, $CDCl_3$): *δ* 168.1, 165.8, 160.4, 159.2, 139.3, 138.6, 136.2, 135.0, 132.5, 131.1, 130.9, 129.7, 128.5, 128.2, 127.9, 127.6, 127.5, 124.0, 112.4, 111.5, 109.9, 106.3, 67.9, 62.1, 51.7, 21.4.

Methyl (*E*)-3-methoxy-2-(2-([3-[4-methylbenzamido]phenoxy]methyl)phenyl)acrylate (10). Obtained according to general procedure from **22** and 4-methylbenzoic acid. The crude was purified by flash chromatography in hexane/ethyl acetate 3:1 to give compound **10** (56.5 mg, 82 %) as a white waxy solid. ^{1}H NMR (600 MHz, $CDCl_3$): *δ* 7.93 (s, 1H), 7.81–7.77 (m, 2H), 7.59 (s, 1H), 7.55 (dd, *J* = 1.3, 7.6 Hz, 1H), 7.36–7.28 (m, 3H), 7.27-7.23 (m, 2H), 7.20 (dd, *J* = 8.1, 8.2 Hz, 1H), 7.15 (dd, *J* = 1.3, 7.1, 1H), 7.07–7.09 (m, 1H), 6.68 (ddd, *J* = 0.8, 2.4, 8.2 Hz, 1H), 4.98 (s, 2H), 3.82 (s, 3H), 3.68 (s, 3H), 2.40 (s, 3H). ^{13}C NMR (150 MHz, $CDCl_3$): *δ* 168.1, 165.5, 160.4, 159.2, 142.2, 139.3, 136.2, 132.1, 131.1, 130.9, 129.7, 129.3, 128.9, 128.2, 127.5 (×2), 127.1, 120.3, 112.4, 111.4, 109.9, 106.2, 67.8, 62.1, 51.8, 21.5.

Methyl (*E*)-3-methoxy-2-(2-([3-[2-methoxybenzamido]phenoxy]methyl)phenyl)acrylate (11a). Obtained according to the general procedure from **22** and 2-metoxybenzoic acid and purified by flash chromatography in hexane:ethyl acetate 3:1 (81.0 mg, 64%). White waxy solid. ^{1}H NMR (600 MHz, $CDCl_3$): δ = 9.77 (s, 1H), 8.27 (dd, *J* = 7.8, 19 Hz, 1H), 7.59 (s, 1H), 7.58–

7.47 (m, 2H), 7.40–7.32 (m, 3H), 7.22–7.11 (m, 4H), 7.03 (d, J = 8.3 Hz, 1H), 6.77–6.59 (m, 1H), 4.99 (s, 2H), 4.04 (s, 3H), 3.83 (s, 3H), 3.70 (s, 3H). ^{13}C NMR (75 MHz, $CDCl_3$): δ 168.9, 163.1, 160.1, 159.4, 157.2, 139.5, 136.0, 133.2, 132.5, 131.3, 131.0, 129.6, 128.1, 127.7, 127.5, 121.9, 121.7, 112.9, 111.5, 110.5, 110.0, 107.3, 68.1, 62.0, 56.2, 51.7.

Methyl (*E*)-3-methoxy-2-(2-([3-[2-acetamidobenzamido]phenoxy]methyl)phenyl)acrylate (11b). Obtained according to the general procedure from compound **22** and 2-acetamidobenzoic acid and purified by flash chromatography in hexane:ethyl acetate 1:1 (50 mg, 59%) to give a white waxy solid. ^{1}H NMR (300 MHz, $CDCl_3$): δ 8.26 (d, J = 7.9 Hz, 1H), 7.79–7.72 (m, 1H), 7.66 (d, J = 8.0 Hz, 1H), 7.56 (s, 1H), 7.54–7.37 (m, 3H), 7.36–7.27 (m, 2H), 7.18–7.13 (m, 1H), 7.06 (dd, J = 8.4, 1.7 Hz, 1H), 6.81 (d, J = 7.8 Hz, 1H), 6.71 (t, J = 2.1 Hz, 1H), 5.02 (m, 2H), 3.74 (s, 3H), 3.61 (s, 3H), 2.11 (s, 3H). ^{13}C NMR (75 MHz, $CDCl_3$): δ 167.8, 162.3, 160.3, 160.0, 154.6, 147.6, 138.7, 135.6 (×2 C), 134.7, 131.3, 130.7, 128.2, 127.7, 127.3, 127.1, 126.9, 126.7, 120.9, 120.2, 116.5, 114.6, 110.0, 68.2, 62.1, 51.8, 24.1.

Methyl (*E*)-3-methoxy-2-(2-([3-[3-methylthiophene-2-carboxamido]-phenoxy]-methyl)-phenyl)-acrylate (12). Prepared according to the general procedure from **22** and 2-methylthiophene-3-carboxylic acid. Purified by flash chromatography in hexane:ethyl acetate 3:1 (79 mg, 62%). White waxy solid. ^{1}H NMR (600 MHz, CDCl3): δ 7.59 (s, 1H), 7.57–7.51 (m, 2H), 7.36–7.29 (m, 3H), 7.22–7.14 (m, 4H), 6.93 (d, J = 5.1 Hz, 1H), 6.68 (dd, J = 1.8, 7.8 Hz, 1H), 4.98 (s, 2H), 3.83 (s, 3H), 3.69 (s, 3H), 2.57 (s, 3H). ^{13}C NMR (150 MHz, $CDCl_3$): δ 168.2, 161.4, 160.4, 159.5, 142.4, 139.0, 136.1, 132.4, 131.3, 131.1, 130.8, 129.7, 128.1, 127.6 (×2C), 127.1, 112.8, 111.5, 110.1, 106.9, 68.0, 62.0, 51.7, 15.8.

General procedure for the synthesis of compounds 11c,d,f, 14a-c, 15. To a solution of a suitable acid (0.26 mmol) in CH_2Cl_2 (1.5 mL) at 0 °C under nitrogen atmosphere, EDC·HCl (0.33 mmol) and HOBt (0.33 mmol) were added. The reaction mixture was stirred at 0 °C for 30 min. After that, a solution of amine **22** (0.22 mmol) in abs. CH_2Cl_2 (1 mL) was added dropwise at 0 °C. Then, DIPEA (0.44mmol) was added dropwise at 0 °C, and the reaction mixture was stirred at room temperature for 24 h. The reaction mixture was diluted with ethyl acetate and washed with 1M HCl, a saturated solution of $NaHCO_3$, and brine. The organic phase was dried over anhydrous Na_2SO_4, and the solvent was removed in vacuo. The residue was purified by flash chromatography to give the desired compounds.

Methyl (*E*)-3-iodo-2-(2-([3-[2-methoxybenzamido]phenoxy]methyl)phenyl)acrylate (11c). Prepared according to general procedure from **22** and 2-iodobenzoic acid. Purified by flash chromatography in hexane:ethyl acetate 2:1 (87 mg, 86%). White solid. R_f: 0.46 hexane:ethyl acetate 1:1. ^{1}H NMR (300 MHz, $CDCl_3$): δ 7.93-7.87 (m, 1H), 7.59 (s, 1H), 7.58–7.49 (m, 3H), 7.42 (t, J = 7.5 Hz, 1H), 7.35–7.30 (m, 3H), 7.24–7.10 (m, 4H), 6.74–6.69 (m, 1H), 4.98 (s, 2H), 3.83 (s, 3H), 3.66 (s, 3H). ^{13}C NMR (75 MHz, $CDCl_3$): δ 167.9, 167.0, 160.1, 159.4, 142.2, 140.0, 138.7, 136.0, 131.4, 131.3, 130.9, 129.8, 128.5, 128.3, 128.1, 127.6, 127.5, 112.4, 111.6, 110.1, 106.7, 92.3, 68.1, 61.9, 51.6.

2-(2-(3-[2-Benzyloxybenzoylamino]phenoxymethyl)phenyl)-3-methoxy-acrylic acid methyl ester (11d). Prepared according to general procedure from **22** and 2-benzyloxybenzoic acid. Purified by flash chromatography in hexane:ethyl acetate 6:4 (59 mg, 71%). Brown oil. R_f: 0.13

hexane:ethyl acetate 7:3. ^{1}H-NMR- (600 MHz, $CDCl_3$): δ 9.95 (1H, s); 8.33 (1H, dd, J = 1.4 Hz, 7.8 H, 1H); 7.58 (s, 1H); 7.57–7.49 (m, 4H); 7.49–7.42 (m, 3H); 7.39–7.30 (m, 2H); 7.18 (d, J = 7.8 Hz, 1H); 7.16–7.12 (m, 1H); 7.06–7.02 (m, 1H); 6.66 (dd, J = 1.6 Hz, 7.8 Hz, 1H); 6.56 (dd, J = 2.5 Hz; 8.3 Hz, 1H); 5.24 (s, 2H); 4.89 (s, 2H); 3.83 (s, 3H); 3.70 (s, 3H). ^{13}C-NMR- (150 MHz, $CDCl_3$): δ 167.9, 162.9, 160.1, 159.3, 156.6, 139.6, 136.0, 135.2, 133.2, 132.6, 131.4, 130.9, 129.3, 129.2 (× 4C), 128.6 (× 2C), 128.0, 127.8, 127.5, 121.9, 112.6, 112.2, 110.3, 110.0, 106.7, 71.8, 68.1, 62.0, 51.7.

2-(2-(3-[2-Hydroxy-benzoylamino]-phenoxymethyl)-phenyl)-3-methoxy-acrylic acid methyl ester (11e). To a solution of compound **11d** (40 mg, 0.076 mmol) in ethyl acetate (0.6 mL), 10% Pd/C (8 mg) was added. The suspension was evaporated under vacuum and flushed with H_2 gas (× 3). The reaction mixture was stirred overnight under H_2 at room temperature, then it was filtered through a plug of celite and the residue purified by flash chromatography in hexane:ethyl acetate 6:4 to give compound **11e** in 91% yield. Pale oil. R_f: 0.44 in hexane:ethyl acetate 6:4. ^{1}H-NMR (600 MHz, $CDCl_3$): δ 12.15 (bs, 1H), 8.24 (s, 1H), 7.73 (dd, J = 1.2 Hz, 8.1 Hz, 1H), 7.63 (s, 1H); 7.57 (dd, J = 1.9 Hz, 7.6 Hz, 1H), 7.45–7.41 (m, 1H), 7.40 (dd, J = 1.6 Hz, 7.8 Hz, 1H), 7.38–7.31 (m, 2H), 7.28–7.22 (m, 1H), 7.19 (dd, J = 1.9 Hz, 7.3 Hz, 1H), 7.01 (d, J = 8.1 Hz, 1H), 6.94 (dd, J = 2.2 Hz, 2.2 Hz, 1H), 6.90 (dd, J = 7.8 Hz, 7.8 Hz, 1H), 6.77 (dd, J = 2.4 Hz, 8.2 Hz, 1H), 5.02 (s, 2H); 3.86 (s, 3H); 3.72 (s, 3H). ^{13}C-NMR (150 MHz, $CDCl_3$): δ 168.5, 168.4, 161.9, 160.6, 159.0, 138.1, 136.2, 134.5, 131.1, 130.9, 130.8, 129.8, 128.4, 127.8, 127.6, 127.3, 125.9, 118.7 (× 2C), 114.6, 113.1, 112.5, 109.8, 106.8, 67.7, 62.1, 51.9.

2-(2-(3-[2-tert-Butoxycarbonylamino-benzoylamino]phenoxymethyl)phenyl)-3-methoxy-acrylic acid methyl ester 11f. Prepared according to general procedure from **22** and 2-*tert*-butoxycarbonylamino-benzoic acid. Purified by flash chromatography in hexane:ethyl acetate 8:2→6:4 (28 mg, 41%). Pale oil. R_f: 0.48 hexane:ethyl acetate 4:6. ^{1}H-NMR (600 MHz, $CDCl_3$): δ 9.88 (s, 1H), 8.34 (d, J = 8.2 Hz, 1H), 8.09 (s, 1H), 7.62 (d, J = 7.9 Hz, 1H); 7.60 (s, 1H), 7.57 (d, J = 7.3 Hz, 1H), 7.46–7.42 (m, 1H), 7.37–7.30 (m, 2H), 7.30–7.22 (m, 2H), 7.17 (d, J = 7.6 Hz, 1H), 7.05–7.00 (m, 2H), 6.37 (d, J = 7.9 Hz, 1H), 4.99 (s, 2H), 3.84 (s, 3H), 3.66 (s, 3H), 1.51 (s, 9H).^{13}C-NMR (150 MHz, $CDCl_3$): δ 168.2, 167.3, 160.4, 159.2, 153.1, 140.2, 138.6, 136.1, 132.6, 131.0, 130.9, 129.8, 128.3, 127.6, 127.4, 126.9, 121.5, 120.5, 120.1, 112.9, 111.9, 109.9, 106.9, 80.4, 67.8, 62.0, 51.8, 28.3 (× 3C).

2-(2-(3-[2-Aminobenzoylamino]phenoxymethyl)phenyl)-3-methoxy-acrylic acid methyl ester (11g). To a stirred solution of compound **11f** (28 mg, 0.05 mmol) in dry CH_2Cl_2 (0.3 mL), TFA (30 μL) was added at 0 °C. The reaction mixture was warmed to room temperature and stirred for 2 h. The reaction mixture was concentrated in vacuo. The residue was diluted with ethyl acetate and washed with a saturated solution of $NaHCO_3$ and brine. The organic phase was dried over anhydrous Na_2SO_4 and the solvent was removed in vacuo. The residue was purified by flash chromatography in hexane:ethyl acetate 6:4 to afford compound **11g** (12 mg, 52% yield). Yellow oil. R_f: 0.24 in hexane:ethyl acetate 1:1. ^{1}H-NMR- (600 MHz, $CDCl_3$): δ 8.07 (s, 1H), 7.60 (s, 1H), 7.59–7.54 (m, 2H), 7.37–7.29 (m, 2H), 7.29–7.24 (m, 2H), 7.23–7.15 (m, 2H), 7.07 (m, 1H), 6.84 (d, J = 8.3 Hz, 1H), 7.82–7.77 (m, 1H), 6.69 (dd, J = 1.8 Hz, 8.3 Hz, 1H), 4.98 (s, 2H), 3.83 (s, 3H), 3.68 (s, 3H). ^{13}C-NMR-NMR (150 MHz, $CDCl_3$): δ 168.2, 167.2, 160.4, 159.1, 146.2, 139.0,

136.1, 132.7, 131.1 (×2C), 130.9, 129.7, 128.2, 127.6, 127.5, 118.7, 118.6, 117.8, 112.9, 111.5, 109.9, 106.8, 67.8, 62.1, 51.8.

(E)-Methyl 2-(2-([3-[3-[difluoromethyl]-1-methyl-1H-pyrazole-4-carboxamido]phenoxy]methyl)phenyl)-3-methoxyacrylate (13). Prepared according to the general procedure from **22** and 3-(difluoromethyl)-1-methyl-1H-pyrazole-4-carboxylic acid. Purified by flash chromatography in hexane:ethyl acetate 45:55 (93 mg, 57%). White solid. mp: 139–141 °C. R_f: 0.07 hexane:ethyl acetate 1:1. ^{1}H NMR (300 MHz, $CDCl_3$): δ 8.07 (m, 1H), 8.04 (s, 1H), 7.59 (s, 1H), 7.57–7.53 (m, 1H), 7.38–7.28 (m, 2H), 7.24–7.14 (m, 3H), 713–7.10 (m, 1H); 6.97 (t, J = 54.4 Hz, 1H), 6.72–6.63 (m, 1H), 4.97 (s, 2H), 3.94 (s, 3H), 3.83 (s, 3H), 3.70 (s, 3H). ^{13}C NMR (75 MHz, $CDCl_3$): δ 168.2 (x 2C), 160.5, 159.3, 143.5, 138.9, 136.1, 131.2, 130.9, 129.8, 128.2, 127.7, 127.6 (x 2C), 117.2, 114.5, 112.4, 111.2, 109.9, 106.6, 68.0, 62.0, 51.8, 39.6.

3-(2-Methylbenzamido)propanoic acid (24b). To a solution of β-alanine (688 mg, 7.72 mmol) in water (8 mL), pH was adjusted to 10 with 2M NaOH, then 2-methylbenzoyl chloride (1.43 g, 9.26 mmol) was added, and the reaction was stirred at rt. The reaction was acidified to pH 2 by adding conc. HCl, and the resulting mixture was extracted with ethyl acetate. The organic phase was dried over anhydrous Na_2SO_4, and the solvent was evaporated. The crude was purified by flash chromatography in hexane:ethyl acetate 1:1 to hexane:ethyl acetate 1:1 + 1% CH_3COOH to give 384 mg (24%) of the title compound. White solid; m.p.: 103–105 °C. R_f: 0.14 in hexane:ethyl acetate 1:1. ^{1}H NMR (300 MHz, $CDCl_3$): δ 7.36–7.27 (m, 2H); 7.23–7.14 (m, 2H); 6.40 (t, J = 5.1 Hz, 1H); 3.74–3.65 (m, 2H); 2.70 (t, J = 6.1 Hz, 2H); 2.41 (s, 3H).

3-(2-Methylbenzamido)-butanoic acid (24c). To a solution of GABA (747 mg, 7.24 mmol) in water (3 mL), 2M NaOH was added (10 mL), and the solution was cooled at 0°C. Then, 2-methylbenzoyl chloride (1.34g, 8.69 mmol) was added dropwise, and the reaction was stirred for 2 h at 0 °C and overnight at rt. The reaction was acidified to pH 2 by adding conc. HCl, and the resulting mixture was extracted in ethyl acetate. The organic phase was dried over anhydrous Na_2SO_4, and the solvent was evaporated. The resulting crude was purified by flash chromatography from hexane:ethyl acetate 1:1 to hexane:ethyl acetate 1:1 + 1% CH_3COOH to give 657 mg (41%) of the title compound. White solid; m.p.: 89 °C. R_f: 0.12 in hexane:ethyl acetate 1:1. ^{1}H NMR (300 MHz, $CDCl_3$): δ 7.37–7.27 (m, 2H), 7.24–7.15 (m, 2H), 6.06 (t, J = 6.4 Hz, 1H), 3.55–3.43 (m, 2H), 2.47 (t, J = 7.1 Hz, 2H), 2.43 (3H, s), 2.00–1.88 (2H, m).

3-Methoxy-2-(2-(3-[2-[2-methylbenzoylamino]-acetylamino]-phenoxymethyl)-phenyl)-acrylic acid methyl ester (14a). Prepared according to the general procedure from **23** and **24a**. Purified by flash chromatography in hexane:ethyl acetate 45:55 (18 mg, 18%). Light yellow solid; m.p.: 170 °C. R_f: 0.21 in hexane:ethyl acetate 45:55. ^{1}H NMR (300 MHz, $CDCl_3$): δ 8.50 (s, 1H), 7.60 (s, 1H), 7.57–7.40 (m, 2H), 7.40–7.29 (m, 3H), 7.28–7.07 (m, 6), 6.75 (s, 1H), 6.67 (s, 1H), 4.95 (s, 2H), 4.29 (d, J = 4.7 Hz, 2H), 3.84 (s, 3H), 3.74 (s, 3H), 2.48 (s, 3H). ^{13}C NMR (75 MHz, $CDCl_3$): δ 170.8, 168.1, 166.9, 160.4, 159.4, 138.9, 136.5, 136.2, 135.2, 131.2,, 131.1, 130.5, 129.8, 128.3, 127.8 (× 2C), 127.2, 126.0, 112.3, 111.1, 110.1, 106.7, 68.1, 62.1, 51.9, 44.8, 29.7, 19.9.

3-Methoxy-2-(2-(3-[3-[2-methylbenzoylamino]-propionylamino]-phenoxymethyl)-phenyl)-acrylic acid methyl ester (14b). Prepared according to general procedure from **23** and **24b**. Purified by flash chromatography in hexane:ethyl acetate 1:10 (53 mg, 49%). Green solid; m.p.:

140 °C. R_f: 0.2 in hexane:ethyl acetate 1:1. ^{1}H NMR (300 MHz, $CDCl_3$): δ 7.88 (s, 1H), 7.59 (s, 1H), 7.56–7.49 (m, 1H), 7.36–7.27 (m, 4H), 7.22–7.11 (m, 5H), 6.98 (s, 1H), 6.69–6.60 (m, 2H), 4.94 (s, 2H), 3.82 (s, 3H), 3.75 (dd, J = 10.3, 4.4 Hz, 2H), 3.72 (s, 3H), 2.70 (t, J =5.8, 2H), 2.41 (s, 3H). ^{13}C NMR (75 MHz, $CDCl_3$): δ 170.5, 169.9, 168.1, 160.3, 159.2, 139.1, 136.1 (x 2C), 135.9, 131.2, 131.0, 130.9, 129.9, 129.6, 128.1, 127.5 (×2C), 126.8, 125.8, 112.1, 110.9, 109.9, 106.4, 67.9, 62.0, 51.7, 36.6, 35.7, 19.8.

3-Methoxy-2-(2-(3-[4-[2-methyl-benzoylamino]butyrylamino]phenoxymethyl)phenyl)acrylic acid methyl ester (14c). Prepared according to general procedure from **23** and **24c**. Purified by flash chromatography in hexane:ethyl acetate 1:10 (44 mg, 40%). Light green solid; m.p.: 136 °C. R_f: 0.2 in hexane:ethyl acetate 1:1. ^{1}H NMR (300 MHz, $CDCl_3$): δ 8.79 (s, 1H), 7.58 (s, 1H), 7.57–7.50 (m, 1H), 7.38–7.27 (4H, m), 7.25–7.11 (6H, m), 6.63 (d, J = 7.6 Hz, 1H), 6.21 (1H, t, J = 5.5 Hz), 4.94 (s, 2H), 3.81 (s, 3H), 3.71 (s, 3H), 3.58–3.49 (m, 2H), 2.50–2.41 (m, 5H), 2.05–1.94 (m, 2H).^{13}C NMR (75 MHz, $CDCl_3$): δ 171.1, 168.1, 160.2, 159.2, 139.5, 136.1, 136.0, 135.9, 131.2, 131.1 (× 2C), 130.9, 130.0, 129.5, 128.1, 127.7, 127.5, 126.7, 125.8, 112.1, 110.6, 109.9, 106.3, 67.9, 61.9, 51.7, 39.1, 34.9, 26.3, 19.8.

3-Methoxy-2-(2-(3-[3-[2-methylbenzoylamino]benzoylamino]phenoxymethyl)phenyl)acrylic acid methyl ester (15). Prepared according to the general procedure from **22** and 3-(2-methylbenzoylamino)benzoic acid (**25**). Purified by flash chromatography in hexane:ethyl acetate 2:1 (40 mg, 33%). Light green solid; m.p.: 129–131 °C. R_f: 0.36 in hexane:ethyl acetate 1:1. ^{1}H NMR (300 MHz, $CDCl_3$): δ 8.13 (s, 1H), 8.05 (s, 1H), 7.93 (d, J = 7.5 Hz, 1H), 7.76–7.65 (m, 2H),

7.59 (s, 1H), 7.60–7.08 (m, 12H), 6.70 (dd, J = 8.2, 1.7 Hz, 1H), 4.98 (s, 2H), 3.83 (s, 3H), 3.67 (s, 3H), 2.51 (s, 3H). ^{13}C NMR (75 MHz, $CDCl_3$): δ 168.2, 168.1, 165.1, 160.3, 159.1, 139.1, 138.5, 136.5, 136.1, 135.9, 135.6, 131.3, 131.1, 130.8, 130.4, 129.7, 129.4, 128.2, 127.5, 127.4, 126.6, 125.9, 123.1, 122.8, 118.6, 112.6, 111.5, 109.9, 67.9, 62.0, 51.7, 19.8.

^{1}H-NMR (300 MHz, $CDCl_3$) compound **7**.

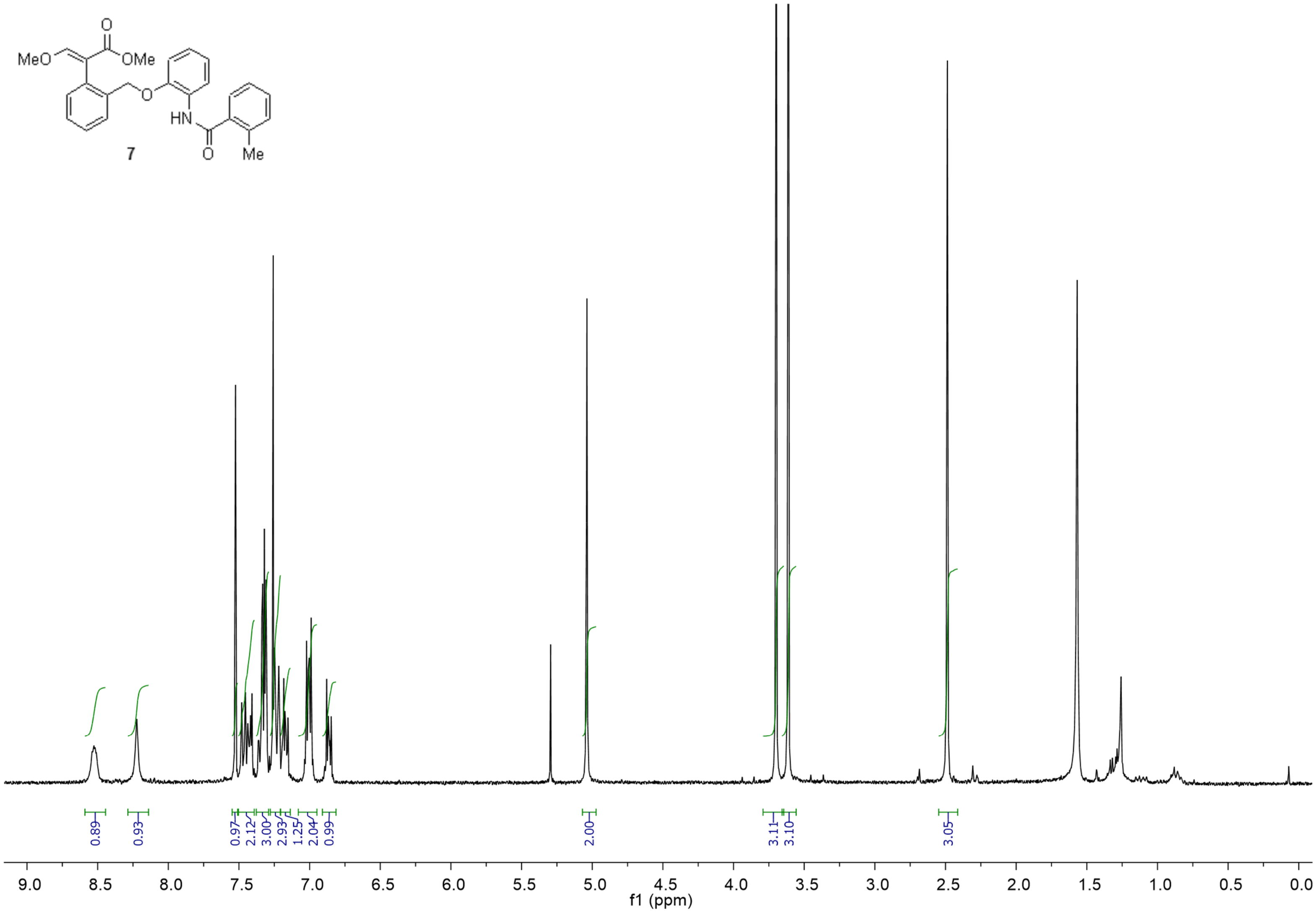

^{13}C-NMR (75 MHz, $CDCl_3$) compound **7.**

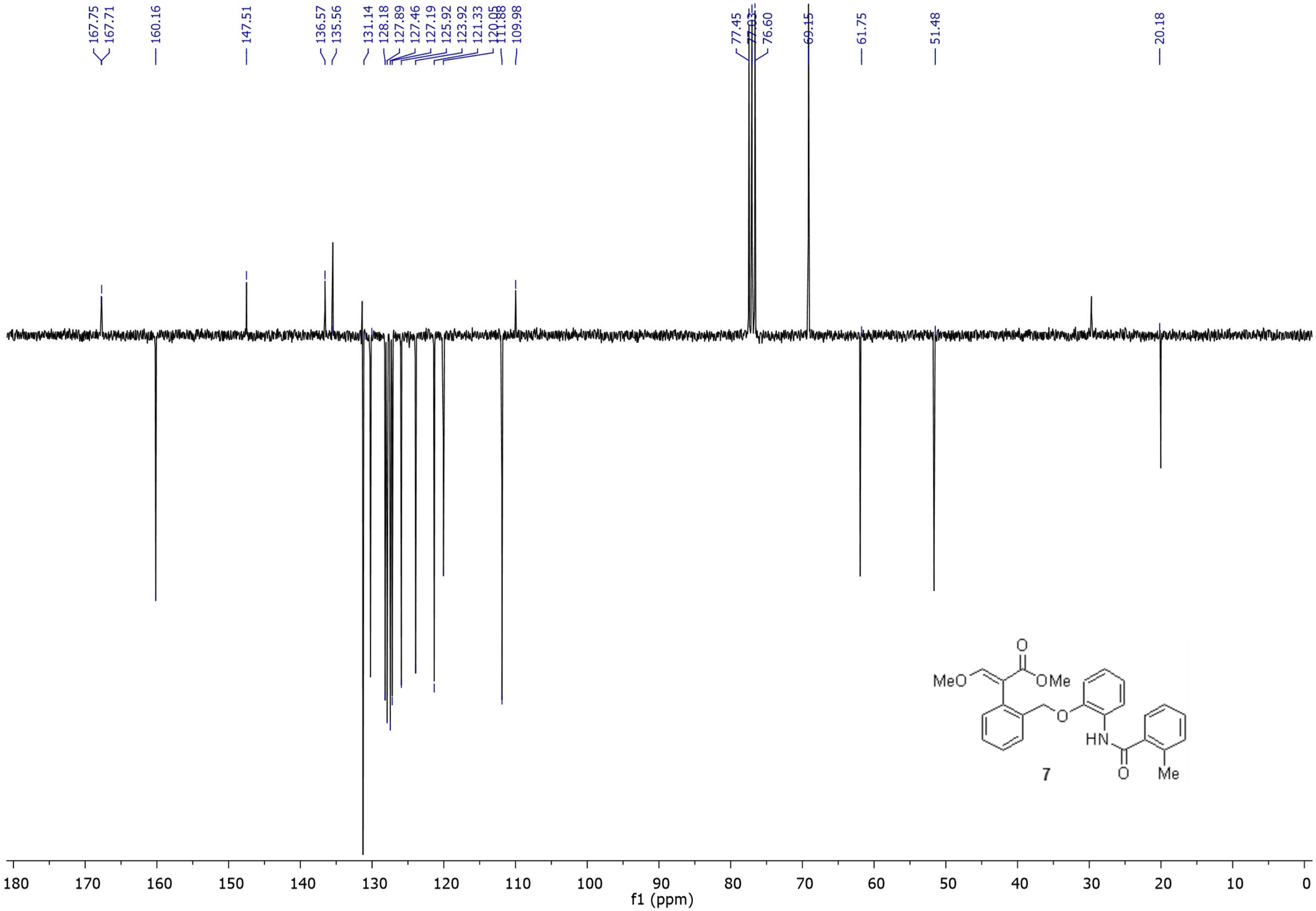

^{1}H-NMR (300 MHz, $CDCl_3$) compound **8**.

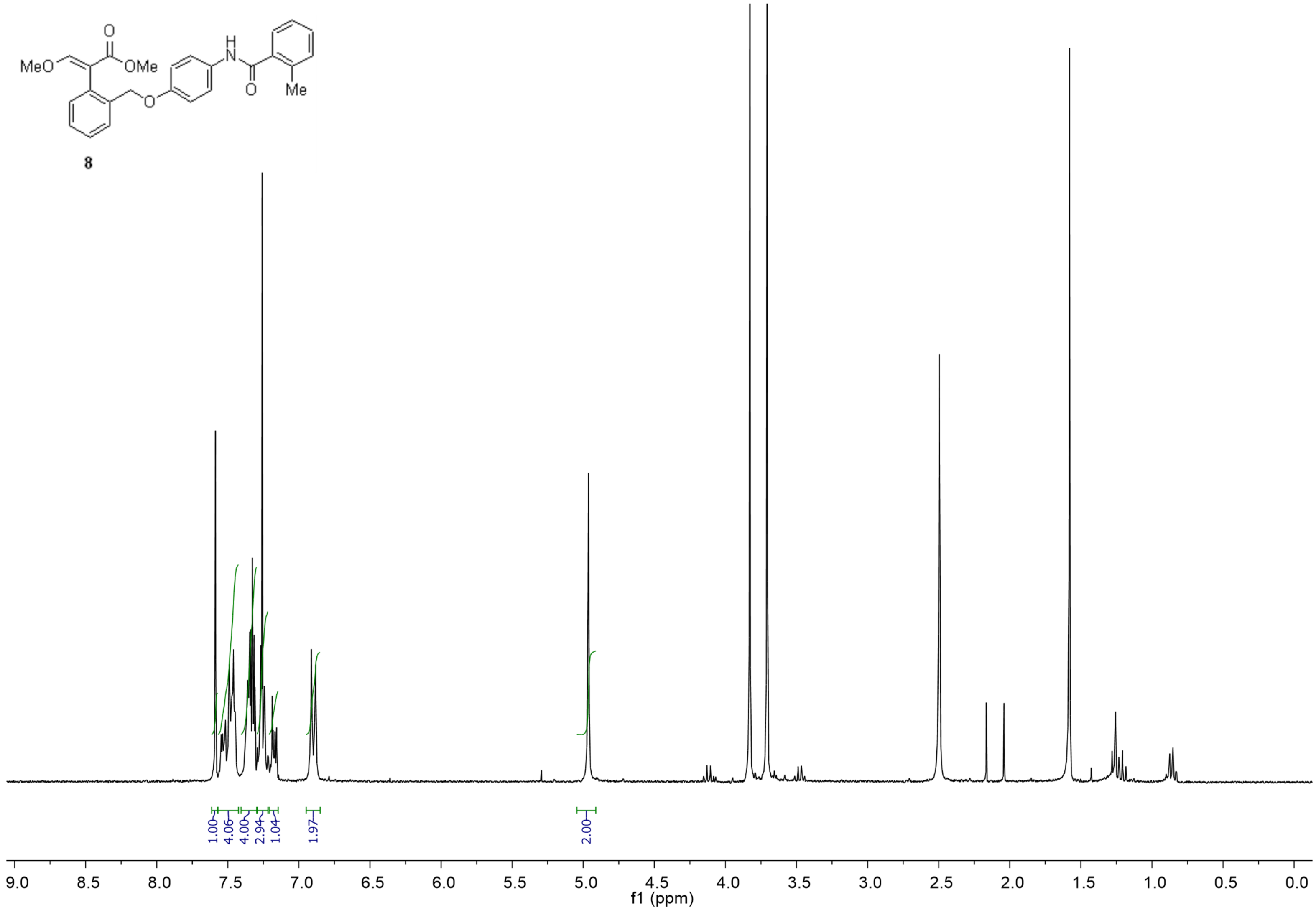

^{13}C-NMR (75 MHz, $CDCl_3$) compound **8**.

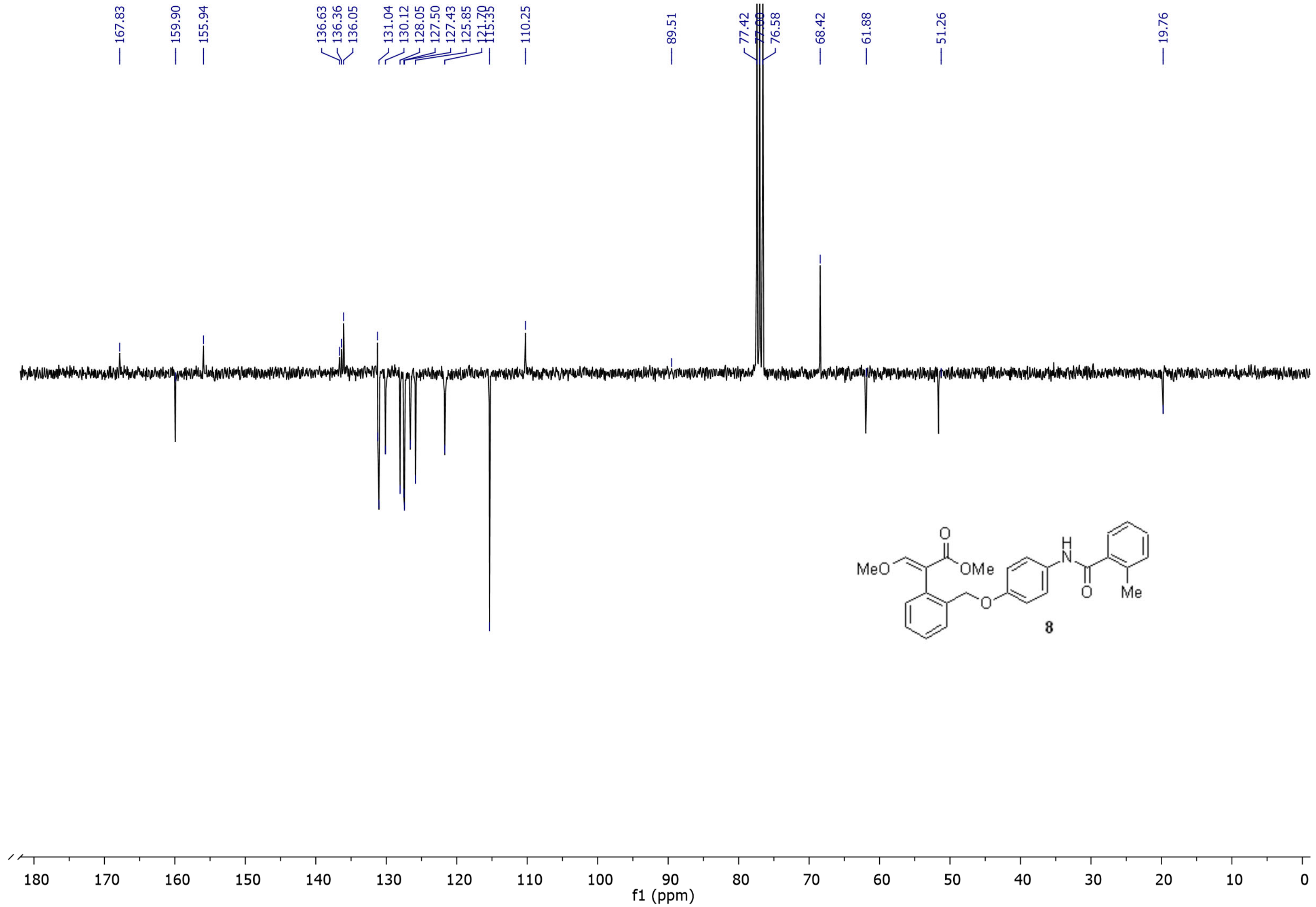

^{1}H-NMR (600 MHz, $CDCl_3$) compound **9**.

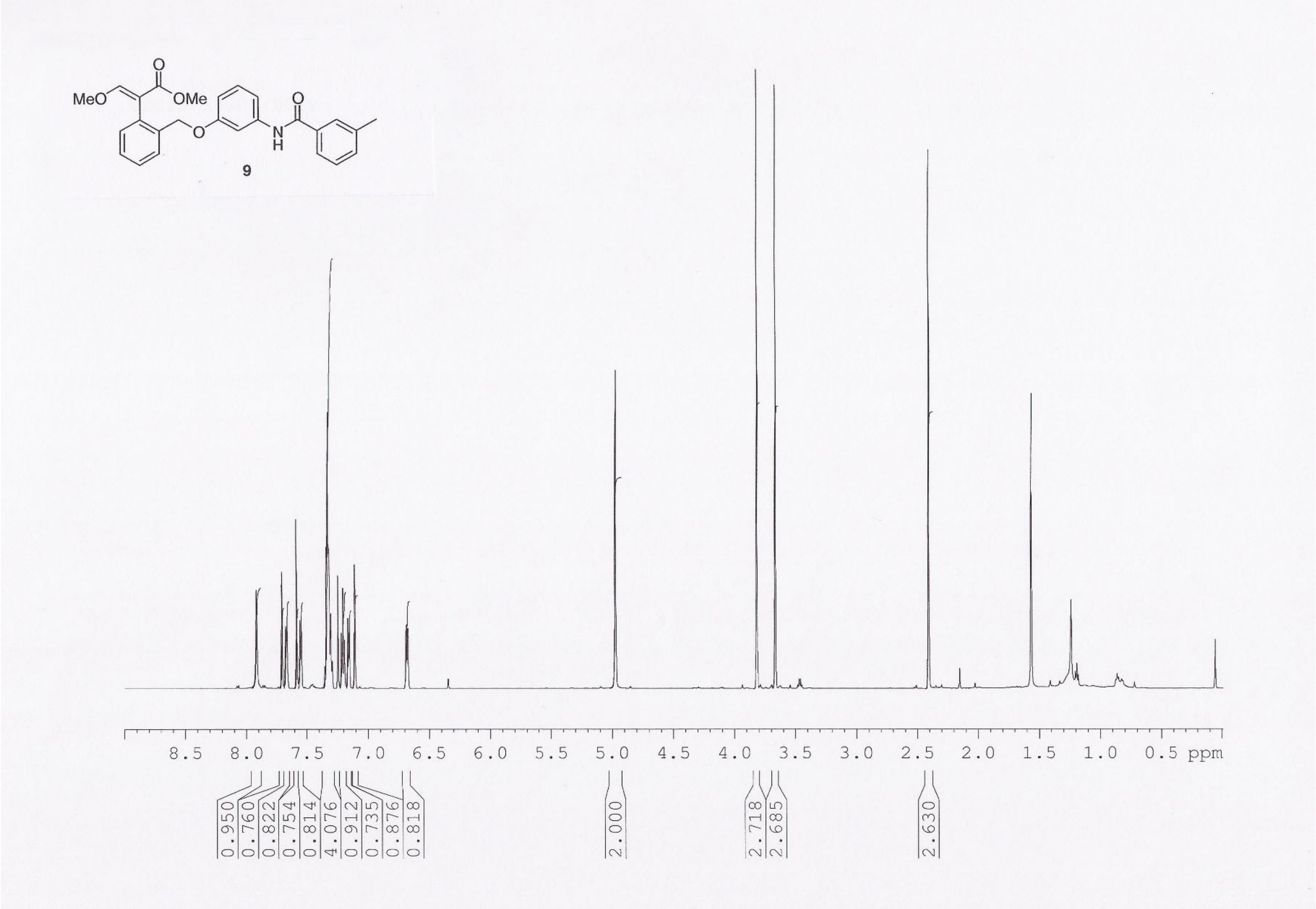

^{13}C-NMR (150 MHz, $CDCl_3$) compound **9**.

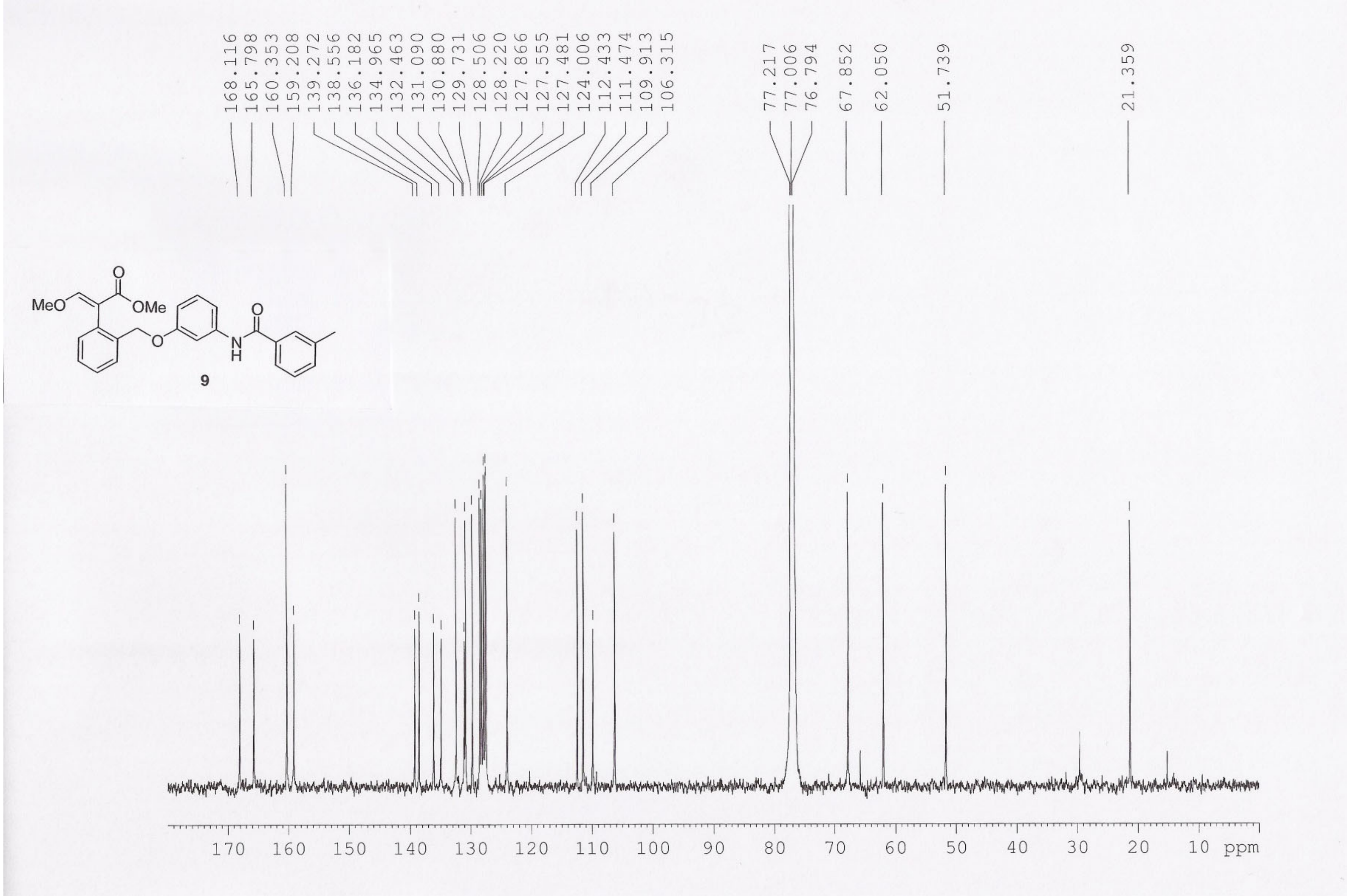

^{1}H-NMR (600 MHz, $CDCl_3$) compound **10**.

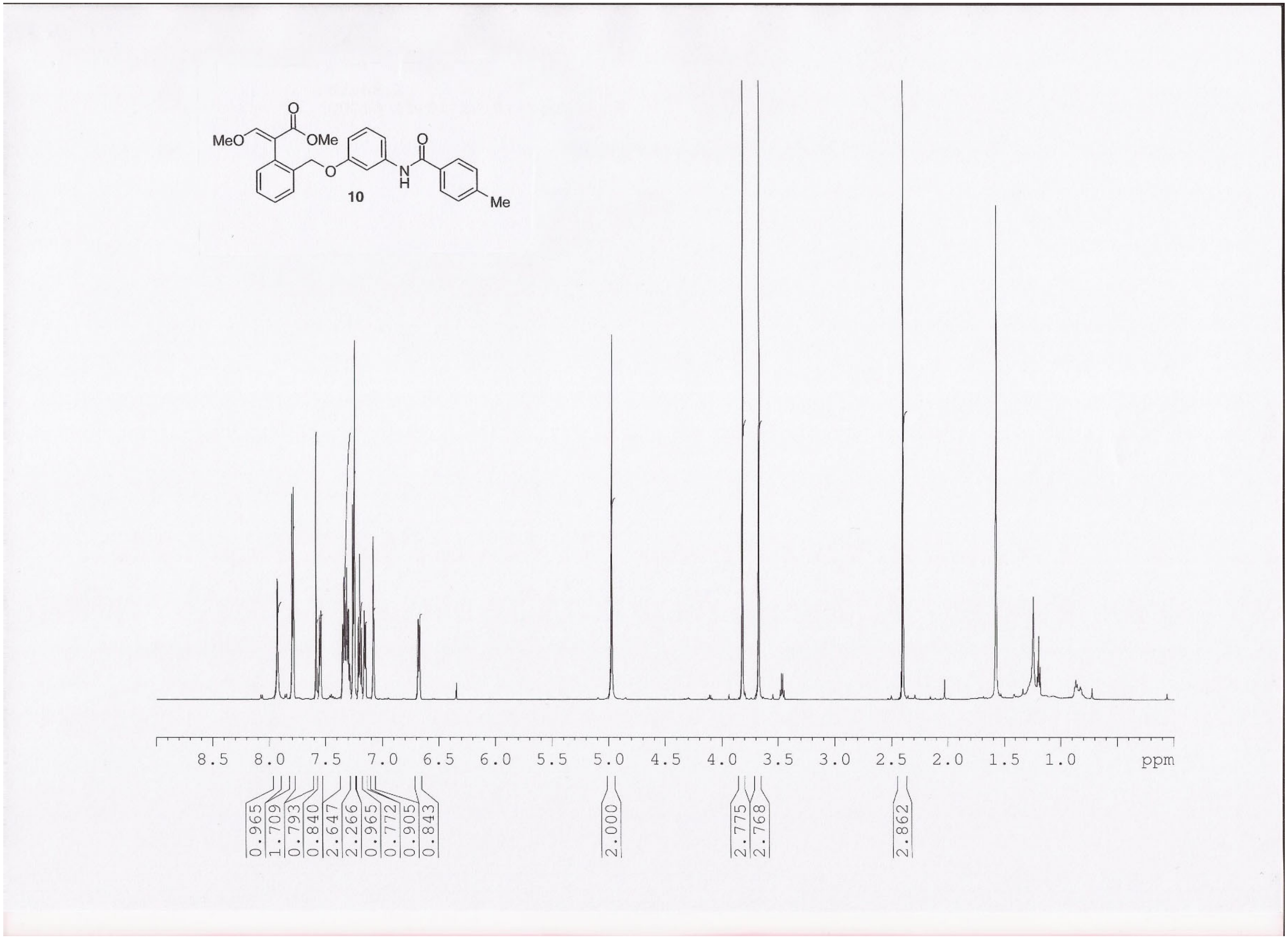
MeO
O
OMe
O
N
H
O
10
Me
8.5
8.0
7.5
7.0
6.5
6.0
5.5
5.0
4.5
4.0
3.5
3.0
2.5
2.0
1.5
1.0
ppm
0.965
1.709
0.793
0.840
2.647
2.266
0.965
0.772
0.905
0.843
2.000
2.775
2.768
2.862

^{13}C-NMR (150 MHz, $CDCl_3$) compound **10**.

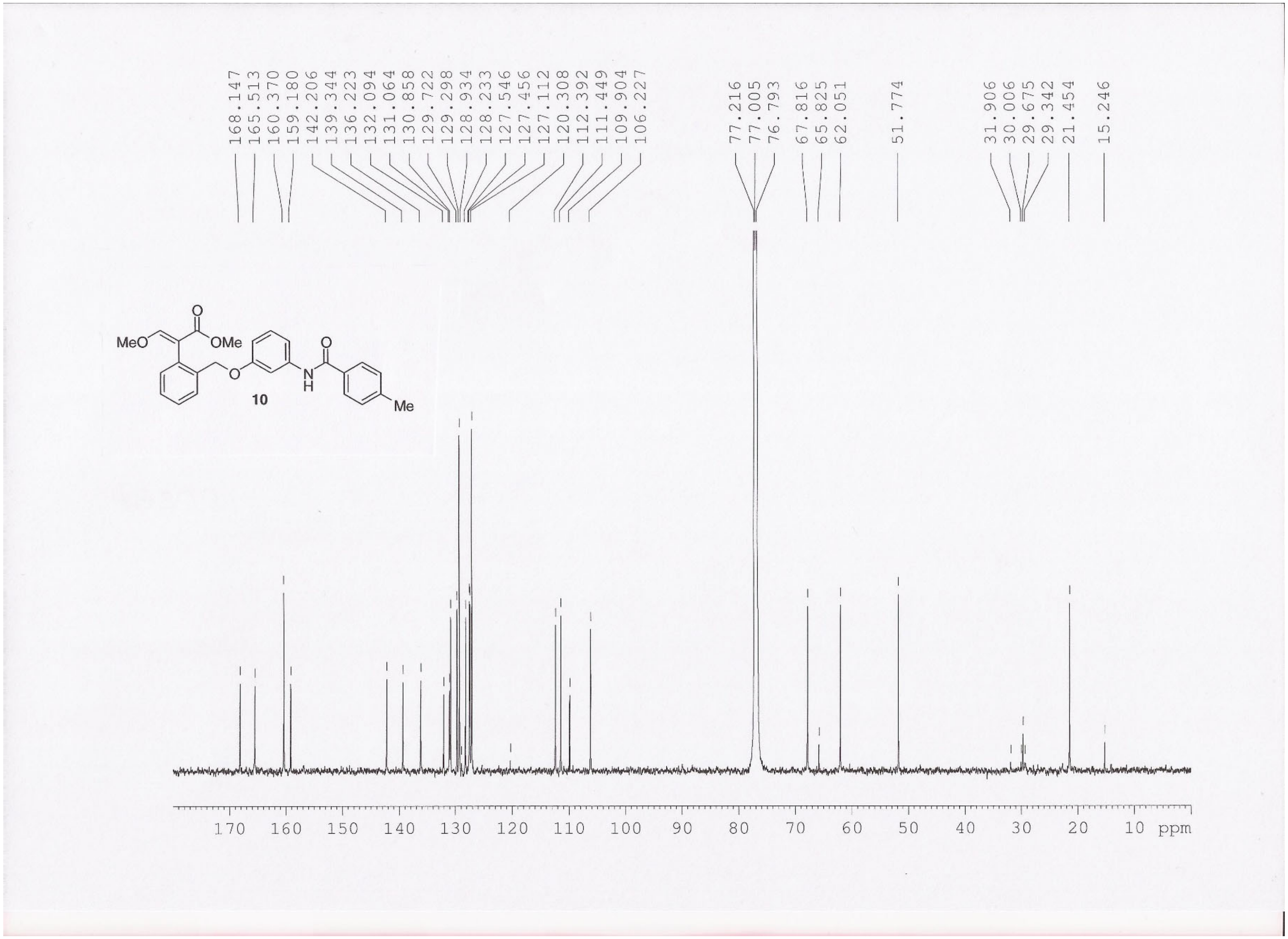

^{1}H-NMR (300 MHz, $CDCl_3$) compound **11a**.

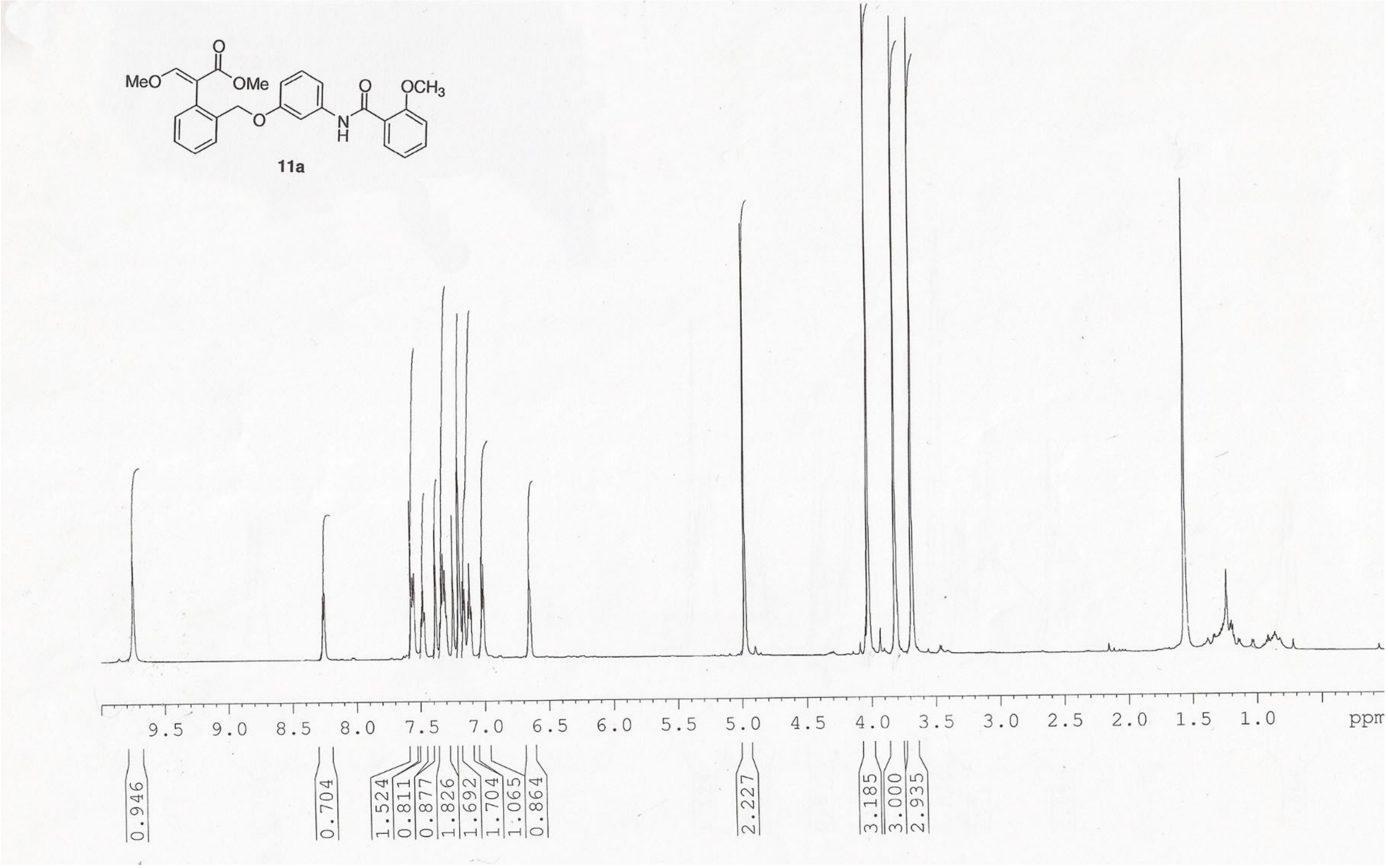

^{13}C-NMR (75 MHz, $CDCl_3$) compound **11a**.

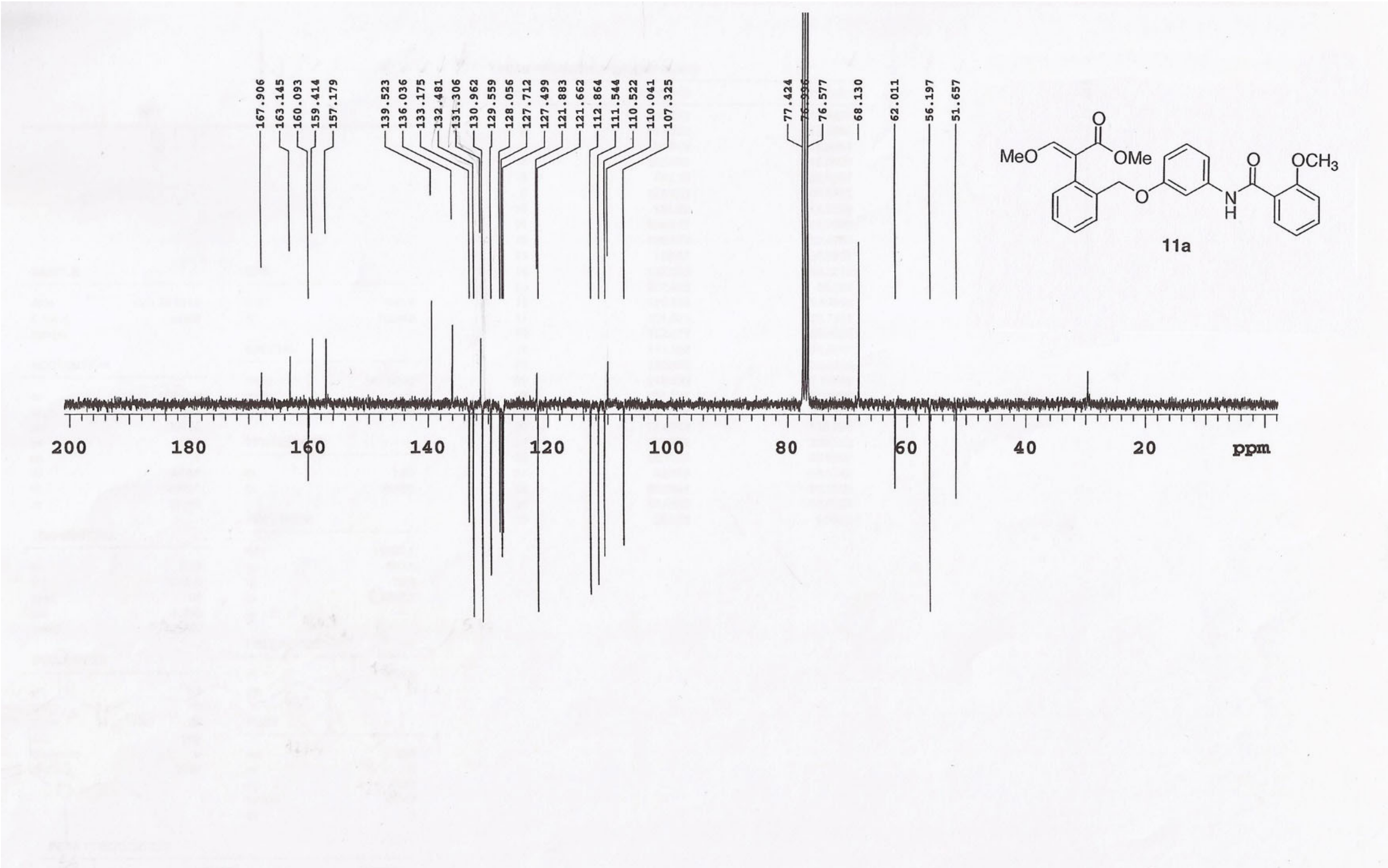

^{1}H-NMR (300 MHz, $CDCl_3$) compound **11b**.

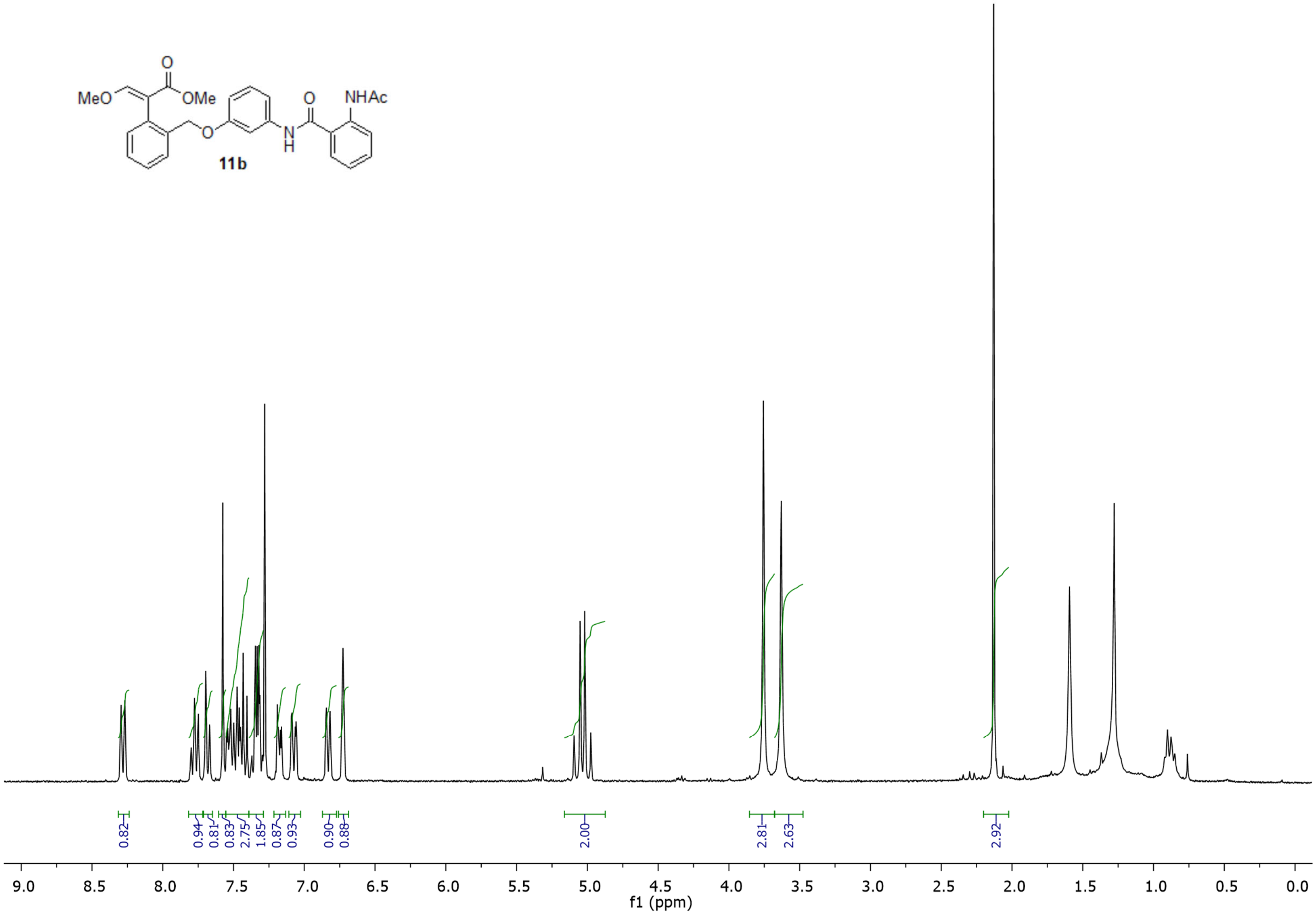

^{13}C-NMR (75 MHz, $CDCl_3$) compound **11b**.

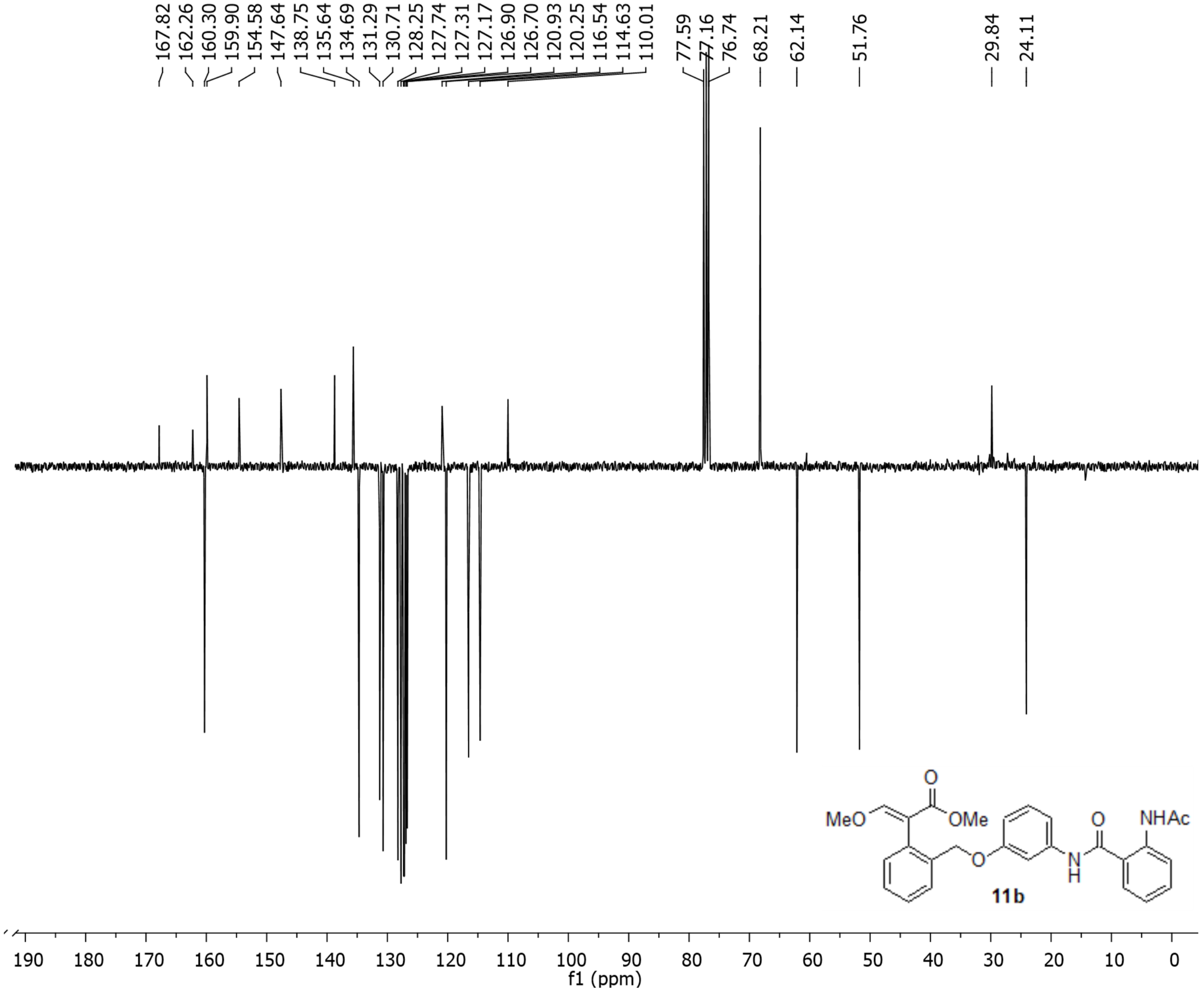

^{1}H-NMR (300 MHz, $CDCl_3$) compound **11c**.

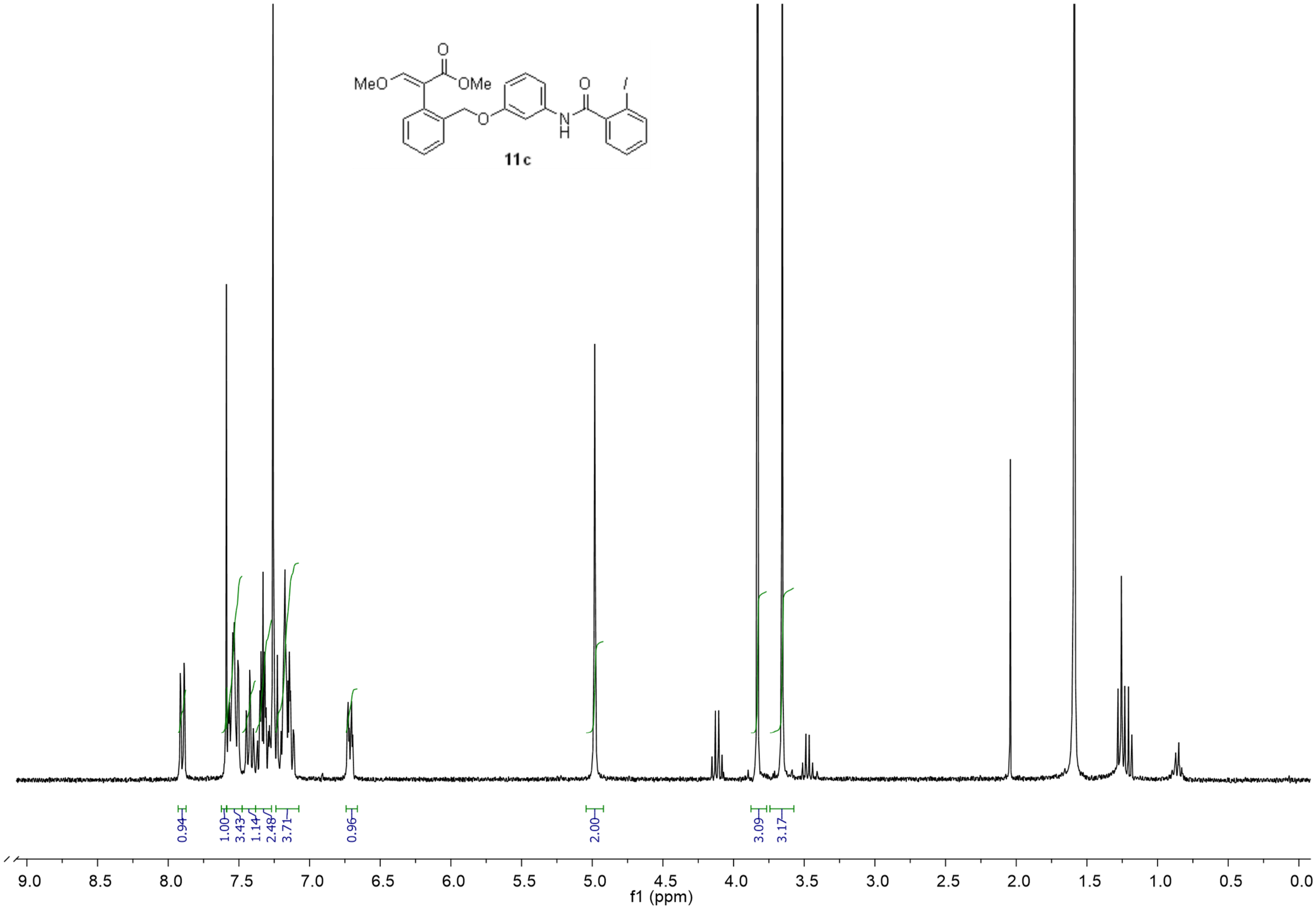

^{13}C-NMR (75 MHz, $CDCl_3$) compound **11c**.

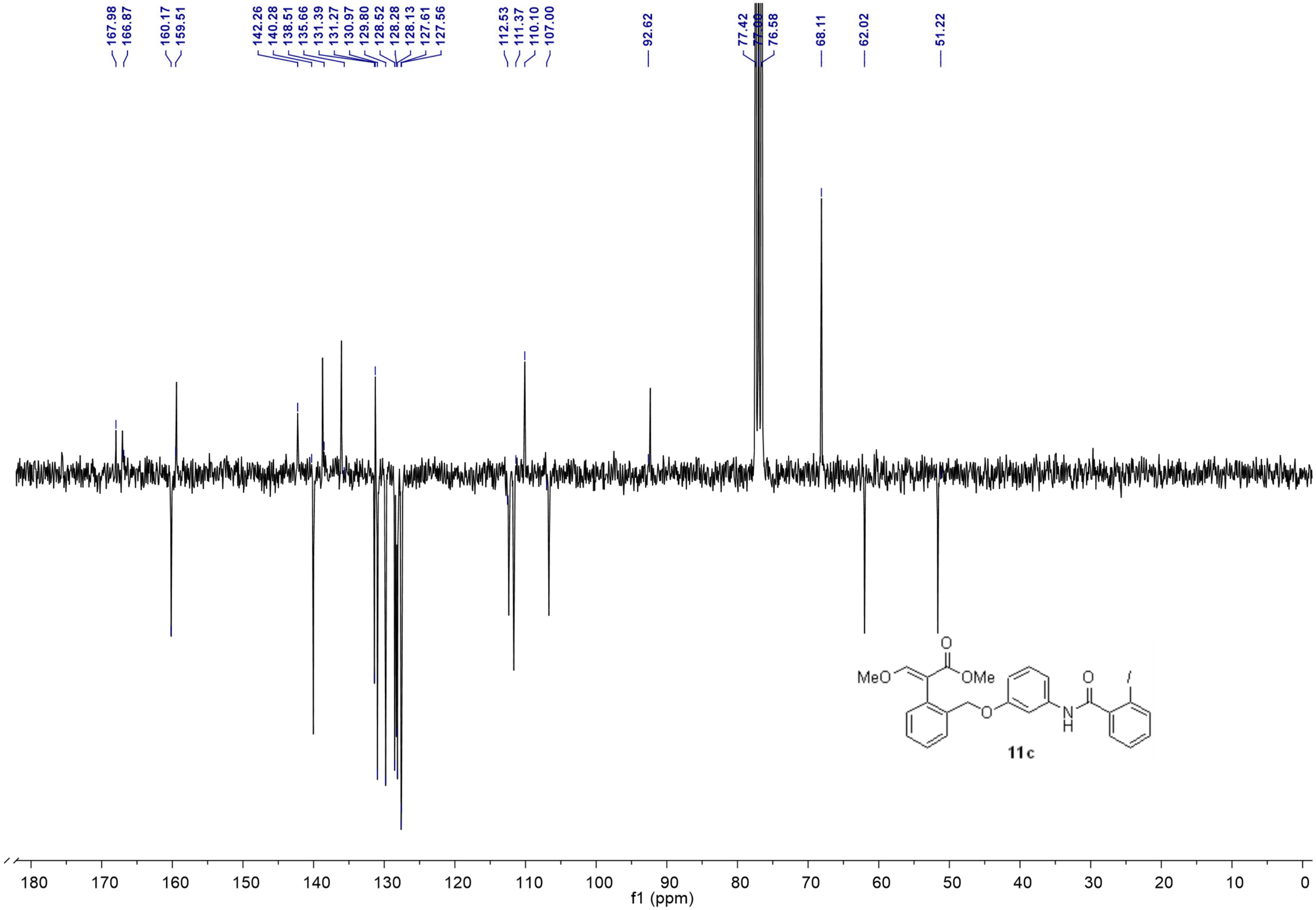

^{1}H-NMR (600 MHz, $CDCl_3$) compound **11d**.

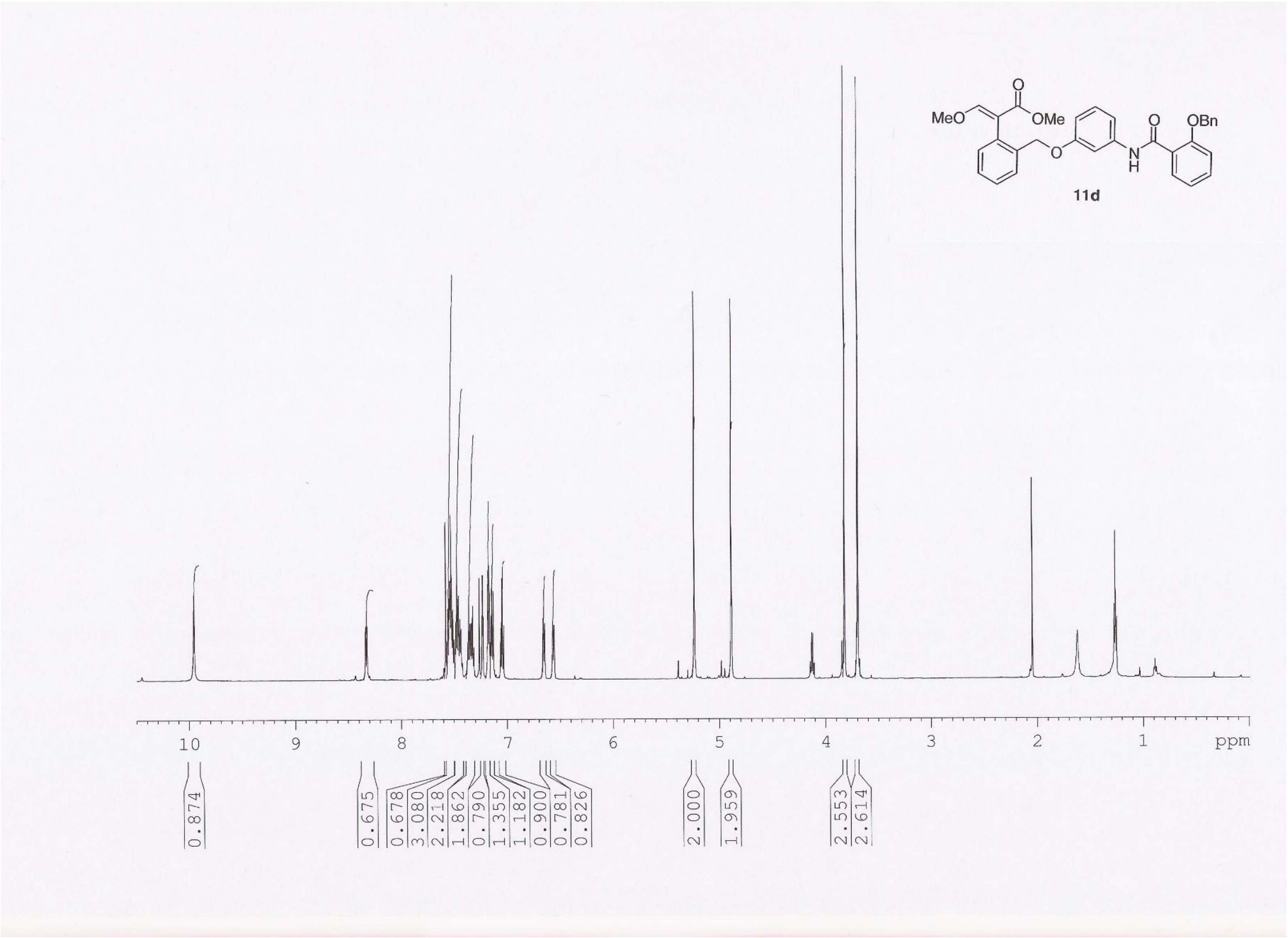

^{13}C-NMR (150 MHz, $CDCl_3$) compound **11d**.

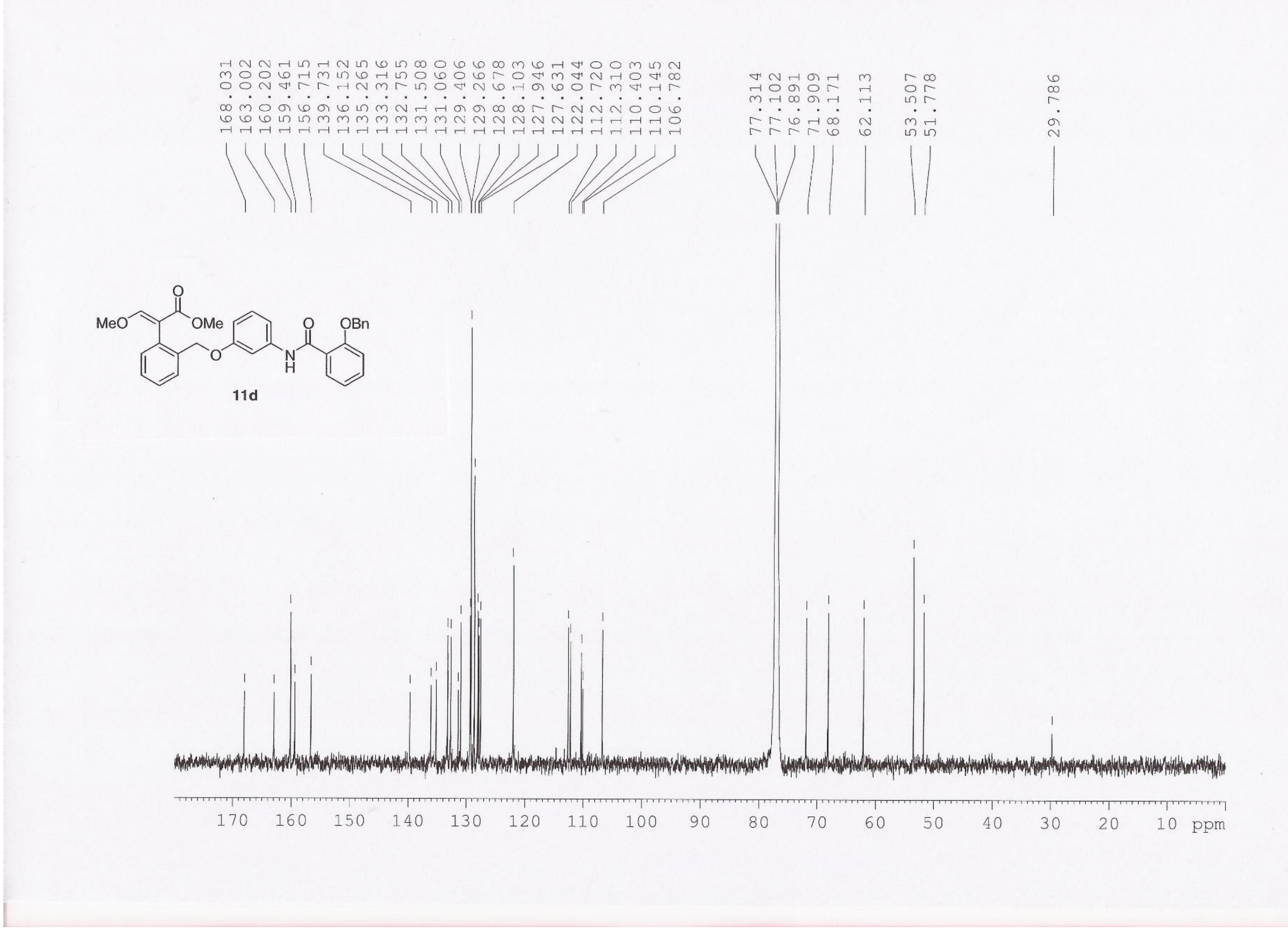

^{1}H-NMR (600 MHz, $CDCl_3$) compound **11e**.

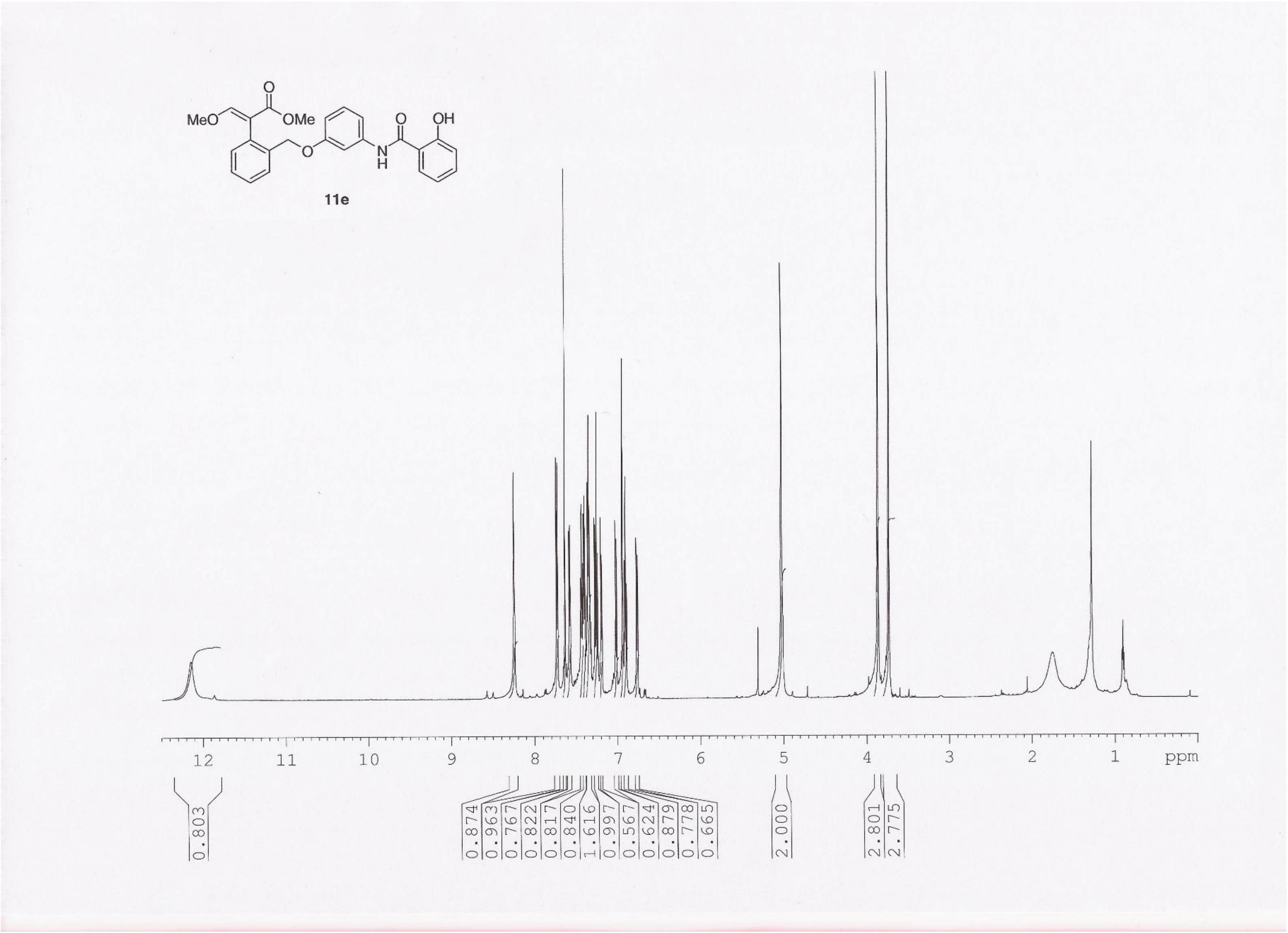

^{13}C-NMR (150 MHz, $CDCl_3$) compound **11e**.

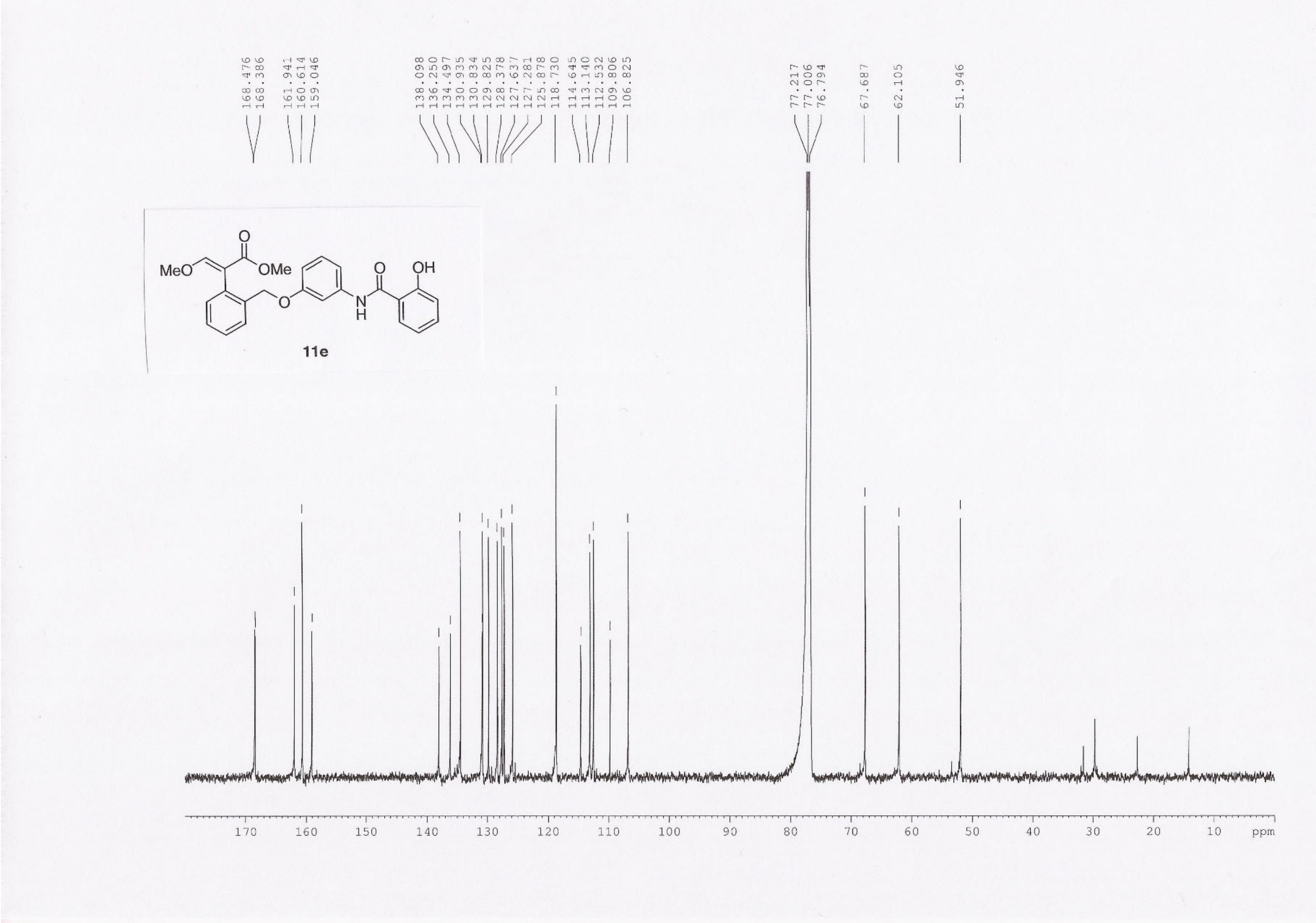

^{1}H-NMR (600 MHz, CDCl3) compound **11f**.

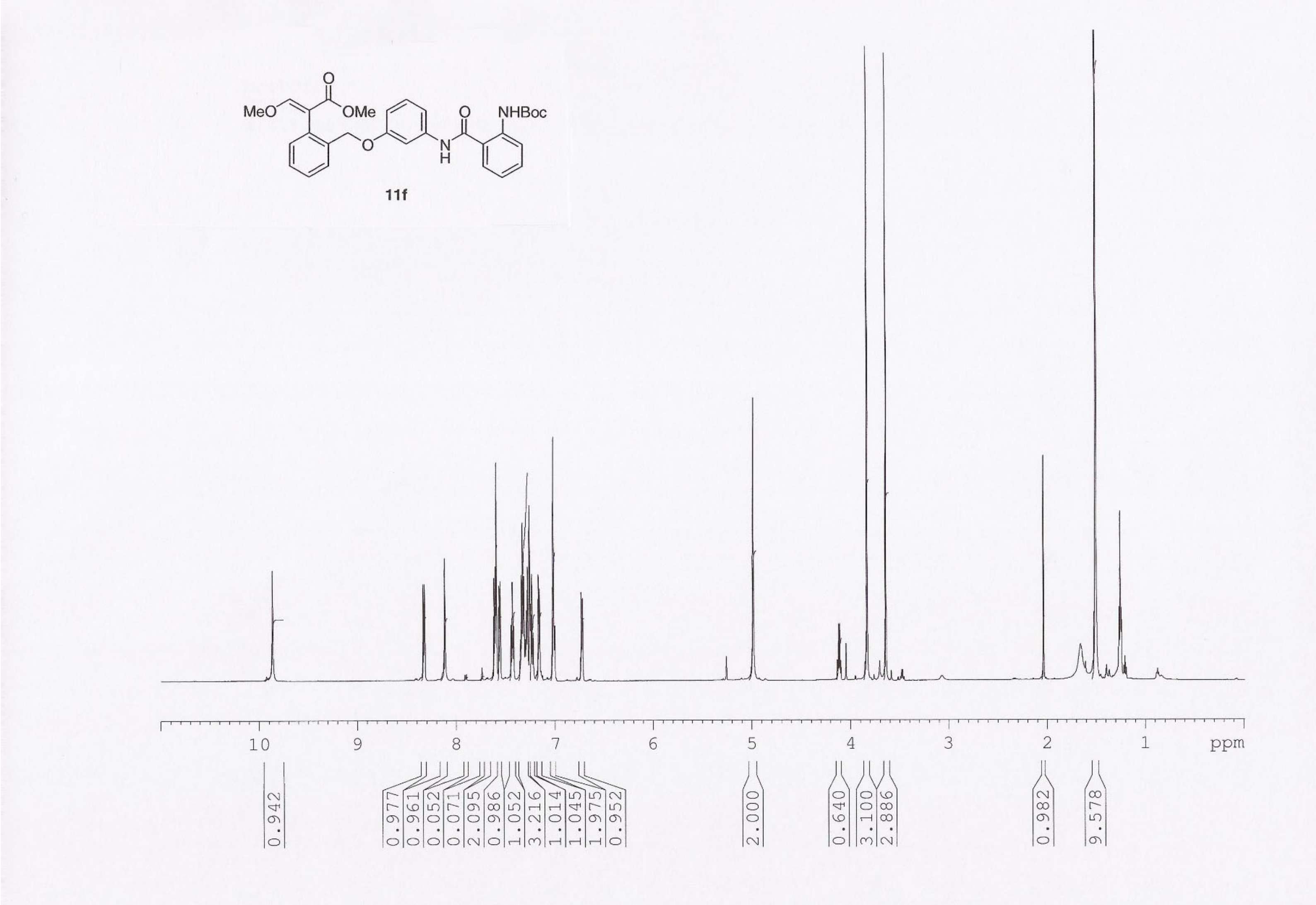

^{13}C-NMR (150 MHz, $CDCl_3$) compound **11f**.

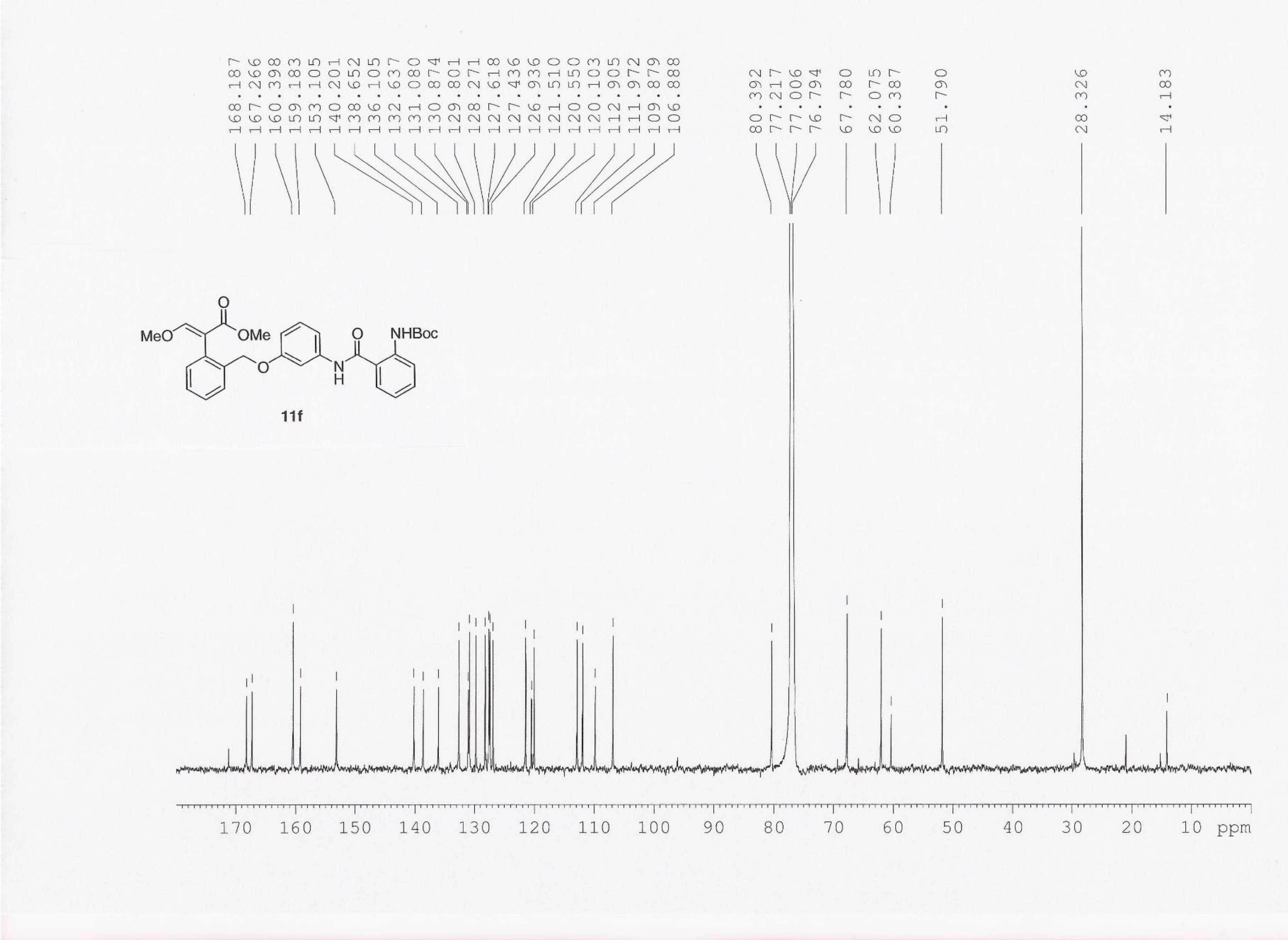

^{1}H-NMR (600 MHz, $CDCl_3$) compound **11g**.

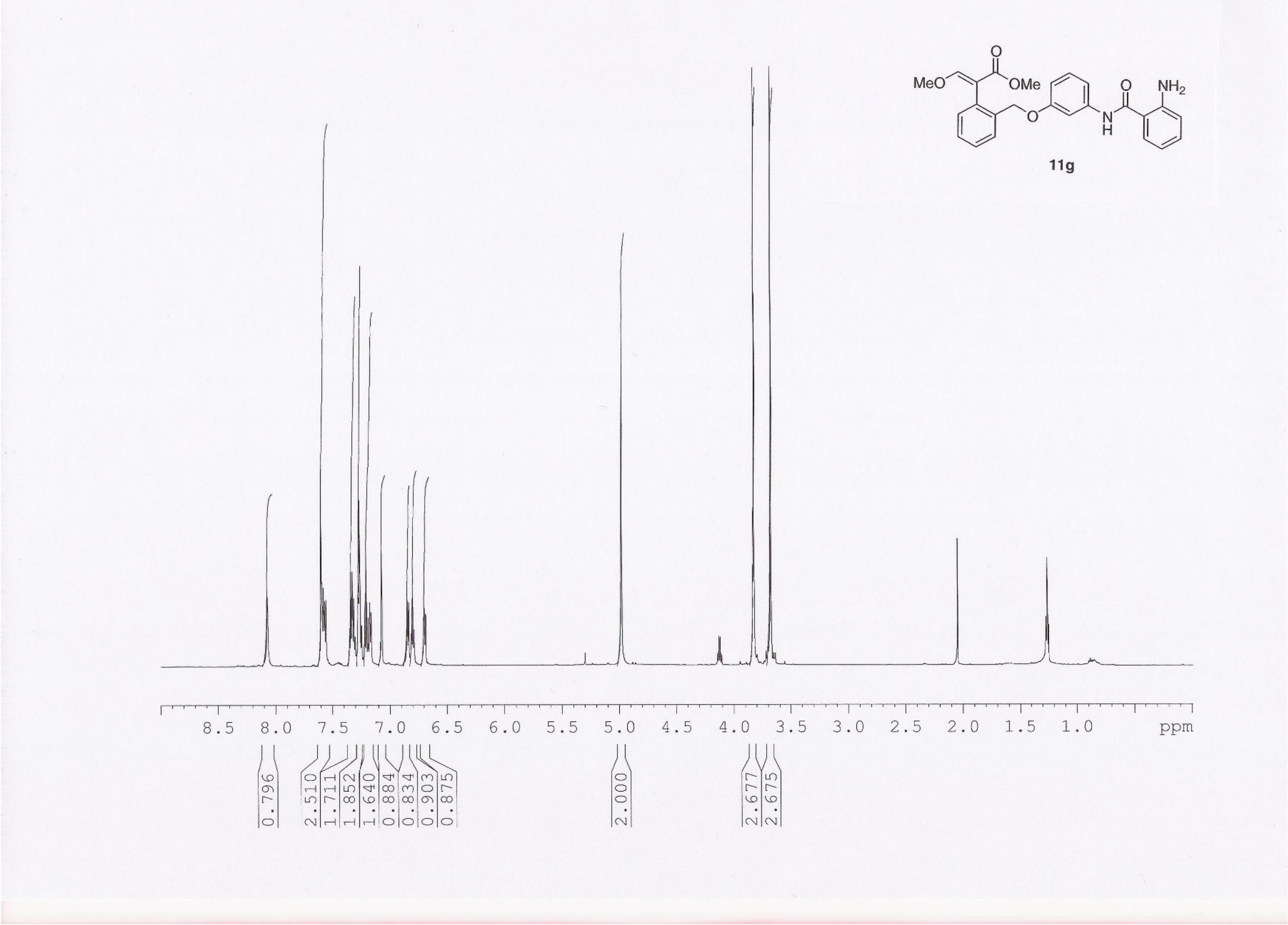

^{13}C-NMR (150 MHz, $CDCl_3$) compound **11g**.

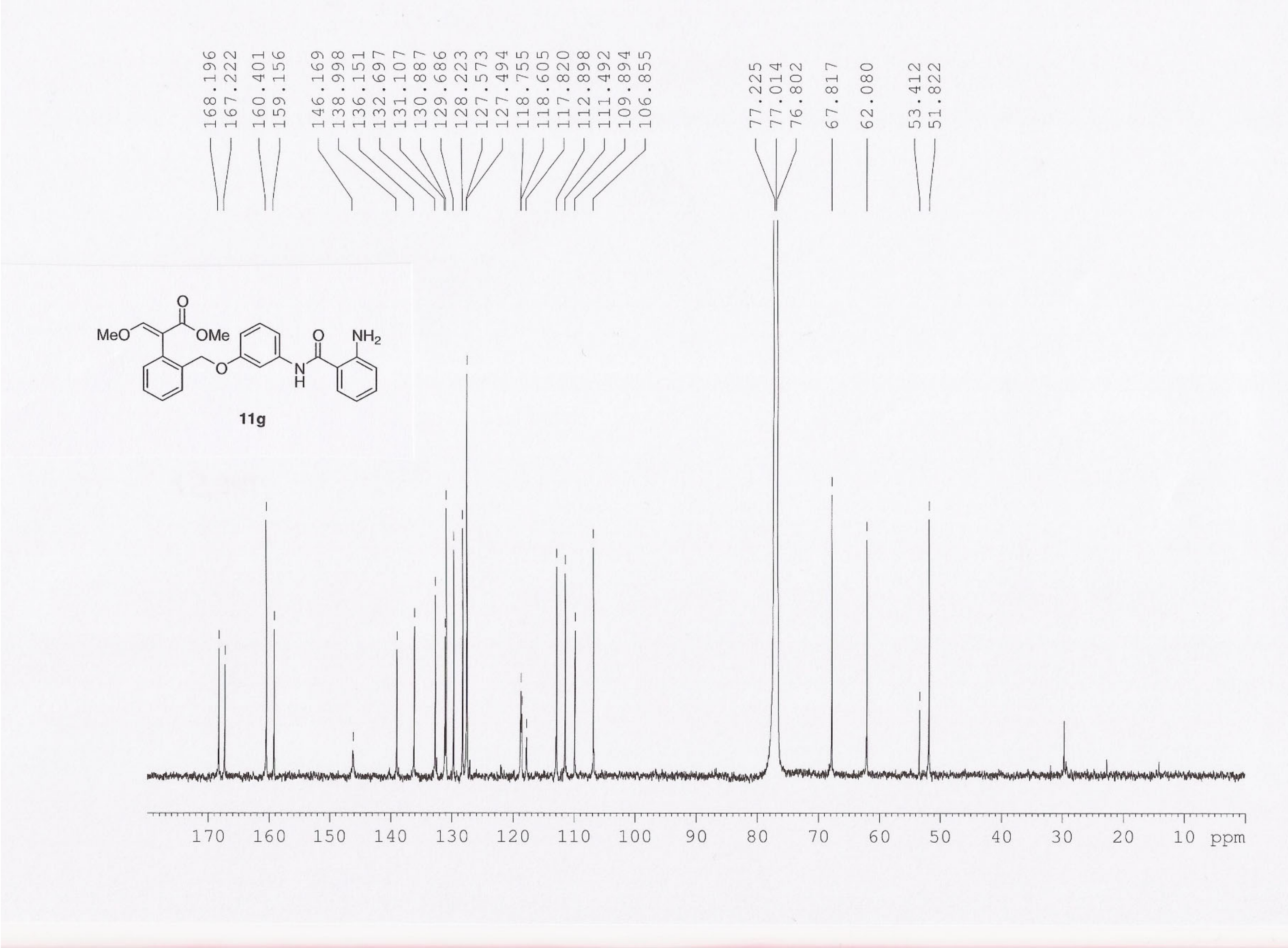

^{1}H-NMR (600 MHz, $CDCl_3$) compound **12**.

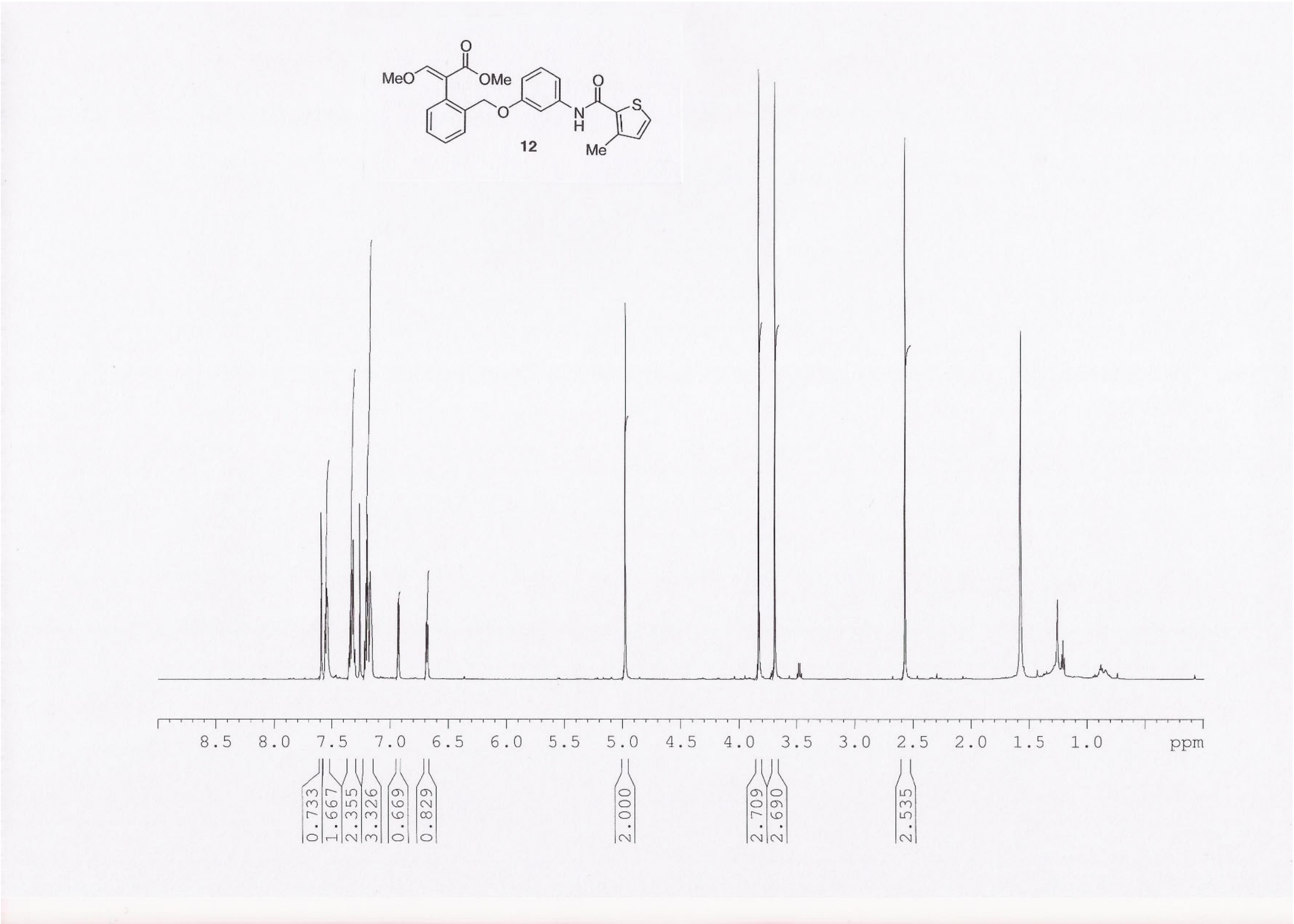

^{13}C-NMR (150 MHz, $CDCl_3$) compound **12**.

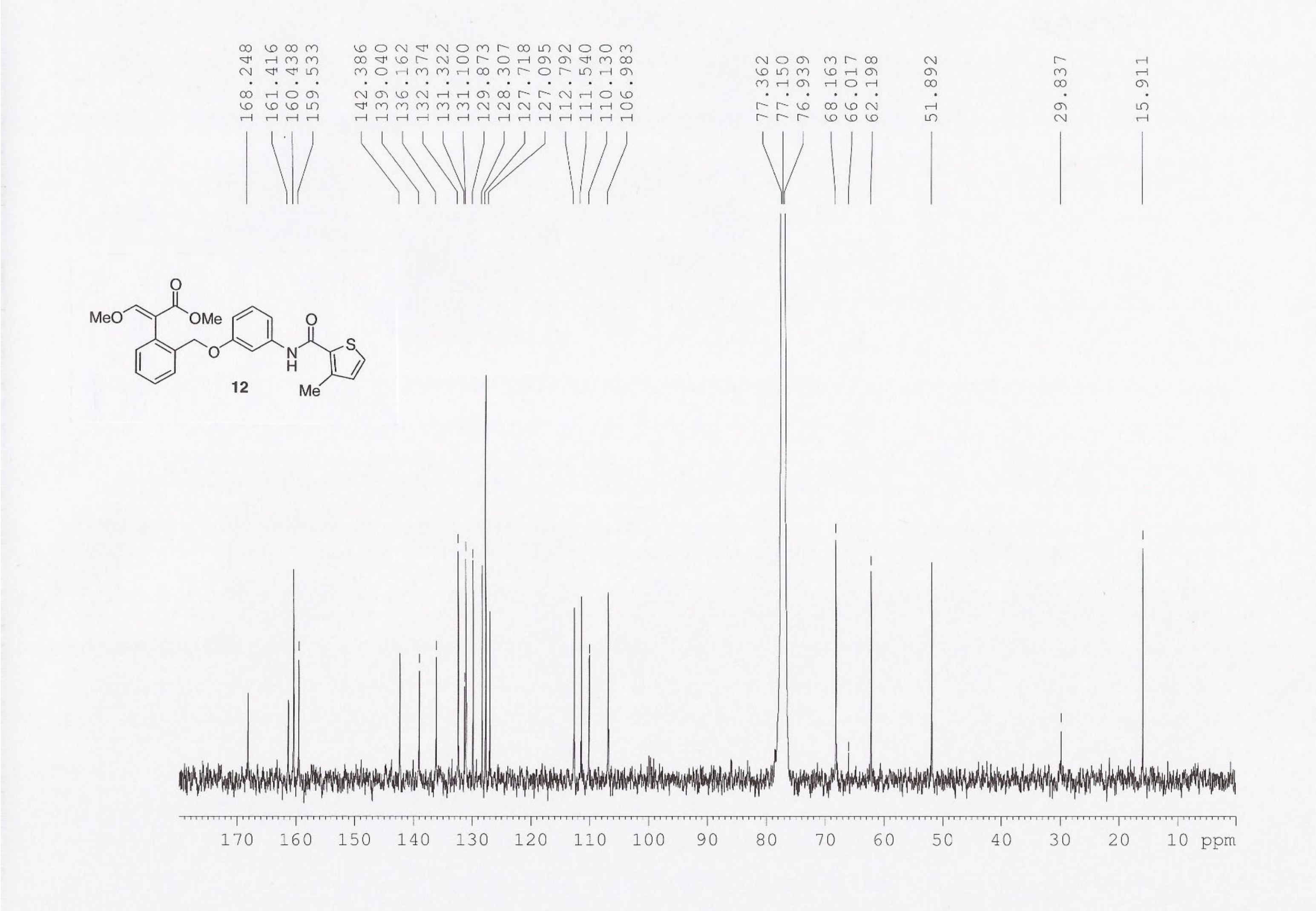

^{1}H-NMR (300 MHz, $CDCl_3$) compound **13**.

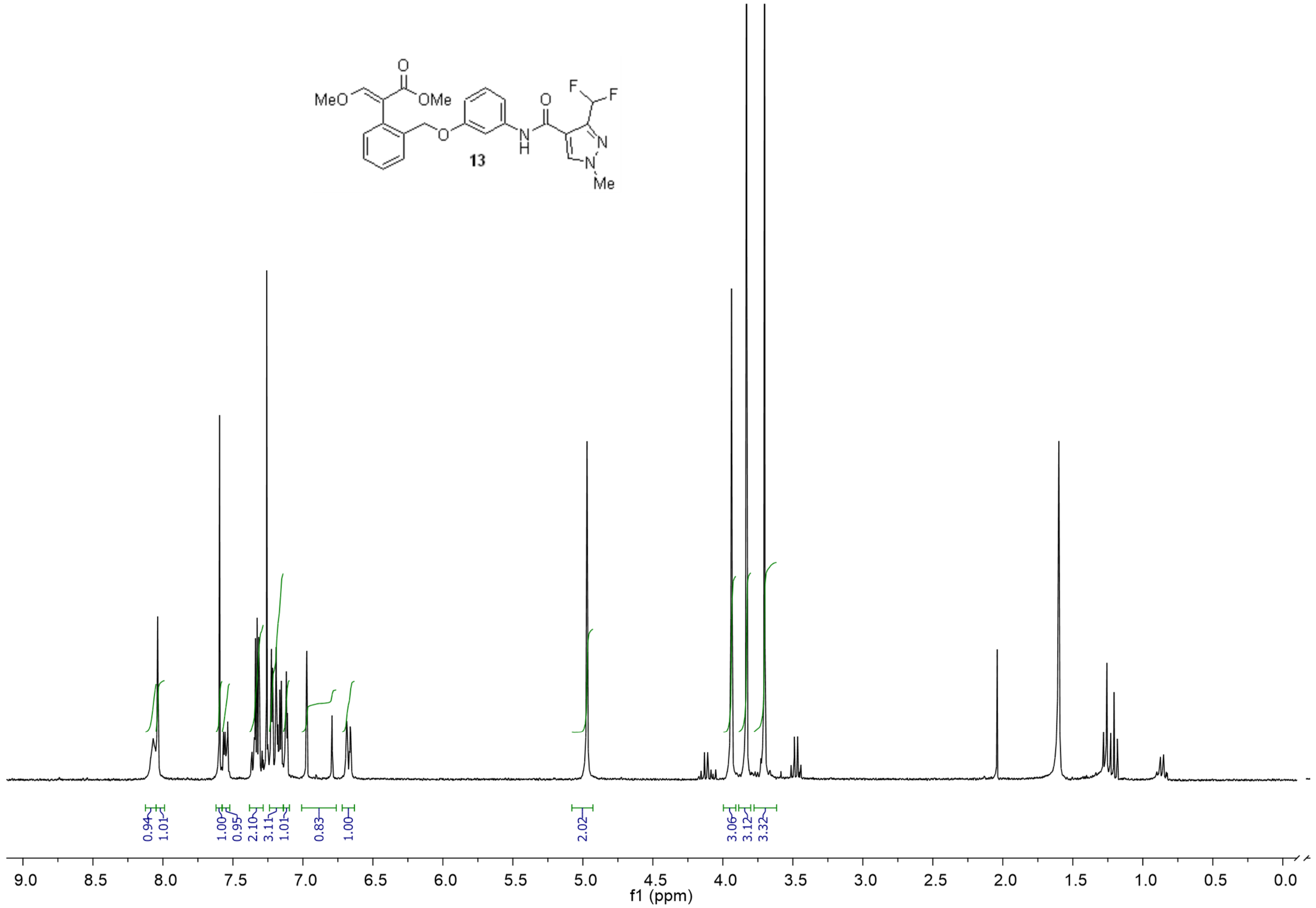

^{13}C-NMR (75 MHz, $CDCl_3$) compound **13**.

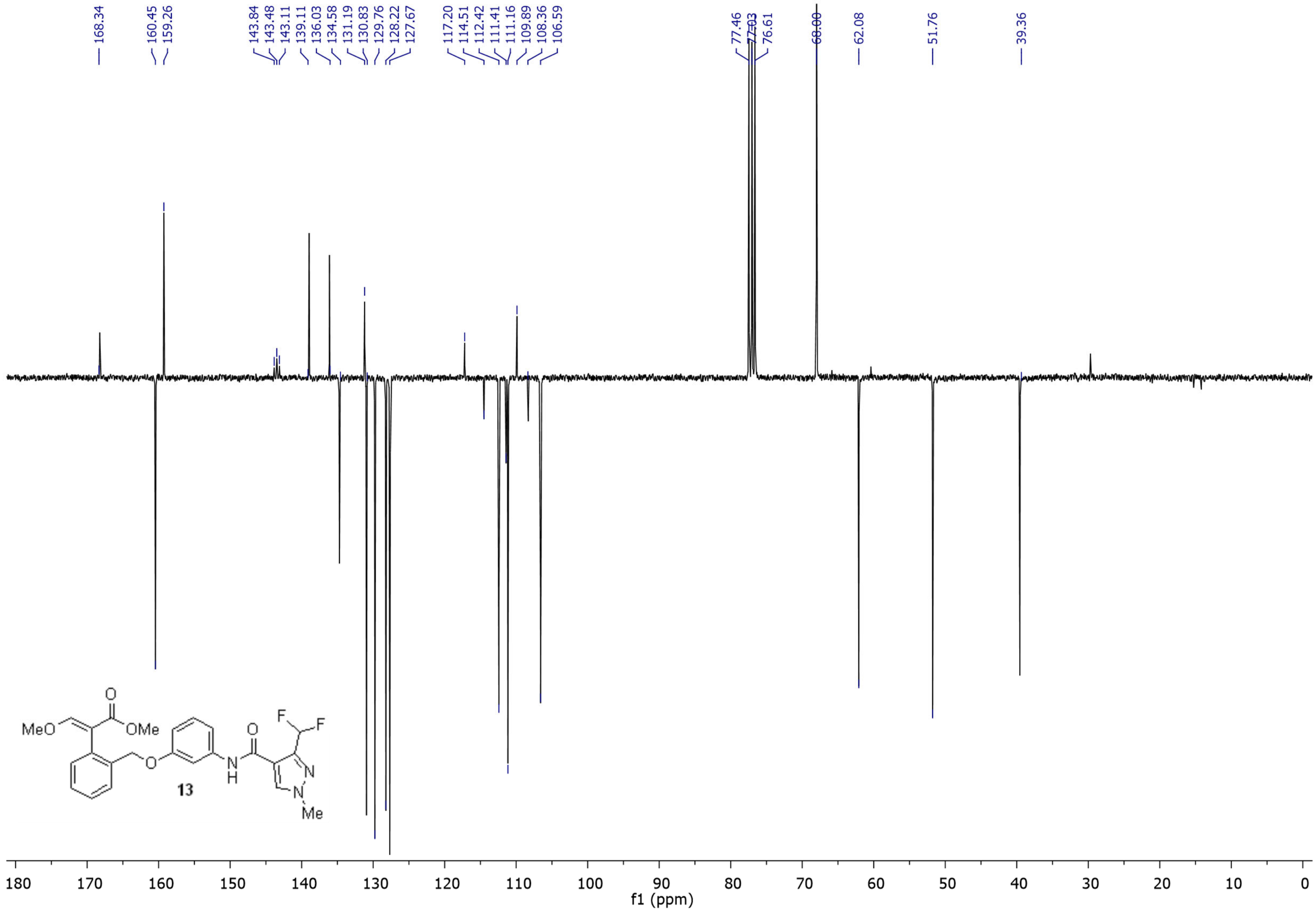

^{1}H-NMR (600 MHz, $CDCl_3$) compound **14a.**

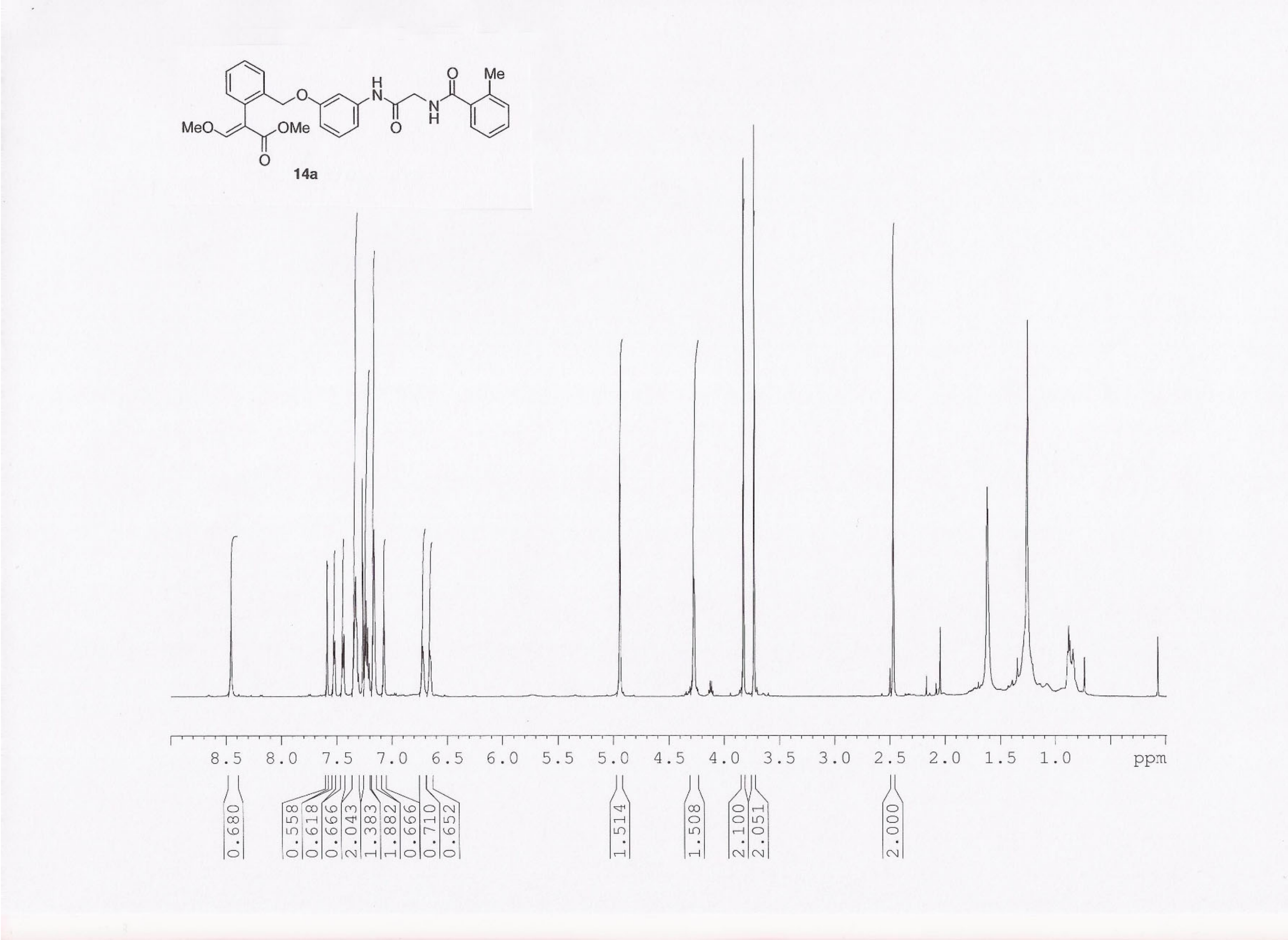

^{13}C-NMR (150 MHz, $CDCl_3$) compound **14a.**

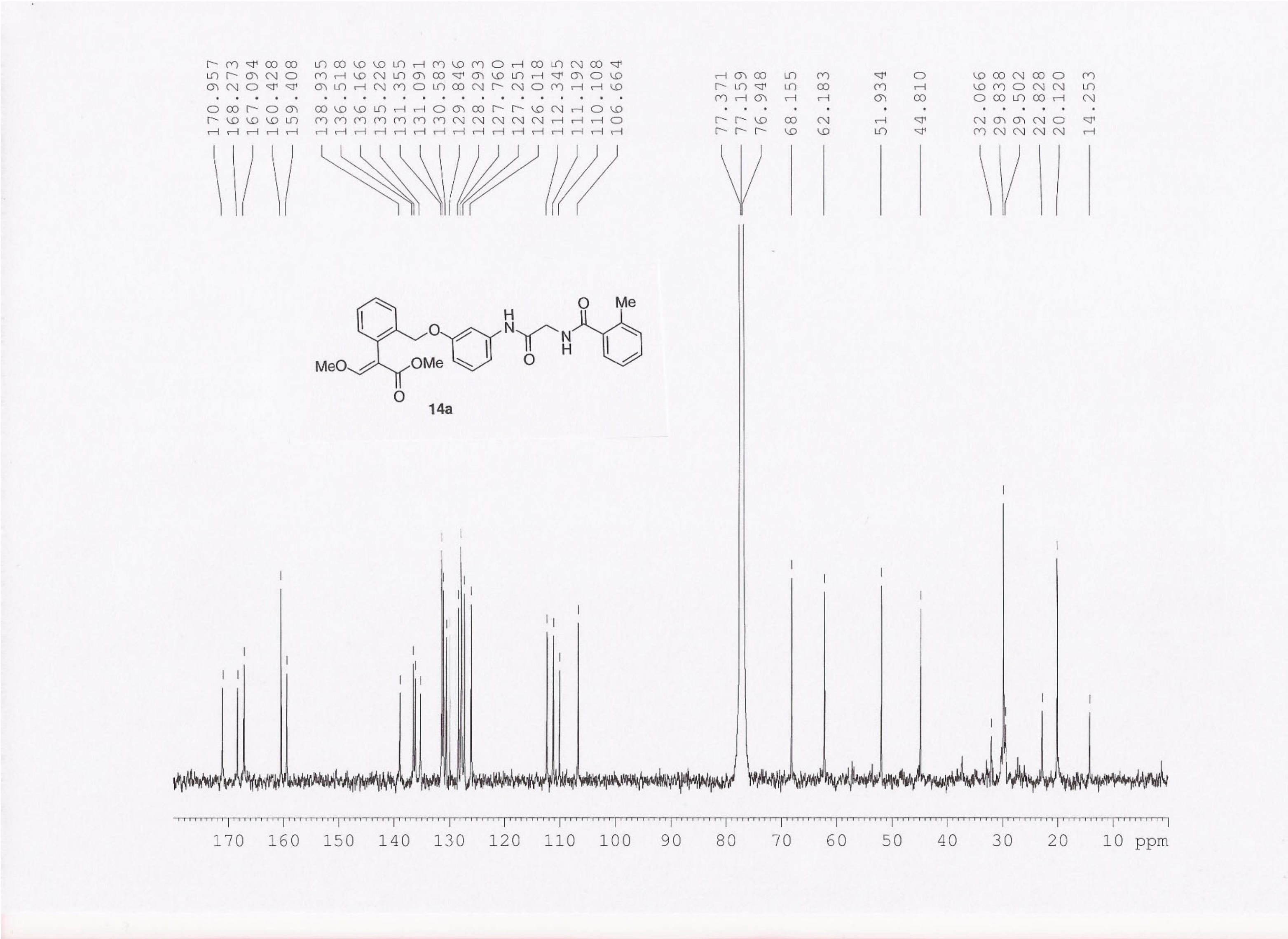

^{1}H-NMR (300 MHz, $CDCl_3$) compound **14b.**

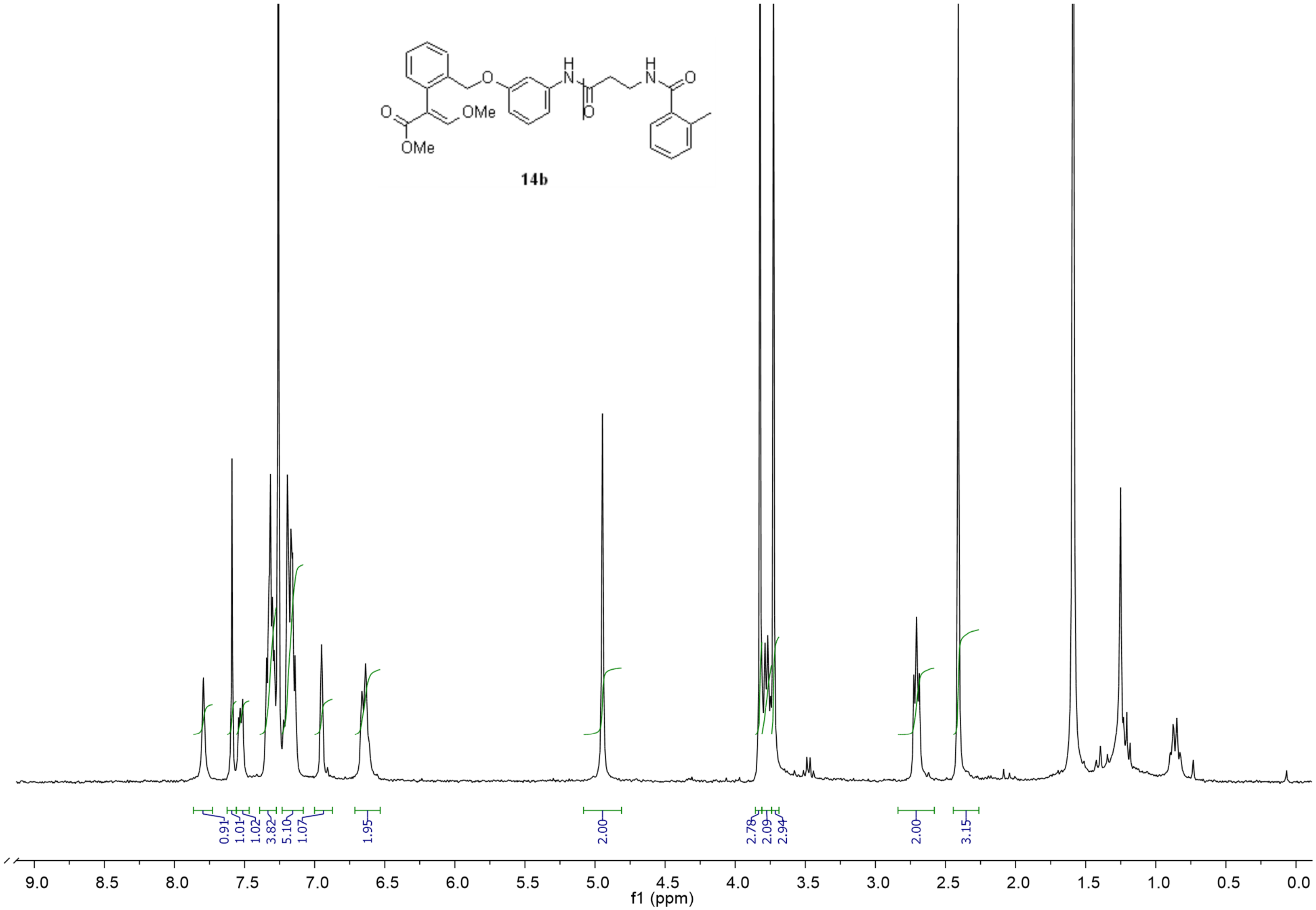

^{13}C-NMR (300 MHz, $CDCl_3$) compound **14b.**

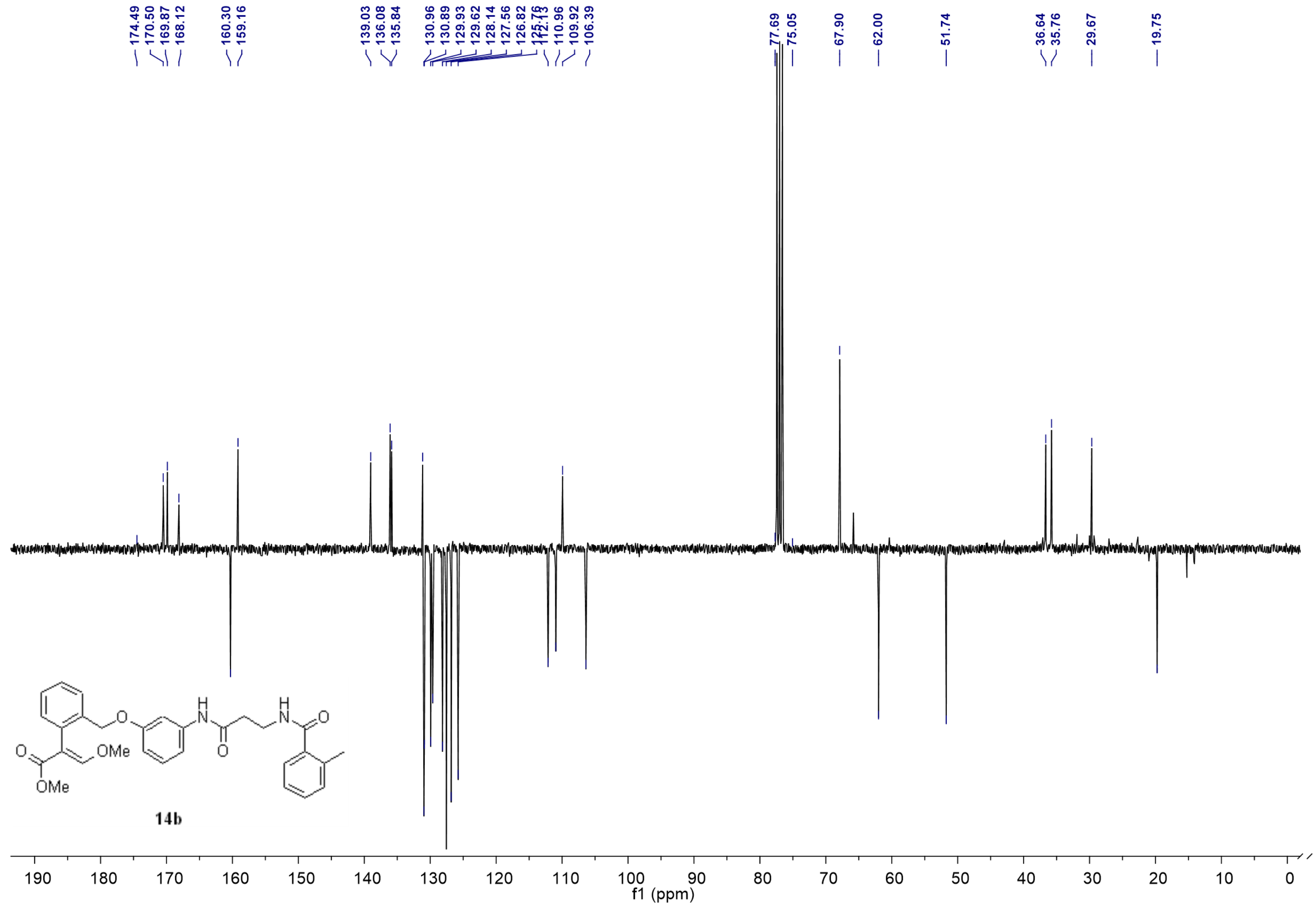

^{1}H-NMR (300 MHz, CDCl3) compound **14c.**

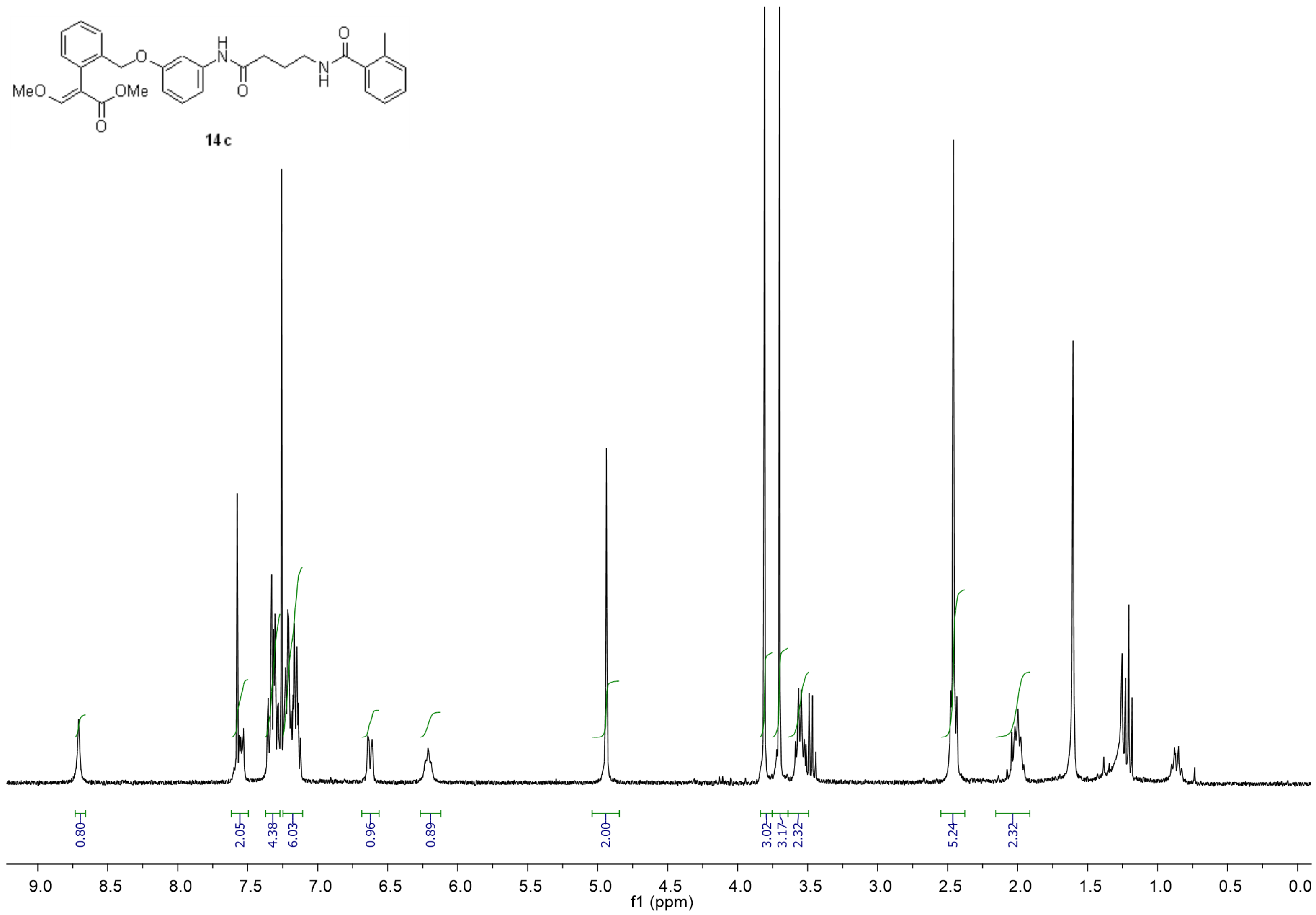

^{13}C-NMR (75 MHz, $CDCl_3$) compound **14c.**

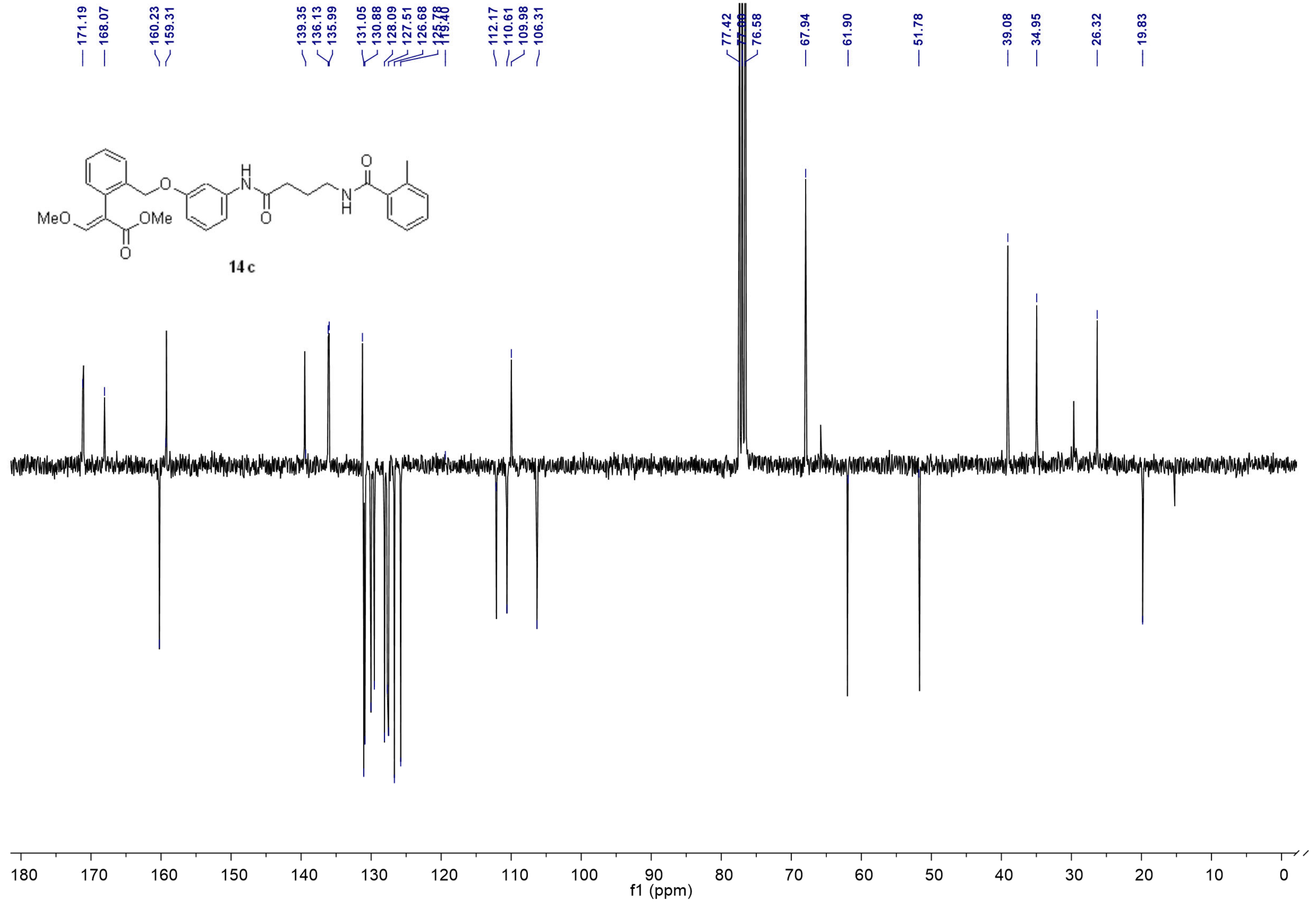

^{1}H-NMR (300 MHz, $CDCl_3$) compound **15.**

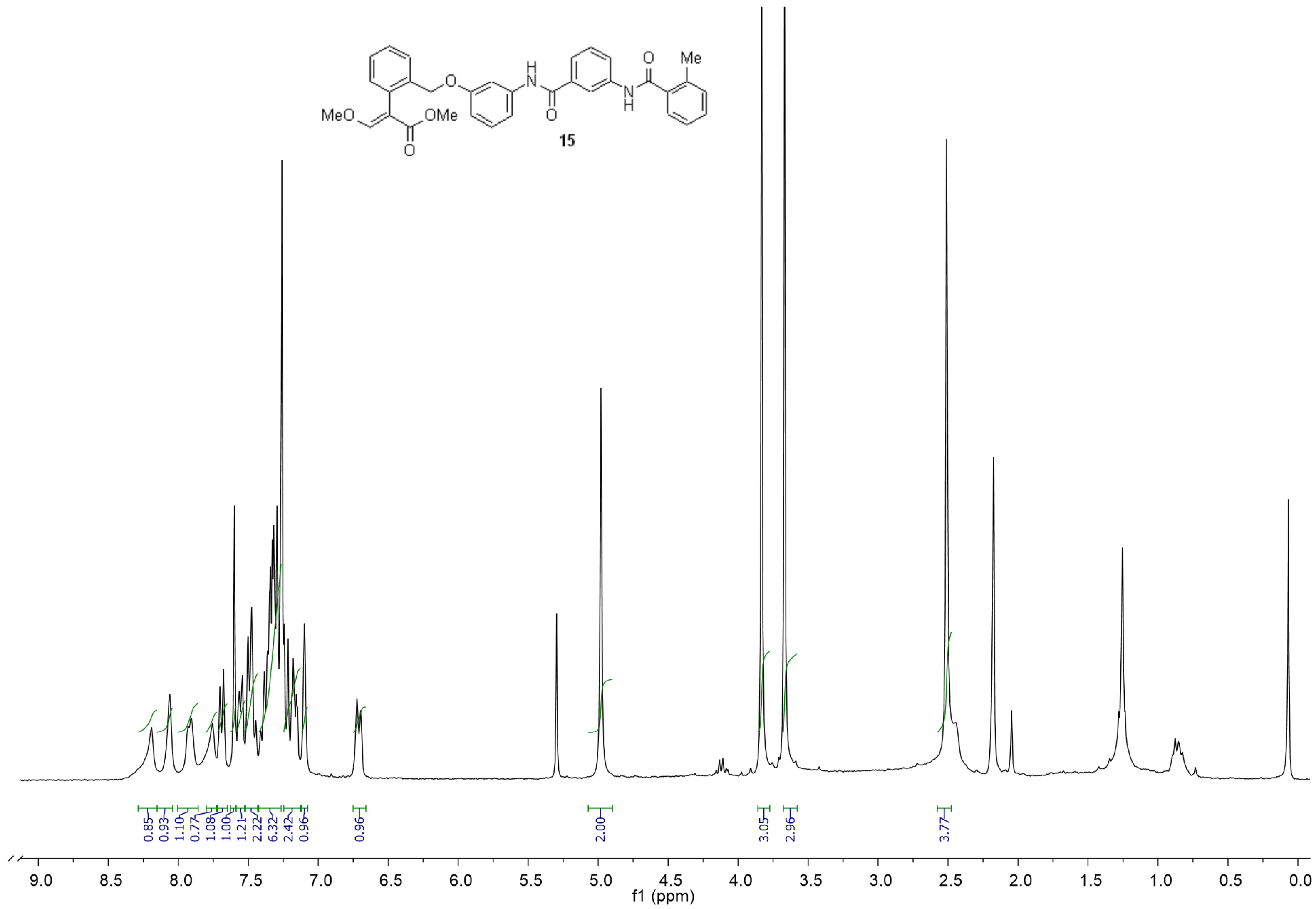

^{13}C-NMR (75 MHz, $CDCl_3$) compound **15**.

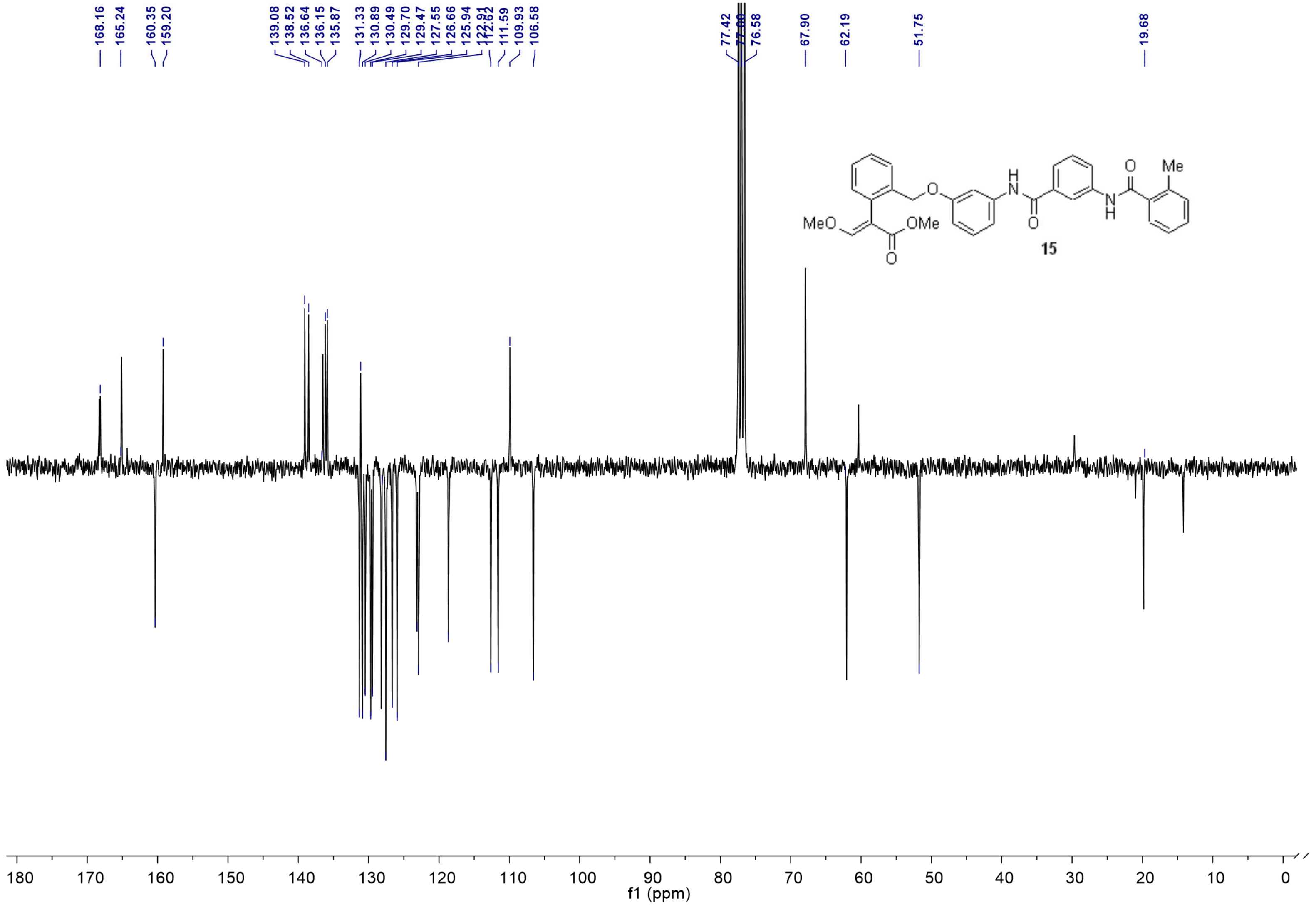

168.16
165.24
160.35
159.20
139.08
138.52
136.64
136.15
135.87
131.33
130.89
130.49
129.70
129.47
127.55
126.66
125.94
111.59
109.93
106.58
77.42
76.58
67.90
62.19
51.75
19.68
MeO
OMe
O
H
N
Me
15
180
170
160
150
140
130
120
110
100
90
80
70
60
50
40
30
20
10
0
f1 (ppm)

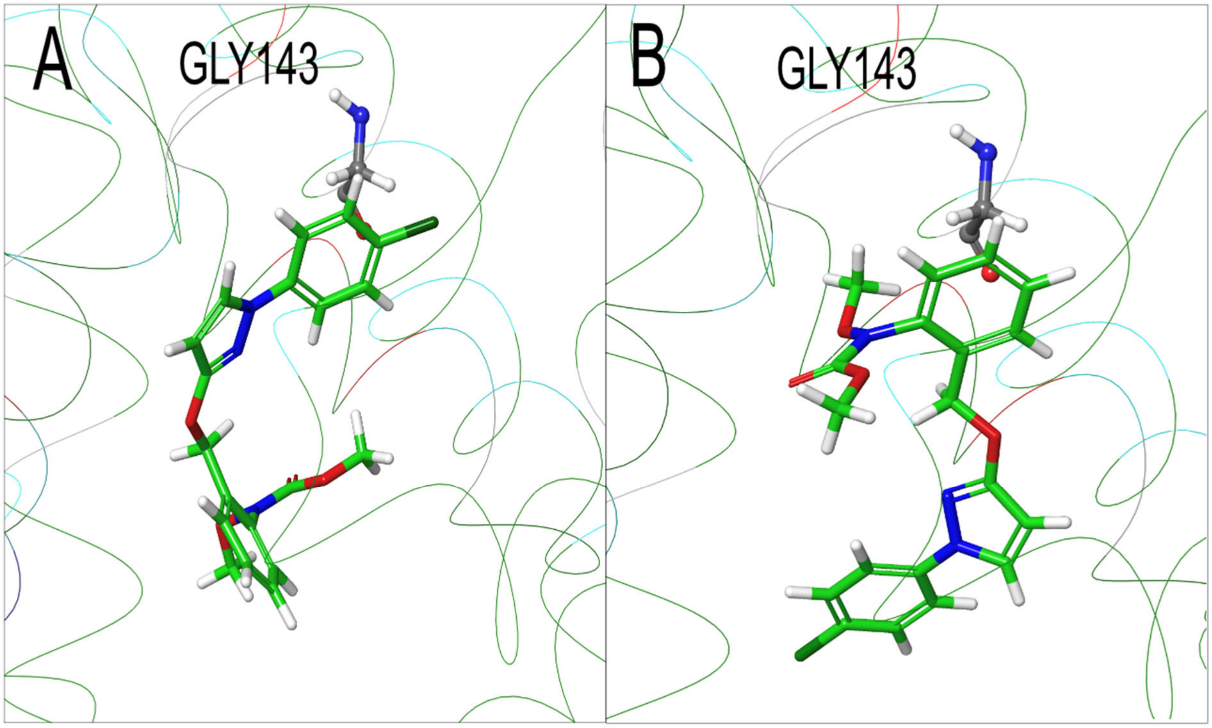

Figure S1. Top-scoring poses of pyraclostrobin. A) Top-scoring solution with a different orientation than co-crystallized azoxystrobin. B) Second top-scoring solution with a similar orientation to the co-crystallized azoxystrobin.

References [22,24,18,25,26,27,28] in main text

(1) Kim, E. M.; Jung, C. K.; Choi, E. Y.; Gao, C.; Kim, S. W.; Lee, S. H.; Kwon, O. P. Highly Conductive Polyaniline Copolymers with Dual-Functional Hydrophilic Dioxyethylene Side Chains. *Polymer (Guildf).* **2011**, *52* (20), 4451–4455. https://doi.org/10.1016/j.polymer.2011.07.052.

(2) Huang, R.; Li, Z.; Ren, P.; Chen, W.; Kuang, Y.; Chen, J.; Zhan, Y.; Chen, H.; Jiang, B. *N* -Phenyl- *N* -Aceto-Vinylsulfonamides as Efficient and Chemoselective Handles for N-Terminal Modification of Peptides and Proteins. *European J. Org. Chem.* **2018**, *2018* (6), 829–836. https://doi.org/10.1002/ejoc.201701715.

(3) Zuccolo, M.; Kunova, A.; Musso, L.; Forlani, F.; Pinto, A.; Vistoli, G.; Gervasoni, S.; Cortesi, P.; Dallavalle, S. Dual-Active Antifungal Agents Containing Strobilurin and SDHI-Based Pharmacophores. *Sci. Rep.* **2019**, *9* (1), 11377. https://doi.org/10.1038/s41598-019-47752-x.

(4) Zhou, X.; Wang, Q.; Zhao, W.; Xu, S.; Zhang, W.; Chen, J. Palladium-Catalyzed Ortho-Arylation of Benzoic Acid Derivatives via C-H Bond Activation Using an Aminoacetic Acid Bidentate Directing Group. *Tetrahedron Lett.* **2015**, *56* (6), 851–855. https://doi.org/10.1016/j.tetlet.2014.12.134.

(5) Crestey, F.; Frederiksen, K.; Jensen, H. S.; Dekermendjian, K.; Larsen, P. H.; Bastlund, J. F.; Lu, D.; Liu, H.; Yang, C. R.; Grunnet, M.; Svenstrup, N. Identification and Electrophysiological Evaluation of 2-Methylbenzamide Derivatives as Nav1.1 Modulators. *ACS Chem. Neurosci.* **2015**, *6* (8), 1302–1308. https://doi.org/10.1021/acschemneuro.5b00147.

(6) Baramov, T.; Schmid, B.; Ryu, H.; Jeong, J.; Keijzer, K.; von Eckardstein, L.; Baik, M.; Süssmuth, R. D. How Many O-Donor Groups in Enterobactin Does It Take to Bind a Metal

Cation? *Chem. – A Eur. J.* **2019**, *25* (28), 6955–6962. https://doi.org/10.1002/chem.201900453.

(7) Vilaivan, T. A Rate Enhancement of Tert-Butoxycarbonylation of Aromatic Amines with Boc_2O in Alcoholic Solvents. *Tetrahedron Lett.* **2006**, *47* (38), 6739–6742. https://doi.org/10.1016/j.tetlet.2006.07.097
